# Identification of the Acetamide-Chalcone Derivative That Selectively Inhibits ABCG2 Transport Activity

**DOI:** 10.1021/acsomega.6c07380

**Published:** 2026-09-08

**Authors:** Arthur Henrique Gomes de Oliveira, Eli Silveira Alves Ducas, Thales Kronenberger, Jean Carlos Pereira Sousa, Bruna Estelita Ruginsk, Katalin Goda, Fabiane Gomes de Moraes Rego, Geraldo Picheth, Vivian Rotuno Moure, Pablo José Gonçalves, Glaucio Valdameri

**Affiliations:** † Graduate Program in Pharmaceutical Sciences, Laboratory of Cancer Drug Resistance, 28122 Federal University of Parana, Curitiba, Paraná 80210-170, Brazil; ‡ Interfaculty Institute of Microbiology and Infection Medicine (IMIT), DZIF Tübingen Partner Site, 27203 University Hospital Tübingen, Tuebingen 72076, Germany; § Institute of Chemistry, 67824 Federal University of Goiás, Goiânia, Goiás 74001-970, Brazil; ∥ School of Pharmacy, Faculty of Health Sciences, University of Eastern Finland, Kuopio 70211, Finland; ⊥ Faculty of Pharmacy, 67824 Federal University of Goiás, Goiânia, Goiás 74605-170, Brazil; # Graduate Program in Cell and Molecular Biology, 28122 Federal University of Parana, Curitiba, Paraná 80210-170, Brazil; ∇ Department of Biophysics and Cell Biology, Faculty of Medicine, 37599 University of Debrecen, Egytem tér 1, Debrecen H-4032, Hungary; ○ Graduate Program in Pharmaceutical Sciences, 28122 Federal University of Parana, Curitiba, Paraná 80210-170, Brazil; ◆ Center of Excellence in Hydrogen and Sustainable Energy Technologies (CEHTES), Federal University of Goiás, Goiânia, Goiás 74605-170, Brazil

## Abstract

Multidrug resistance (MDR) remains a major obstacle to effective cancer chemotherapy, often driven by the overexpression of ABC transporters such as ABCG2. In this study, we identified the synthetic chalcone derivative, **B5**, as a selective ABCG2 inhibitor. Among the 20 chalcone analogs evaluated, **B5**, bearing an acetamide group at R1 and an ethoxy substituent at R2, was the only compound that produced more than 50% inhibition of ABCG2-mediated Hoechst 33342 transport at 10 μM, while showing no activity against ABCB1. **B5** exhibited an IC_50_ of 2.7 μM for ABCG2 inhibition, consistent with the low-micromolar potency reported for chalcone-based ABCG2 modulators. Furthermore, **B5** effectively reversed ABCG2-mediated resistance to the chemotherapeutic agent SN-38 in ABCG2-overexpressing cells, thereby restoring drug sensitivity. Notably, **B5** was noncytotoxic at concentrations up to 5 μM and did not exhibit differential cytotoxicity between parental and ABCG2-overexpressing cells, suggesting that it was not efficiently transported under the experimental conditions. ATPase assays revealed that **B5** stimulated, rather than inhibited, ABCG2 ATPase activity, a behavior previously reported for chemically distinct ABCG2 inhibitors. Molecular docking and molecular dynamics simulations suggested that **B5** bound within the central inhibitor-binding cavity of ABCG2, forming π–π interactions with Phe439 and hydrophobic contacts with Val546, although with a lower interaction frequency than the reference inhibitor Ko143. This binding mode, together with the physicochemical properties of **B5**, suggested that the electron-donating acetamide group enhanced its inhibitory activity, potentially by facilitating hydrogen-bonding interactions within the binding pocket. Collectively, these findings identified **B5** as a promising lead compound for the development of selective ABCG2 inhibitors and provided structural insights to support the rational design of more potent analogs capable of overcoming MDR in cancer.

## Graphical abstract

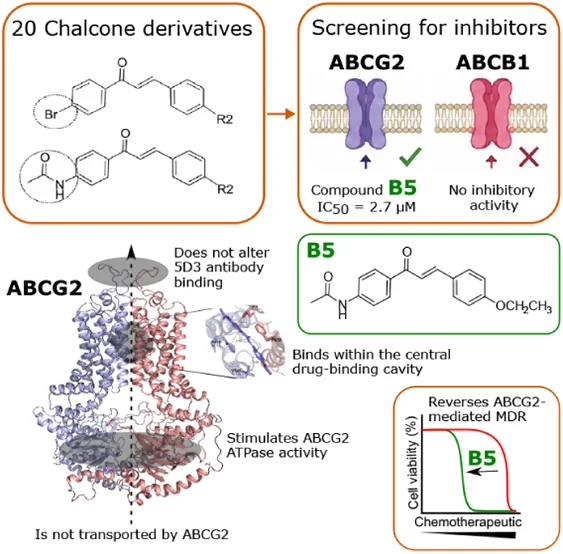

## 1 Introduction

Multidrug resistance (MDR) remains a major limitation of cancer chemotherapy and can arise through several mechanisms, including the overexpression of ATP-binding cassette (ABC) transporters that reduce the intracellular accumulation of anticancer agents.^1^ ABCG2, also known as breast cancer resistance protein (BCRP), is an ABC transporter that normally contributes to tissue protection by limiting cellular exposure to xenobiotics.^2^ However, when overexpressed in cancer cells, this protective function can reduce intracellular drug concentrations and compromise treatment response.^3^ Although numerous chemical scaffolds have been investigated as ABCG2 inhibitors, none has been clinically approved specifically to reverse ABCG2-mediated MDR, owing partly to limitations in selectivity, toxicity, pharmacokinetics, and clinical efficacy.^3,4^ Consequently, the identification of chemically accessible and structurally tunable ABCG2 inhibitors remains a priority.

Chalcones are attractive scaffolds for the development of ABCG2 inhibitors, since substituents can be independently introduced into their two aromatic rings. Chalcones have increasingly been investigated as a source of ABCB1 and ABCG2 modulators, with recent evidence indicating that inhibitory activity, transporter selectivity, and intrinsic cytotoxicity are strongly influenced by the nature and position of the ring substituents.^3^ Independent experimental studies have also shown that relatively small structural modifications can markedly alter the interaction of chalcone derivatives with ABCG2.^5,6,7^

This structural versatility provided the rationale for selecting the chalcone scaffold for the present study. Accordingly, a library of 20 chalcone derivatives was evaluated (Figure S1). Compounds A1–A10 and B1–B10 shared the same chalcone core structure and identical R2 substituents, differing only in the R1 substituent, which consisted of either an ortho-bromo or an ortho-acetamido group, respectively (see Table 1). These structural features enabled us to examine whether replacing the ortho-bromo substituent with the more polar ortho-acetamido group, which can participate in hydrogen-bonding interactions, would influence biological activity across different R2 substitution patterns. Importantly, this chalcone library was not rationally designed to generate ABCG2 inhibitors. Instead, it was repurposed in the present study as a structurally defined library for functional screening against ABCG2. The most active chalcone identified in the screening was subsequently selected for comprehensive biological characterization.

**Table 1:**
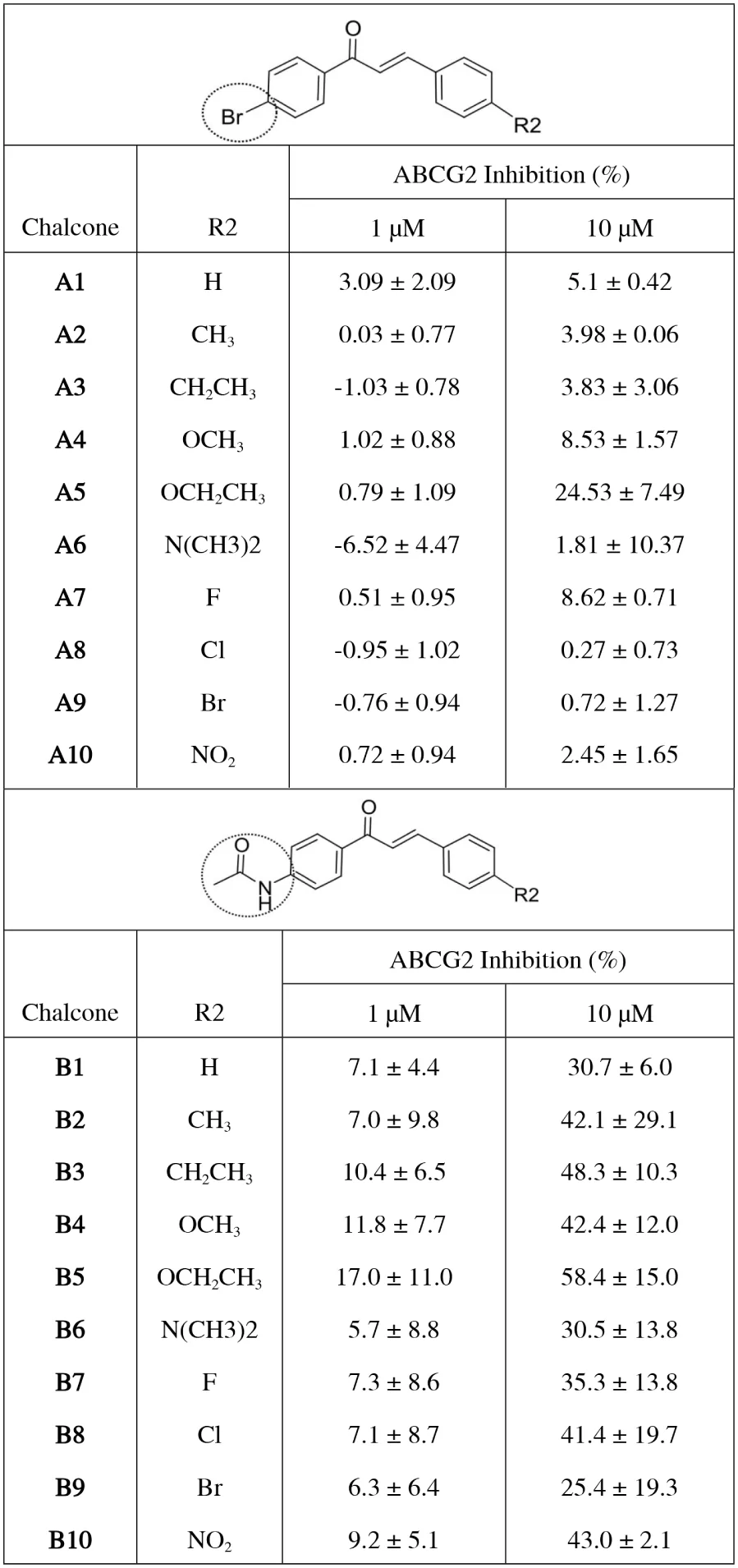
Inhibitory Effects of Chalcone Derivatives on ABCG2 Transport Activity^a^

a Description: Twenty chalcone derivatives from structural groups A and B were evaluated for their ability to inhibit ABCG2-mediated transport. The table presents the chemical structure of each derivative, including the corresponding R^2^ substituent, and the percentage of ABCG2 inhibition measured at 1 and 10 μM. ABCG2 transport activity was assessed by flow cytometry in HEK293 cells overexpressing ABCG2 and using Hoechst 33342 (3 μM) as the fluorescent substrate. Ko143 (1 μM) was used as the reference inhibitor of ABCG2 and was defined as producing 100% inhibition. Negative values indicate that no inhibitory activity or a slight stimulation of ABCG2-mediated transport was detected under the experimental conditions. Data are presented as mean ± SD from at least three independent experiments.

## 2 Materials and Methods

### 2.1 Chemistry

The chalcone derivatives employed in this study were synthesized in a single step following established procedures using commercially available reagents.^8^ The compounds were synthesized by Claisen–Schmidt condensation under basic conditions, using a protocol adapted from Nepali and colleagues.^9^ Following synthesis, the compounds were purified by recrystallization, and their structures were confirmed by ^1^H and ^13^C NMR spectroscopy and high-resolution mass spectrometry (representative structures, HRMS; Supplementary Figures S1–S30). The acetamide–chalcone derivatives evaluated in the present study are not novel compounds. They were originally synthesized and reported by Pelosi and colleagues^8^ during the investigation of their nonlinear optical, two-photon absorption, and multiphoton-excited fluorescence properties.

### 2.2 Chemicals

All chalcone derivatives were dissolved in dimethyl sulfoxide (DMSO; Sigma-Aldrich, MA, USA). Elacridar (GF120918), MK571, Ko143, and SN-38 were obtained from Sigma-Aldrich (MA, USA). The ABCG2 substrate Hoechst 33342 was purchased from Invitrogen (OR, USA). MTT (3-(4,5-dimethylthiazol-2-yl)-2,5-diphenyltetrazolium bromide) salt was acquired from VWR International (OH, USA). DMSO and ethanol, used for the solubilization of formazan crystals, were purchased from Alphatec (Brazil) and Synth (Brazil), respectively. Alexa 647 succinimidyl ester (A647) was obtained from Life Technologies, Inc. (Carlsbad, CA, USA). The 5D3 anti-ABCG2 monoclonal antibody was purified from hybridoma supernatants by affinity chromatography. The 5D3 hybridoma cell line was generously provided by Brian P. Sorrentino (Division of Experimental Hematology, Department of Hematology/Oncology, St. Jude Children’s Research Hospital, Memphis, TN, USA). The 5D3 antibody was conjugated to A647 (5D3-A647) and separated from unreacted dye by gel filtration using a Sephadex G-50 column.

### 2.3 Cell Culture

The stably transfected cell lines overexpressing human ABCG2 (HEK293-*ABCG2*)^10^ and ABCB1 (NIH3T3-*ABCB1*),^11^ as well as the corresponding parental cell lines (HEK293 and NIH3T3), were generously provided by Dr. Attilio Di Pietro (IBCP, Lyon, France). The human tumor cell lines NCI-H460 (H460) and H460MX20 (mitoxantrone-selected) were provided by Dr. Robert W. Robey and Dr. Michael Gottesman (NCI/NIH, Bethesda, MD, USA). HEK293, HEK293-*ABCG2*, NIH3T3, and NIH3T3-*ABCB1* cells were cultured in Dulbecco’s Modified Eagle Medium (DMEM; Gibco, NY, USA), whereas H460 and H460MX20 cells were cultured in RPMI 1640 medium (Gibco, NY, USA). The epidermoid carcinoma cell line A431-ABCG2, overexpressing human ABCG2 generated by retroviral transduction with an ABCG2 construct, was also used.^12^ All culture media were supplemented with 10% fetal bovine serum (FBS, Gibco, Brazil), 1% penicillin/streptomycin (Gibco, NY, USA), and 0.25 μg/mL amphotericin B (Gibco, NY, USA). Cells were kept in a humidified incubator at 37 °C under an atmosphere of 5% CO_2_.

### 2.4 ABC Transporter Inhibition Assay

ABCG2 transport inhibition assays were performed using HEK293-*ABCG2* cells. Cells were seeded at a density of 2 × 10^5^ cells per well in 24-well plates and incubated at 37 °C in a humidified atmosphere containing 5% CO_2_. After 48 h, when the cells had reached at least 80% confluence, the culture medium was removed. Cells were incubated with the reference inhibitor Ko143 (1 μM) or chalcone derivatives at concentrations of 1 and 10 μM, together with the fluorescent substrate of ABCG2, Hoechst 33342 (3 μM). After 30 min of incubation at 37 °C, the medium was discarded, and the cell monolayer was washed with prewarmed PBS (37 °C). Cells were then detached using 0.05% trypsin, resuspended in 300 μL of cold PBS, and transferred to flow cytometry tubes. Flow cytometric analysis was conducted using a FACS Celesta (BD Biosciences) equipped with a UV laser (355 nm) and a 450/50 band-pass filter. A total of 10,000 events were acquired per sample. The percentage of ABCG2 inhibition was calculated using Ko143 as the reference inhibitor, which was defined as 100% inhibition, according to the following equation:

$$Inhibition\;\left(\%\right)=\frac{\left(Cf-If\right)}{\left(RIf-If\right)}\times 100$$

whereas *Cf* represents the fluorescence of the tested chalcone derivative with the fluorescent substrate; *If* corresponds to the fluorescence only with the fluorescent substrate; and *RIf* corresponds to the fluorescence of the reference inhibitor with the fluorescent substrate. Chalcone derivatives producing more than 50% inhibition were selected for IC_50_ determination. IC_50_ values, defined as the concentration producing half-maximal inhibition of ABCG2-mediated transport, were estimated by nonlinear regression using the Michaelis–Menten equation (GraphPad Prism, V. 9.5.0). ABCB1 inhibition was investigated in NIH3T3-*ABCB1* cells stably overexpressing human ABCB1. For these assays, the reference inhibitor was elacridar (1 μM), and the fluorescent substrate was rhodamine 123 (5 μM). Cells were analyzed using a FACS Celesta (BD Biosciences) equipped with a blue laser (488 nm) and a 530/30 band-pass filter for rhodamine 123.

### 2.5 ABCG2 Conformational Antibody (5D3) Binding

The 5D3 monoclonal antibody (mAb) reactivity assay was performed using A431 cells overexpressing ABCG2 (A431-*ABCG2*). Cells were detached from culture flasks, washed three times with PBS-glucose (7 mM), and resuspended at a density of 5 × 10^5^ cells per flow-cytometry tube. Cells were preincubated with 3 or 30 μM of chalcone **B5** or 2 μM of Ko143 for 30 min at 37 °C. They were then further incubated with 2.5 μg/mL AlexaFluor-647-conjugated 5D3 antibody (5D3-A647) for an additional 30 min at 37 °C. Following incubation, cells were washed once with PBS-glucose containing 1% FBS and then once with PBS-glucose alone. The cell suspension was then incubated on ice with 5 μg/mL propidium iodide (PI). After a final wash, 20,000 events were collected on a NovoCyte flow cytometer (Agilent Technologies, Inc., Santa Clara, CA, USA) using 532 and 635 nm laser lines for excitation, and fluorescence signals were detected at 617 and 661 nm for PI and 5D3-A647, respectively. Data were analyzed using FCS Express (De Novo Software, Glendale, CA, USA), and dead cells were excluded from the analysis based on PI signal.

### 2.6 Cell Viability Assay

The intrinsic cytotoxicity and chemosensitivity assays were conducted using the MTT colorimetric method. Intrinsic cytotoxicity was assessed in HEK293 and HEK293-*ABCG2* cells. Cells were seeded at densities of 1.5 × 10^4^ (HEK293) and 2 × 10^4^ (HEK293-*ABCG2*) per well in 96-well plates and allowed to adhere for 24 h at 37 °C in a humidified atmosphere containing 5% CO_2_. After this period, the medium was removed, and cells were exposed to increasing concentrations (0.1–50 μM) of **B5** for 72 h. For the chemosensitivity assay, parental HEK293 cells and their ABCG2-overexpressing counterpart (HEK293-*ABCG2*) were used. ABCG2-overexpressing cells were pretreated with **B5**, followed by the addition of SN-38, and incubated for 72 h. Parental H460 cells and their mitoxantrone-selected, ABCG2-overexpressing counterpart H460MX20 were seeded in 96-well plates at a density of 1.5 × 10^4^ cells per well and allowed to attach for 24 h. To evaluate ABCG2-mediated drug resistance and its reversal by **B5**, cells were exposed to a fixed concentration of mitoxantrone or SN-38 (0.5 μM) in the absence or presence of **B5** (2.5 μM) or the reference ABCG2 inhibitor Ko143 (1 μM). Following treatment, MTT solution was added to a final concentration of 0.5 mg/mL, and cells were further incubated at 37 °C for 4 h. The medium was then discarded, and formazan crystals were dissolved in 100 μL of a 1:1 (v/v) DMSO:EtOH solution. Absorbance was measured at 595 nm using an iMark absorbance microplate reader (Bio-Rad, CA, USA). Untreated cells served as controls, representing 100% viable.

#### 2.6.1 Drug-Combination Analysis

Drug-combination interactions between **B5** and SN-38 were analyzed using SynergyFinder 3.0 and the Bliss independence model. The resulting Bliss δ-scores were used to classify the interaction across the tested concentration matrix, and the two-dimensional synergy map is provided in the Supporting Information.

### 2.7 Membrane Preparations for ABCG2 ATPase Activity Assay

Membrane preparation for the ATPase assay was performed following Sarkadi’s protocol with minor modifications.^13^ The entire procedure was conducted at 4 °C. A431 human epidermoid carcinoma cells overexpressing ABCG2 (A431-*ABCG2*) were detached from culture flasks and washed twice with TMEP buffer (50 mM Tris-HCl, pH 7.0; 50 mM mannitol; 2 mM EGTA; protease inhibitor cocktail; and 0.3 mM phenylmethylsulfonyl fluoride). The resulting cell pellets were resuspended in TMEP buffer and homogenized for 30 min using a glass–Teflon tissue homogenizer; this homogenization step was repeated twice. After the second homogenization, nuclei and intact cells were removed by centrifugation, and the resulting membrane-containing supernatant was centrifuged at 28,000 × g for 1 h at 4 °C. The supernatant was discarded, and the membrane pellet was resuspended in fresh TMEP buffer, homogenized again, and stored at −80 °C until use. Protein concentrations of the membrane preparations were determined using the Lowry method.^14^

### 2.8 ABCG2 ATPase Activity Assay

The vanadate-sensitive ATPase activity of ABCG2 was assessed by quantifying inorganic phosphate (Pi) release using a colorimetric assay.^38^ ^,^ All samples were prepared in assay buffer (51 mM MOPS-Tris, 64 mM KCl, 2.6 mM DTT, 0.65 mM EGTA–Tris, pH = 7.0) supplemented with 6.4 mM NaN_3_ and 1.28 mM ouabain. Membrane preparations from A431-ABCG2 cells (2.5 μg per well) were added to a 96-well plate, and half of the samples were additionally treated with 100 μM heat-activated sodium orthovanadate (Na_3_VO_4_). The membranes were then preincubated with or without Ko143 (2 μM), **B5** (3 μM or 30 μM), or SDS (5%) for 10 min at 37 °C. Following pretreatment, 3.5 mM Mg/ATP was added, and samples were incubated for another 25 min at 37 °C. The ATPase reaction was terminated by the addition of SDS, after which the color reagent (24.3 mM ammonium molybdate, 74.6 mM H_2_SO_4_, 95.2 μM potassium antimony tartrate, 0.95 M acetic acid, and 11.4 mM ascorbic acid) was added and allowed to develop for 30 min at room temperature. Finally, absorbance at 700 nm was measured using a BioTek Synergy HT plate reader (BioTek Instruments, VT, USA) at 700 nm, and the amount of P_i_ was determined.

Based on the ABCG2-specific ATPase activities measured in the presence and absence of the tested compound, the fold stimulation of ABCG2-specific ATPase activity was calculated according to equation:

$$\mathrm{Fold}\;\mathrm{stimulation}\;\mathrm{of}\;\mathrm{ATPase}\;=\frac{\mathrm{ABCG}2\;\mathrm{ATPase}\;\mathrm{activity}\;\mathrm{with}\;\mathrm{tested}\;\mathrm{compound}}{\mathrm{ABCG}2\;\mathrm{ATPase}\;\mathrm{activity}\;\mathrm{without}\;\mathrm{tested}\;\mathrm{compound}}$$

### 2.9 *In Silico* Analysis

#### 2.9.1 Model Generation

The human ABCG2 dimeric structural model was generated using the homology model suite (Maestro 2024.2, Schrödinger LCC) with standard options. Given the ligand’s scaffold similarity between our series and SN38, we chose this PDB structure 6VXJ^15^ despite its low resolution: 4.10 Å, as the model template. This structure lacks part of NBDs’ beta-sheet (Val46-Lys61) and some of the connecting loops, namely Asp301-Leu328 and Gly354-Tyr369. We retrieve the structure for those missing components from AlphaFold’s online version (accessed on 10.11.2024), generating a chimera model. We did not include the missing N-terminal (until Ala36) in our models, due to unreliability of templates and high flexibility, which were then capped. Missing loops in the original template structure were refined using Prime.^16^ Cholesterol molecules, present in the original ABCG2 cryo-EM structure, were retained. Final models were validated by checking their Ramachandran plot and overall energy levels.

For the additional ABCB1 docking analysis, the tariquidar-bound human ABCB1 cryo-EM structure (PDB ID: 7A6E) was used. The experimental structure was used directly for docking without additional homology modeling, and the transmembrane drug-binding region occupied by the cocrystallized tariquidar molecules was retained for definition of the docking site.

All protein structures were prepared using the Protein Wizard Preparation tool (Schrödinger LCC), with standard options and the homology model was further refined to remove steric clashes. For all structures protonation states of amino acids were optimized with PROPKA (pH 7.4, Schrödinger, LLC, New York, NY, 2024.2), where we selected the most likely ionization state as proposed by the software, and the structures were minimized using steep descent (cutoff for heavy atoms 1 Å).

#### 2.9.2 Molecular Docking

Compounds were drawn using Maestro and prepared using LigPrep^17^ to generate the three-dimensional conformation, adjust the ionization state to physiological pH (7.4), and calculate the partial atomic charges, with the force field OPLS4.^18^ Docking studies with the prepared ligands were performed using Glide (v7.7),^19,20^ with the flexible modality of Induced-fit docking with extra precision (XP), followed by a side-chain minimization step using Prime.^16^ Ligands were docked within a grid around the cocrystallized ligand’s centroid. For each ligand 10 docking poses were generated, using scaffold similarity to guide the docking. All docking poses were visually inspected, independently from the docking score, and those with the highest number of consistent interactions were selected for simulation. For the ABCB1 analysis, **B5** and tariquidar were independently docked into the transmembrane drug-binding cavity of the tariquidar-bound ABCB1 structure (PDB ID: 7A6E). The docking grid was positioned to encompass the region occupied by the cocrystallized tariquidar molecules, as previously described.^21^ Tariquidar was included as a reference inhibitor of ABCB1, and the same protein preparation, ligand preparation, docking, and scoring parameters were applied to both compounds. The generated poses were visually inspected to evaluate their accommodation within the transmembrane cavity and their interactions with the surrounding ABCB1 residues. Docking scores were used solely for comparative purposes and were not interpreted as direct measurements of binding affinity or inhibitory potency.

#### 2.9.3 Molecular Dynamics Simulation

Molecular dynamics simulations were carried out using Desmond^22^ engine with the OPLS4 force-field.^18^ Protein was embedded within a POPC lipid pregenerated from Maestro retaining the two original cholesterol molecules from the PDB template, using the System Builder and transmembrane alpha-helices to orient it. The system was placed in a cubic box with 13 × 13 × 13 Å from the box edges to any atom of the protein, using PBC conditions, and, filled with TIP3P^23^ water. Systems were equilibrated as previously described.^24,25^ Short-range Coulombic interactions were calculated using 1 fs time steps and a 9.0 Å cutoff value, whereas long-range Coulombic interactions were estimated using the Smooth Particle Mesh Ewald (PME) method.^26,27^ Initially, the relaxation of the system was performed using Steepest Descent and the limited-memory Broyden-Fletcher-Goldfarb-Shanno algorithms in a hybrid manner, according to the established protocol available in the Desmond standard settings. During the equilibration step, the simulation was performed under the NPT ensemble for 5 ns implementing the Berendsen thermostat and barostat methods.^28^ A constant temperature of 310 K was kept throughout the simulation using the Nose-Hoover thermostat algorithm^29^ and Martyna–Tobias–Klein Barostat^30^ algorithm to maintain 1 atm of pressure, respectively.

After minimization and relaxing steps, we proceeded with five independent 200 ns run with randomly generated seeds (in total ∼1 μs). Trajectories and interaction data are available on the Zenodo repository (10.5281/zenodo.17751261). Docking models of the ligands, binding sites, and amino acids interactions with the proteins from relevant frames of the simulation were visualized using PyMOL 3.

#### 2.9.4 MD Simulation Trajectory Analyses

Protein–ligand interactions and atomic distances were calculated using the Simulation Event Analysis pipeline as implemented in Maestro (v2024.2, Schrödinger LCC) using standard options. Representative frames of the simulations were retrieved using hierarchical clustering analyses. Trajectories were clustered using the script trj_cluster.py (implemented in Maestro 2023.2, Schrödinger LCC) according to the RMSD of the ligand’s heavy atoms, using 1 Å as the cutoff. RMSD values of the protein backbone were used to monitor simulation equilibration and protein folding changes (all raw data is available in the repository link). Protein’s backbone RMSD and RMSF were utilized to evaluate the equilibration of the system.

##### 2.9.4.1 MM/GBSA Binding Energy Calculations

Molecular mechanics with generalized Born and surface area (MM/GBSA) predicts the binding free energy of protein–ligand complexes and the ranking of ligands based on the free energy could be correlated to the experimental binding affinities, especially in a congeneric series. Every 10th frame from the simulations was considered for the calculations. These were used as input files for the MM/GBSA calculations with thermal_mmgbsa.py script from the Schrödinger package. Calculated free-binding energies are represented by the MM/GBSA and normalized by the number of heavy atoms (HAC), according to the following formula: ligand efficiency = (binding energy)/(1 + ln­(HAC)) and is expressed in kcal/mol·HAC, where HAC is the Heavy Atom Count.

#### 2.9.5 Statistical Analysis

Statistical analyses were performed using GraphPad Prism version 9.5.0 (GraphPad Software, San Diego, CA, USA). Measurements obtained within the same independent experiment were treated as matched data. For the ABCG2 ATPase activity assay, differences among treatments were evaluated using a one-way mixed-effects model fitted by restricted maximum likelihood (REML). Dunnett’s multiple-comparisons test was subsequently used to compare each treatment with the untreated basal control. For the cytotoxicity assays performed in H460 and H460MX20 cells, data were analyzed using a two-way mixed-effects model fitted by REML, including the interaction between treatment and control and accommodating missing values. Šídák’s multiple-comparisons test was applied to the prespecified comparisons between cell lines under the same treatment conditions and between selected treatments within each cell line. Concentration–response curves were fitted by nonlinear regression to estimate IC_50_ values. All statistical tests were two-sided, and multiplicity-adjusted *p* values below 0.05 were considered statistically significant. Statistical significance is indicated as follows: *p* < 0.05; *p < 0.01; **p < 0.001; ***p < 0.0001; ns, not significant. Data are presented as mean ± SD, unless otherwise stated.

## 3 Results and Discussion

### 3.1 Synthesis Scheme of Chalcones

The compounds evaluated in this study were previously reported by Pelosi and colleagues,^8^ who described their synthesis and characterization. To obtain bromo- and acetamide-substituted chalcones, a Claisen-Schmidt reaction was employed (Scheme 1). This condensation reaction between aromatic aldehydes and ketones leads to the formation of a new C–C bond, leading to α,β-unsaturated compound. The final structures were designed by fixing acetamide or bromo in orto position of the ketone portion (A), reacting with different benzaldehydes (B) with electron donor and acceptor groups.^8^ For more details, see Supplementary Figures S1–S30.

**Scheme 1:**
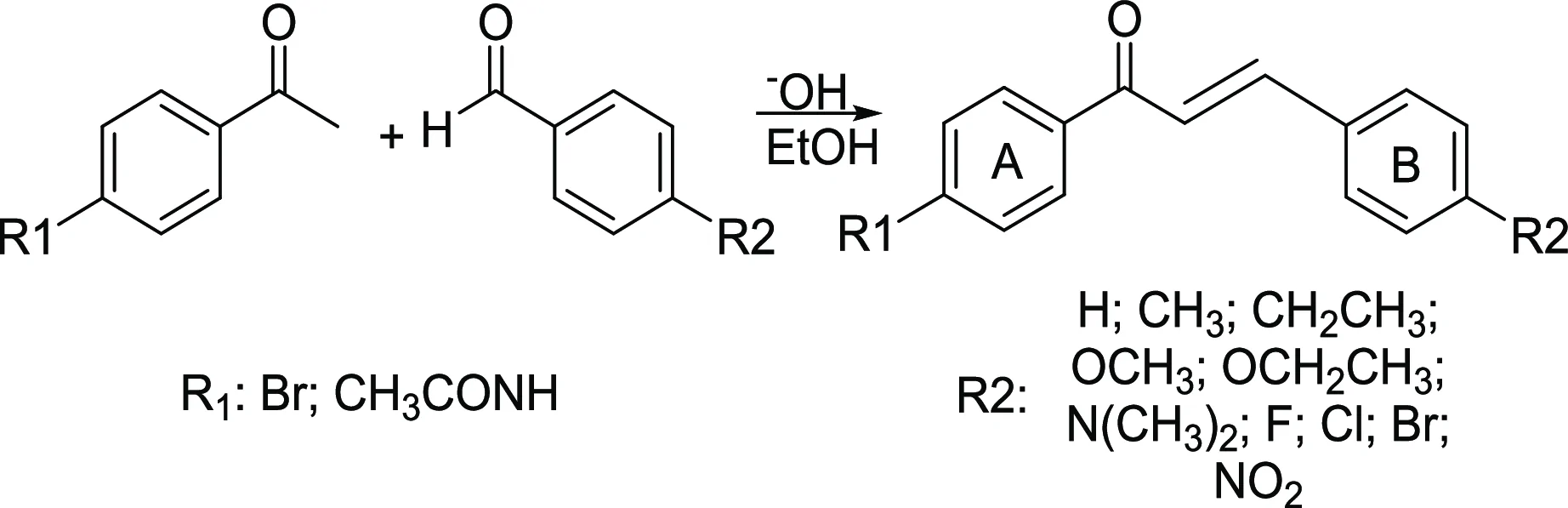
Synthesis of bromo and acetamide-chalcones with different substituents^8^

### 3.2 Screening of Chalcones for ABCG2 Inhibition

The ABCG2 inhibitory activity of the 20 chalcone derivatives (Figure S1) was initially screened using stably transfected HEK293 cells overexpressing ABCG2 (HEK293-*ABCG2*). Cells were exposed to each compound at concentrations of 1 and 10 μM, and the percentage of ABCG2 inhibition was calculated relative to the inhibition produced by the reference inhibitor Ko143. As shown in Table 1, none of the bromochalcones (group A) inhibited ABCG2 activity. In sharp contrast, most acetamide-chalcones (group B) inhibited ABCG2 activity (Table 1).

Group A of chalcones contained an ortho-bromo group as the R1 substituent, while R2 varied among the different derivatives. Overall, these compounds did not significantly inhibit ABCG2, even at 10 μM, except for **A5**, which produced a mild effect of 24% inhibition (Table 1). Thus, variation of the R2 substituent, including alkyl groups (CH_3_, CH_2_CH_3_), oxygen-containing groups (OCH_3_, OCH_2_CH_3_), or others (N­(CH3)­2, F, Cl, Br, NO_2_), did not enhance the interaction of these chalcones with ABCG2. These findings suggested that modifications at R2 alone, when R1 contained an ortho-bromo substituent, were not sufficient to confer the electronic or steric properties required for effective ABCG2 inhibition. Consistent with these findings, Winter and colleagues reported that incorporation of a quinoxaline moiety into the B-ring was shown to markedly enhance ABCG2 inhibition compared to other substituents, such as 2-naphthyl or 3,4-methylenedioxyphenyl. Furthermore, the presence of two or three methoxy groups on the phenyl A-ring was found to be critical for maximal inhibitory activity,^6^ suggesting that electron-donating groups on the A-ring improved binding interactions, while the quinoxaline unit promoted optimal fit within the transporter binding cavity.

Modifications at R2 increase the molecular bulk; however, these changes were not sufficient to promote effective ABCG2 inhibition. For example, even with the bulkier ethoxy substituent (compound **A5**), inhibition increased only modestly (24.53 ± 7.49% at 10 μM) and did not reach the levels of inhibitory activity reported for other chalcone series.^31^ Since the ortho-bromo substituent at R1 remained unchanged, the influence of R2 appeared to be overshadowed by the presence of the bromine atom. These findings suggested that the ortho-bromo substituent limited the conformational or electronic changes required to improve the interaction of these chalcones with ABCG2.

On the other hand, chalcones from group B shared the same R1 group (CH_3_CONH), whereas variations were introduced at R2. Unlike group A, these compounds exhibited greater inhibition of ABCG2 at 10 μM, with some derivatives exhibiting inhibition levels above 40% (Table 1). Several structural factors may have contributed to this increased activity. The CH_3_ONH group at R1 is an electron-donating group, which may have increased the electron density in the chalcone core, thereby enhancing its interaction with ABCG2. Previous studies have shown that substituents on the A-ring can dramatically influence the efficacy of ABCG2 inhibition.^32^ Similarly, Winter et al.^6^ reported that electron-donating groups (*e.g*., methoxy) enhanced activity. In the present series, the acetamido substituent appeared to further improve inhibitory activity, possibly because it provides both polar and hydrogen-bonding interactions.

Certain R2 substituents also appeared to influence ABCG2 inhibition. For example, the NO_2_ group in **B10** resulted in 9.2% ± 5.1 inhibition at 1 μM and 43.0% ± 2.1 inhibition at 10 μM, whereas the ethoxy (**B5**, 58.4% at 10 μM) and ethyl (**B3**, 48.3% at 10 μM) substituents were associated with even greater inhibitory activity. These findings suggested that both the electronic properties and steric characteristics of the R2 substituent influenced ABCG2 inhibition. In particular, the larger ethoxy substituent in **B5**, which produced the highest inhibition at 10 μM (58.4%), may have favored a better fit within the ABCG2 binding pocket.

Findings from Peña-Solórzano et al. 2018 demonstrated that chalcone derivatives bearing polar functional groups capable of hydrogen bonding exhibited higher selectivity and greater ABCG2 inhibitory potency.^32^ Similarly, Luciana and colleagues investigated the SAR of various chalcone inhibitors and reported that the introduction of groups capable of forming hydrogen bonds, together with favorable electronic properties, markedly improved binding interactions.^33^ Consistent with these observations, the acetamide group, by providing additional sites for polar interactions, may have contributed to the enhanced inhibitory activity observed for the acetamide-substituted chalcones.^32,33^

Based on our results, the most active derivatives from each group were **A5** and **B5**, which shared the same R2 substituent (OCH_2_CH_3_). However, **B5** exhibited significantly higher ABCG2 inhibition than **A5**, both at 1 μM (17.0% vs 0.79%) and 10 μM (58.4% vs 24.53%). These findings suggested that the difference in R1 (Br vs CH_3_CONH) was a major determinant of ABCG2 inhibitory activity. Bromine in **A5** is considered a weakly electron-withdrawing substituent and may therefore have provided limited electronic modulation of the chalcone scaffold, potentially contributing to its lower inhibitory activity. In contrast, the CH_3_ONH group in **B5** is an electron-donating substituent that may increase the electron density of the chalcone scaffold, thereby favoring interactions with ABCG2. Moreover, the R1 substituent of **B5** contains a hydrogen bond donor (−NH group), which may establish additional interactions with residues in the ABCG2 binding site, strengthening its inhibitory activity. Together, these structural features may have contributed to the higher inhibitory activity of **B5**. Because **A5** and **B5** possessed the same bulky OCH_2_CH_3_ substituent at R2, the marked difference in inhibitory activity further highlighted the importance of the R1 substituent.

The higher inhibitory activity exhibited by **B5** suggested that its balance of hydrophobicity and polarity may have been more favorable for ABCG2 inhibition. Ko143 is widely recognized as a potent and selective ABCG2 inhibitor, and its activity has been largely attributed to its carefully balanced hydrophobic and polar characteristics.^33^ This balance has been proposed to enable Ko143 to form both strong hydrophobic interactions and effective hydrogen bonds with key residues in the ABCG2 binding pocket.^33^

The CH_3_ONH group in **B5** provided additional polarity and hydrogen-bonding potential that may have favored interactions with ABCG2, resembling some of the physicochemical characteristics associated with Ko143. In contrast, bromine in **A5** is a weak electron-withdrawing atom that lacks hydrogen-bonding capability, which may have contributed to its lower inhibitory activity. Although Winter et al. primarily focused on quinoxaline-substituted chalcones, their work highlighted the importance of A-ring substituents in modulating ABCG2 inhibition. Their findings suggested the concept that fine-tuning electronic and steric properties on the chalcone scaffold could result in significant differences in inhibitory activity. Consistent with these observations, the superior performance of the acetamide-substituted chalcone **B5**, compared with its bromo-substituted counterpart **A5**, suggested that more complex multifunctional substituents can better engage the ABCG2 drug-binding site.^6^

Because chalcones were originally identified as ABCB1 inhibitors,^34^ their inhibitory activity against ABCB1 was also evaluated in stably transfected NIH3T3 cells overexpressing ABCB1 (NIH3T3-*ABCB1*). Interestingly, none of the 20 compounds inhibited ABCB1 activity (Table S1). Together, these results demonstrated that the chalcone derivatives evaluated in the present study were selective for ABCG2 (Figure S31). Because **B5** was the only derivative that produced more than 50% ABCG2 inhibition, it was selected for subsequent biological characterization.

### 3.3 Concentration-Dependent Inhibition of ABCG2-Mediated Hoechst 33342 Efflux and Reversal of ABCG2-Mediated SN-38 Resistance

To further investigate the effect of **B5** on ABCG2, a concentration–response curve was generated in HEK293-*ABCG2* cells using Hoechst 33342 as the substrate. As shown in Figure 1A, **B5** inhibited ABCG2-mediated efflux with an IC_50_ of 2.70 μM (95% confidence interval: 1.91–3.81 μM). Comparisons with previously reported inhibitors were more meaningful when the same cell-based model, substrate, and experimental conditions were employed.^35,36^ Using a similar approach (HEK293-*ABCG2* cells and Hoechst 33342 as the substrate), the chalcone derivative **16** exhibited an IC_50_ value of 1.69 ± 0.23 μM, whereas the recently described *N*-methylpyrazole derivative **1l** showed an IC_50_ of 3.80 μM. Using MDCK II cells overexpressing ABCG2 and Pheophorbide *a* as substrate, the chalcone derivative **21** showed an IC_50_ of 1.97 ± 0.18 μM.^32^ Substrate-dependent inhibition was previously demonstrated for ABCG2,^35,36^ and was consistent with the existence of multiple, partially overlapping substrate- and inhibitor-interaction sites.^36^ Consequently, the apparent inhibitory potency could vary according to the transported substrate, transport assay conditions, cell-based model, and ABCG2 expression levels.^35^ Therefore, IC_50_ values should be compared across studies with caution.

**Figure 1:**
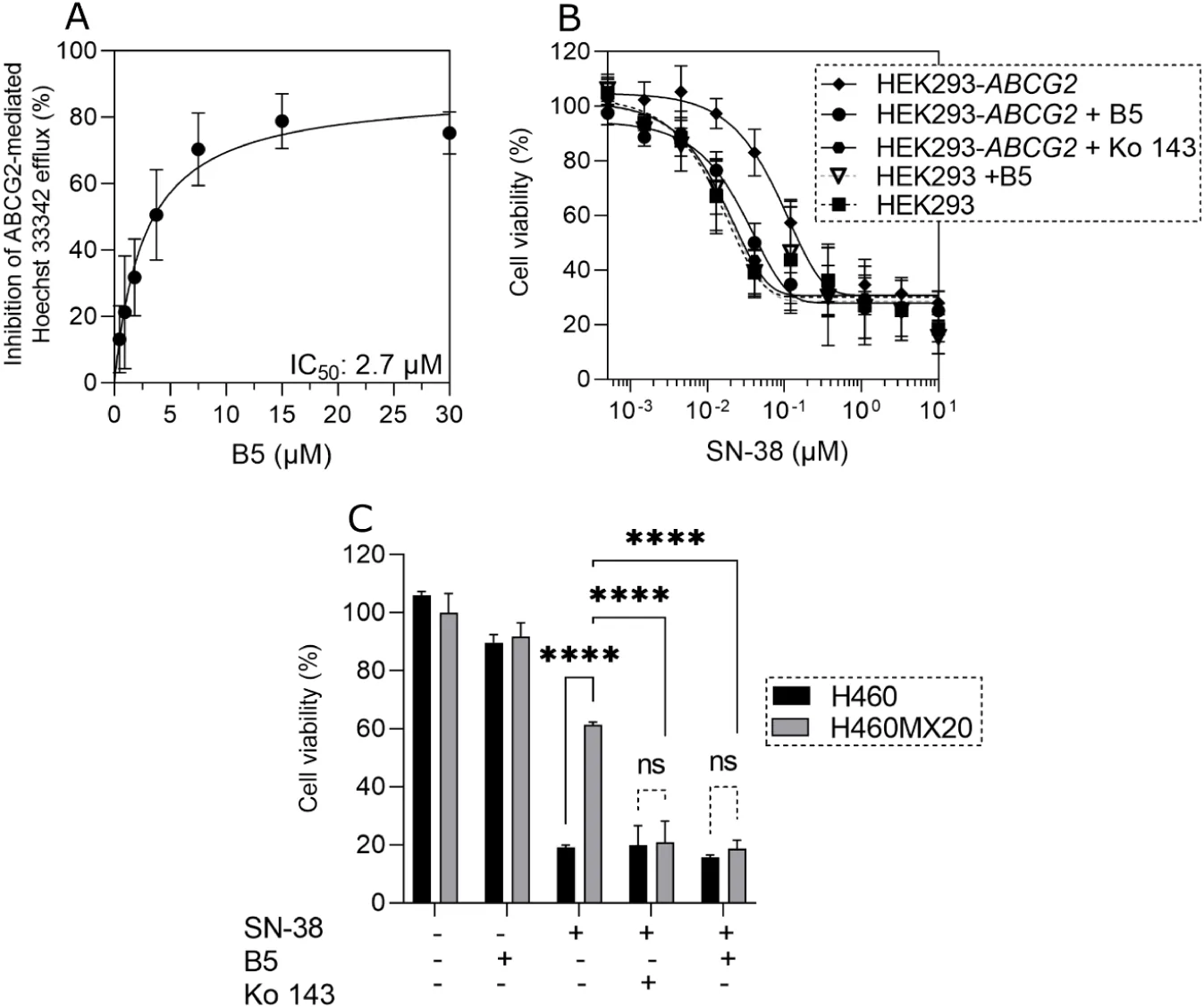
Concentration-dependent inhibition of ABCG2-mediated Hoechst 33342 efflux by **B5** and reversal of ABCG2-mediated SN-38 resistance. (A) Concentration–response curve for the inhibition of ABCG2-mediated Hoechst 33342 efflux by **B5** in ABCG2-overexpressing HEK293 cells. ABCG2 transport activity was evaluated by flow cytometry using Hoechst 33342 (3 μM) as the fluorescent substrate and Ko143 (1 μM) as the reference inhibitor, which was defined as 100% inhibition. Data were fitted by nonlinear regression using a Michaelis–Menten model. The concentration producing half-maximal inhibition (IC_50_) was 2.70 μM (95% confidence interval: 1.91–3.81 μM), and the fitted maximal inhibition was 88.23%. (B) Concentration–response curves for SN-38 cytotoxicity in parental HEK293 cells treated with SN-38 alone or in combination with **B5** (2.5 μM), and ABCG2-overexpressing HEK293 cells treated with SN-38 alone or in combination with **B5** (2.5 μM) or Ko143 (1 μM). (C) Viability of parental H460 and ABCG2-overexpressing H460MX20 cells following vehicle treatment or exposure to **B5** (2.5 μM), SN-38 (0.5 μM), SN-38 + Ko143 (1 μM), or SN-38 + **B5** (2.5 μM). Cell viability was determined by the MTT assay after 72 h of exposure. Data are presented as mean ± SD from at least three independent experiments. Data in panel C were analyzed using a two-way mixed-effects model fitted by restricted maximum likelihood (REML), including treatment, cell line, and their interaction, followed by Šídák’s multiple-comparisons test for selected comparisons. All tests were two-sided. ****, *p* < 0.0001; ns, not significant.

Given the inhibitory effect of **B5** on ABCG2, we next examined its ability to reverse the MDR phenotype associated with ABCG2 overexpression. Cell viability was evaluated in the parental HEK293 cell line and its transfected counterpart overexpressing ABCG2 (HEK293-*ABCG2*), as well as in the tumor cell line H460 and the drug-selected cell line H460MX20, which overexpresses ABCG2. The active metabolite of irinotecan (SN-38), a well-established ABCG2 substrate, was used as a cytotoxic agent.^37^ As expected, ABCG2-overexpressing cell lines were resistant to SN-38 treatment (Figure 1B,C). Notably, cotreatment of HEK293-*ABCG2* cells with 2.5 μM of **B5** restored SN-38 sensitivity to levels comparable to those observed in the parental line, indicating that this concentration of **B5** is sufficient to reverse the MDR phenotype (Figure 1B). Importantly, **B5** at 2.5 μM was noncytotoxic in both cell lines (Figure S32). A similar effect was observed in H460MX20 cells, in which **B5** restored sensitivity to SN-38 (Figure 1). To confirm that this effect was not substrate-dependent, mitoxantrone, another well-established ABCG2 substrate, was also evaluated. As shown in Figure S33, **B5** chemosensitized H460MX20 to mitoxantrone to levels comparable to those of the parental cell line H460. These findings were consistent with those reported by Yi Han and colleagues, who evaluated 17 different chalcone analogs as ABCG2 inhibitors. In their study, one of the most active chalcones at 5 μM significantly increased the intracellular accumulation of mitoxantrone and enhanced mitoxantrone sensitivity in ABCG2-overexpressing cells.^5^

An additional multidose combination analysis using the Bliss independence model yielded an overall synergy score of 10.388, indicating a moderate synergistic interaction between **B5** and SN-38. Positive δ-scores were predominantly observed at **B5** concentrations between 2 and 4 μM, which corresponded to the concentration range required for ABCG2 inhibition (Figure S34).

A promising ABCG2 inhibitor should ideally not be transported by the transporter.^38^ As shown in the Supporting Information (Figure S32), the cytotoxic effects of increasing concentrations of **B5** were evaluated in both HEK293 and HEK293-*ABCG2* cells. **B5** was noncytotoxic up to 5 μM, and no differences in cytotoxicity were observed between the two cell lines. This lack of differential cytotoxicity suggested that **B5** was not efficiently transported by ABCG2 under the experimental conditions employed. In contrast, when compounds are transported by ABCG2, distinct cytotoxicity profiles are expected between parental and ABCG2-overexpressing cells, as illustrated by the findings of Lydia Kuhnert and colleagues, in which mitoxantrone produced pronounced differences in cytotoxicity between ABCG2-overexpressing and parental cells,^39^ as well as by the differential sensitivity to SN-38 observed in the present study (Figure 1B). However, additional studies using more sensitive analytical approaches, such as LC–MS/MS, will be required to definitively determine whether **B5** is transported by ABCG2.

Previous studies demonstrated that the inhibitory potency of chalcone derivatives toward ABCG2 was highly dependent on the substitution pattern of the A and B rings, with several compounds exhibiting submicromolar or low-micromolar IC_50_ values.^7,31^ Some chalcone derivatives, such as **9** and **13**, showed selective ABCG2 inhibition but were associated with significant cytotoxicity.^33^ In contrast, **B5** exhibited low intrinsic cytotoxicity while effectively reversing ABCG2-mediated drug resistance. Although the previously reported chalcone derivative **10** exhibited greater inhibitory potency (IC_50_ = 0.34 μM) and low cytotoxicity (>50 μM),^31^ **B5** also effectively chemosensitized ABCG2-overexpressing cells, highlighting its potential as a promising lead compound for the development of agents capable of overcoming ABCG2-mediated MDR.

### 3.4 Effects of B5 on the 5D3 Shift and ABCG2 ATPase Activity

The transport of substrates across the cellular membrane by ABC transporters is driven by ATP binding and hydrolysis.^40^ Substrate transport is generally associated with stimulation of ATPase activity, whereas many ABC transporter inhibitors reduce ATP hydrolysis.^41^ For ABCG2, quercetin has been reported as a known ATPase stimulator, while Ko143 inhibited the ABCG2-specific activity (Figure 2). In contrast to the effect of Ko143, chalcone derivative **B5**, at concentrations close to its IC_50_ (∼3 μM) and at a 10-fold higher concentration, stimulated ABCG2 ATPase activity to approximately twice the basal level. Although most known inhibitors suppress ATP hydrolysis of ABC transporters,^42^ this is not universally observed. Several structurally distinct compounds, including sildenafil,^43^ curcumin analogs,^44^ imatinib,^45^ indeno­[1,2-*b*]­indole,^46^ and chromone,^47^ have also been reported to stimulate ABCG2 ATPase activity while inhibiting substrate efflux. This apparent paradox reflects that **B5** might act as a “fake substrate”; it engages the transporter and promotes ATP turnover, but instead of being efficiently translocated, it blocks the transport cycle.

**Figure 2:**
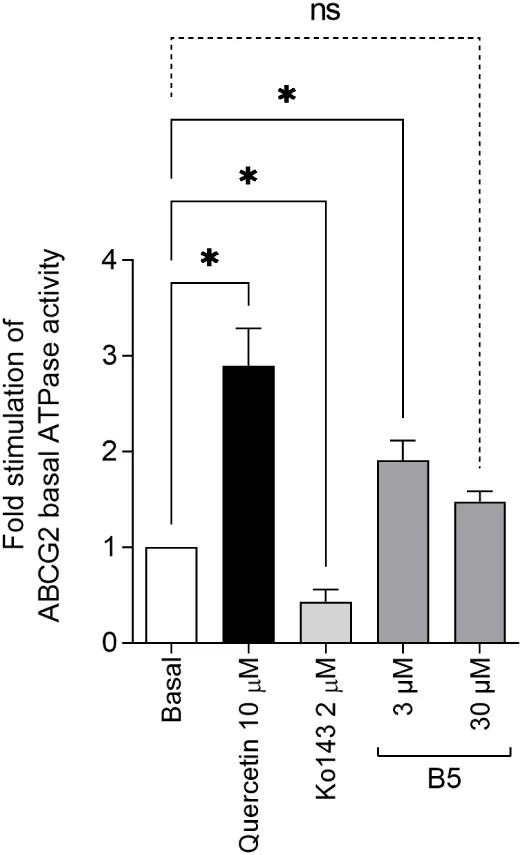
Effect of **B5** on ABCG2 ATPase activity. Vanadate-sensitive ABCG2 ATPase activity was measured using a colorimetric assay in membrane preparations obtained from A431 cells overexpressing ABCG2. Untreated membranes were used to determine basal ATPase activity (white bar). Quercetin (10 μM; black bar) and Ko143 (2 μM; light-gray bar) were included as substrate and inhibitor controls, respectively. **B5** was evaluated at a concentration close to its previously determined IC_50_ (3 μM) and at a 10-fold higher concentration (30 μM). ATPase activity was normalized to the untreated basal control and expressed as fold stimulation. Data are presented as mean ± SD from three independent experiments, except for **B5** at 30 μM (*n* = 2). Differences among treatments were analyzed using a one-way mixed-effects model fitted by restricted maximum likelihood (REML), followed by Dunnett’s multiple-comparisons test comparing each treatment with the basal control. All tests were two-sided. *p* < 0.05; ns, not significant.

To better understand the behavior of **B5**, we further evaluated the effect of this compound on the binding of the conformation-sensitive 5D3 antibody, which recognizes an extracellular epitope of ABCG2. Increased 5D3 reactivity is commonly used as an indicator of potent ABCG2 inhibition. Inhibitors such as Ko143 typically induce a 3- to 5-fold increase in 5D3 binding. However, as shown in the Supporting Information (Figure S35), **B5** did not alter 5D3 antibody binding, a finding also observed for the indeno­[1,2-*b*]­indole derivatives, suggesting that some ABCG2 inhibitors do not promote conformational changes that affect the 5D3 binding.^46^ Collectively, these findings suggested that effective inhibition of ABCG2-mediated transport does not necessarily require suppression of ATPase activity or affect the 5D3 binding.

### 3.5 Molecular Dynamics Simulations of ABCG2 in the Presence of Ko143 and **B5**

To better investigate the binding mode of **B5** within ABCG2, we used a combination of molecular docking followed by molecular dynamics (MD) simulations. We initially positioned **B5** and Ko143 within the central inhibitor-binding cavity, located between the two subunits and centered around the twin Phe439 residues. A single representative docking pose was then selected for each compound for subsequent MD simulations. Chalcone derivative **B5** adopted a binding orientation characterized by π-π interactions with the Phe439 residues, with higher interaction frequency for Phe439A than B (47.3% vs 11.6%) (Tables S2,S3).

The selected docking poses underwent short MD simulations (5 × 200 ns), during which the protein reached equilibrium after 50 ns (protein RMSD < 3 Å). The ligand conformations, however, exhibited only minor fluctuations throughout the simulations, as reflected by Lig. RMSD values of approximately 1–1.5 Å (Figure S36), indicating that the initial docking pose remained stable during the MD simulations. The corresponding MM/GBSA binding free-energy and ligand-efficiency distributions are shown in Figure S37. For both **B5** and Ko143, the binding modes were primarily stabilized by π-mediated interactions (47.3% and 40.9%, respectively; Table S3). However, only **B5** interacted with Phe439B through a π-mediated interaction (11.6%, Table S3), which may have contributed to its lower binding stability and inhibitory activity. In addition, the binding of Ko143 was further stabilized by H-bond interactions with Gln398 (Table S4) and hydrophobic interactions with Phe439 (chain A, 44%), Val 546 (B, 49.5%) (Table S2).

Based on the MD simulation data, we proposed that **B5** adopted a binding orientation in which the acetoamide (R1) moiety was positioned near the aromatic residues Phe439A and Phe439B. The first aromatic ring of the chalcone established contacts with these residues at distances of 3.7 Å and 4.1 Å, consistent with potential dipole–dipole or π interactions. In contrast, the ethoxy group (R2) was oriented toward Val546A, maintaining a contact distance of approximately 3.5 Å. Additionally, hydrophobic interactions with Val546A and Val546B may have further contributed to the stability of the complex, supporting this orientation as the most plausible binding mode for the chalcone scaffold (Figure 3B and Table S2).

**Figure 3:**
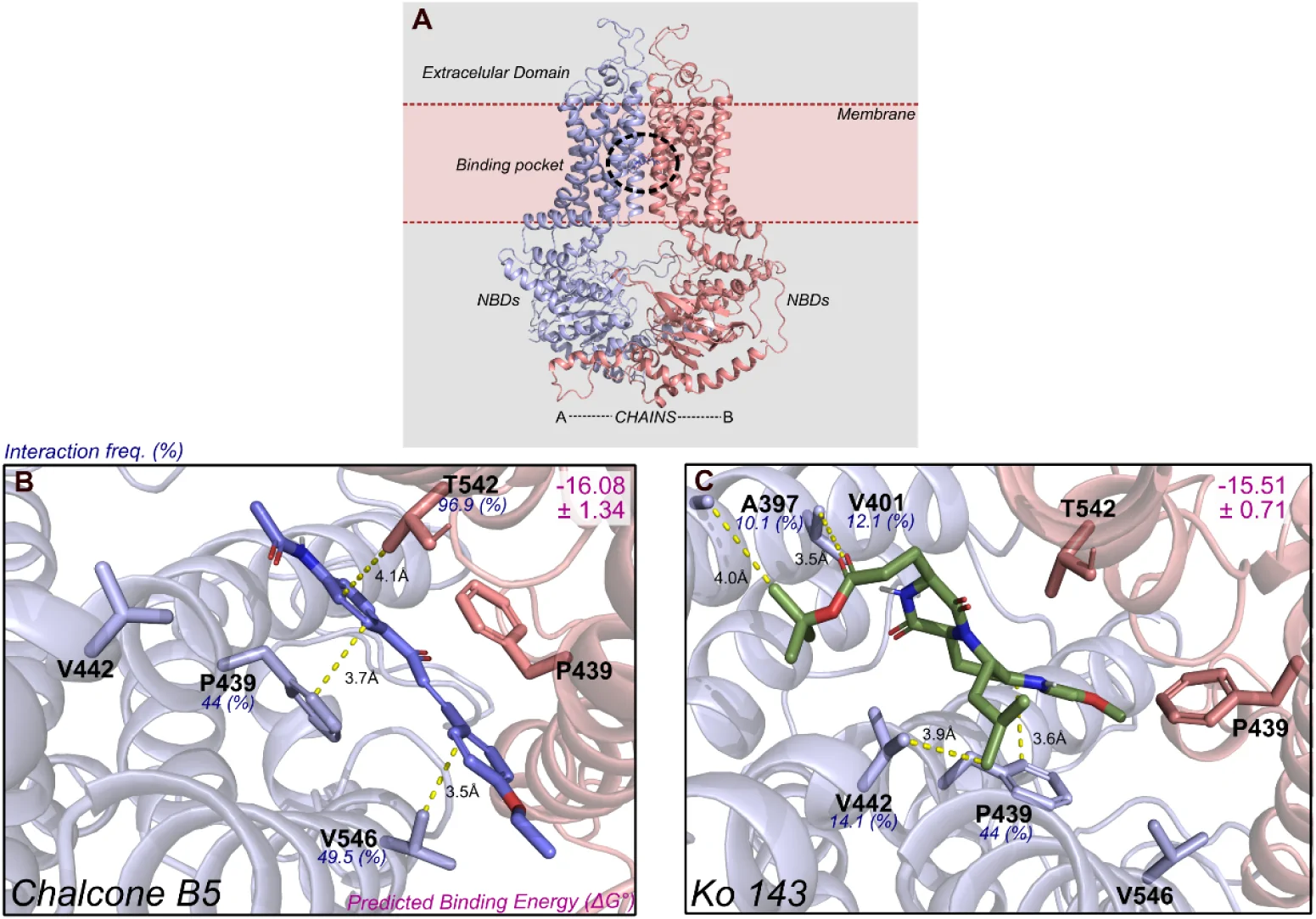
Predicted binding modes and molecular interactions of Ko143 and B5 with ABCG2. (A) Cartoon representation of the ABCG2 homodimer embedded in a membrane environment, with chains A and B shown in light blue and salmon, respectively. The nucleotide-binding domains (NBDs) and the extracellular side of the transporter are indicated. (B–C) Representative binding poses of **B5** (blue; B) and the reference ABCG2 inhibitor Ko143 (green; C), obtained from the molecular dynamics simulations within the transmembrane drug-binding cavity. Hydrogen bonds and π–π interactions are represented by dashed lines, with distances reported in Å. The corresponding MM/GBSA binding free-energy values are shown in purple and expressed in kcal·mol^–1^.

On the other hand, the binding pose of Ko143 showed a preference for chain A over chain B of ABCG2. Unlike **B5,** which adopted a sandwich-like conformation between Phe439A and Phe439B, Ko143 adopted a more vertically stacked orientation. This binding mode was characterized by π stacking interactions with Phe439A at a distance of 3.6 Å, with an interaction frequency of 40.9%, whereas no interaction with Phe439B was observed. Additionally, Ko143 formed more hydrophobic interactions than **B5**, particularly with residues Ala397A, Val401A, Val442A, and Val442B (Table S2). These additional interactions may have been attributed to the larger molecular size of Ko143 compared with **B5**. Based on this binding mode, chalcone derivative **A5** should not encounter steric hindrance in fitting within the cavity. However, the presence of bromine atoms, which have a lower capacity for hydrogen bonding compared to the acetoamide substituent of **B5**, may have resulted in a less stable binding conformation and weaker inhibition of ABCG2.

Interestingly, both inhibitors interacted with residues distributed across transmembrane helices (TMH) 2 and 5. However, Ko143 bound to the inward-facing cavity of ABCG2, thereby interrupting conformational transition, blocking substrate and preventing the conformational changes required for ATP hydrolysis.^48^ In contrast, **B5** stimulated ATPase activity. MD simulations suggested that Ko143 more effectively stabilized the cavity interactions involving Phe439A, Gln398, and an extensive hydrophobic network, consistent with its ATPase inhibition activity. **B5**, on the other hand, engaged the binding site asymmetrically with less persistent contacts, which may have favored nonproductive catalytic cycles and thereby explained the stimulation of ATPase activity.

To further investigate the lack of ABCB1 inhibition by **B5** (Table S1), an additional molecular docking analysis was performed using the tariquidar-bound human ABCB1 structure (PDB ID: 7A6E). Tariquidar exhibited a more favorable docking score than **B5** (−10.925 and −7.659, respectively; Table S5). Although **B5** was predicted to fit within the transmembrane drug-binding cavity of ABCB1, its less favorable binding score and smaller molecular size may have limited its ability to reproduce the binding arrangement and conformational stabilization associated with tariquidar-mediated inhibition. Nevertheless, these findings should be interpreted with caution, as docking scores represent only comparative predictions and were not sufficient to explain the absence of ABCB1 inhibition observed in the functional assay.

## 4 Conclusion

In conclusion, this study identifies the acetamide–chalcone derivative **B5** as a selective inhibitor of the ABCG2 transporter capable of effectively reversing ABCG2-mediated MDR. Under the experimental conditions employed, **B5** exhibited no differential cytotoxicity between parental and ABCG2-overexpressing cells, suggesting that it was not efficiently transported by ABCG2 while maintaining the ability to stimulate ATPase activity. Molecular docking and molecular dynamics simulations proposed a putative binding mode for **B5** within ABCG2 and provided valuable structural insights that may guide the rational design of more potent chalcone analogues. Collectively, these findings establish **B5** as a promising lead compound for the development of selective ABCG2 inhibitors aimed at overcoming MDR in cancer.

## Supplementary Material



## Notes

Experimental procedures, synthesis, and structural characterization of chalcone derivatives; NMR and HRMS spectra; additional data on ABCG2 and ABCB1 transport inhibition, cytotoxicity, resistance reversal, Bliss interaction, and 5D3-shift assays; molecular dynamics analyses; MM/GBSA binding free-energy calculations; protein–ligand interaction frequencies; and ABCB1 docking results (PDF)
