# Supplementary material for: Identification of the Acetamide-Chalcone Derivative That Selectively Inhibits ABCG2 Transport Activity
Source: ACS Omega. 2026 Sep 8;11(37):56373–83. doi: 10.1021/acsomega.6c07380 (PMC13613470; doi:10.1021/acsomega.6c07380)
Supplement: Supplementary file 1 [file ao6c07380_si_001.pdf]

SUPPORTING INFORMATION

Identification of the acetamide-chalcone derivative that selectively inhibits ABCG2 transport activity

Arthur Henrique Gomes de Oliveira[1,2], Eli Silveira Alves Ducas[3], Thales Kronenberger[2,4], Jean Carlos Pereira Sousa[5], Bruna Estelita Ruginsk[6], Katalin Goda[7], Fabiane Gomes de Moraes Rego[8], Geraldo Picheth[8], Vivian Rotuno Moure[1], Pablo José Gonçalves[3,9*] and Glaucio Valdameri[1,6 *]

[1]*Graduate Program in Pharmaceutical Sciences, Laboratory of Cancer Drug Resistance, Federal University of Parana, Curitiba 80210-170, PR, Brazil*
[2]*Interfaculty Institute of Microbiology and Infection Medicine (IMIT), DZIF Tübingen partner site. University Hospital Tübingen, Tuebingen, Germany*

[3]*Institute of Chemistry, Federal University of Goiás, Goiânia 74001-970, GO, Brazil.*

[4]*School of Pharmacy, Faculty of Health Sciences, University of Eastern Finland, 70211, Kuopio, Finland*
[5]*Faculty of Pharmacy, Federal University of Goiás, Goiânia 74605-170, GO, Brazil*
[6]*Graduate Program in Cell and Molecular Biology, Federal University of Parana, Curitiba, PR, Brazil*

[7]*Department of Biophysics and Cell Biology, Faculty of Medicine, University of Debrecen, Egytem tér 1, Debrecen, H-4032, Hungary*
[8]*Graduate Program in Pharmaceutical Sciences, Federal University of Parana, Curitiba 80210-170, PR, Brazil*
[9]*Center of Excellence in Hydrogen and Sustainable Energy Technologies (CEHTES), Federal University of Goiás, Goiânia, GO, Brazil*

*Correspondence: Pablo José Gonçalves (Email: pablo@ufg.br) and Glaucio Valdameri (Email: gvaldameri@ufpr.br)

1. Synthesis and characterization of chalcones derivatives.

The series of ten bromo (Group A) and acetamide-chalcones (Group B) was obtained (Fig. S1) through a Claisen–Schmidt condensation, employing a same protocol used to synthetize the acetamide-chalcones as described in [1]. Briefly, 4′-bromoacetophenone (1 mmol) and the corresponding benzaldehyde (1 mmol) were dissolved in a 5% NaOH/ethanol solution in a three-neck round-bottom flask and maintained under constant stirring at room temperature overnight. All reagents and solvents were used as supplied by Sigma-Aldrich (San Luis, USA), Acros Organics (Thermo Scientific, San Jose, USA), and Neon (Suzano, Brazil) without further purification.

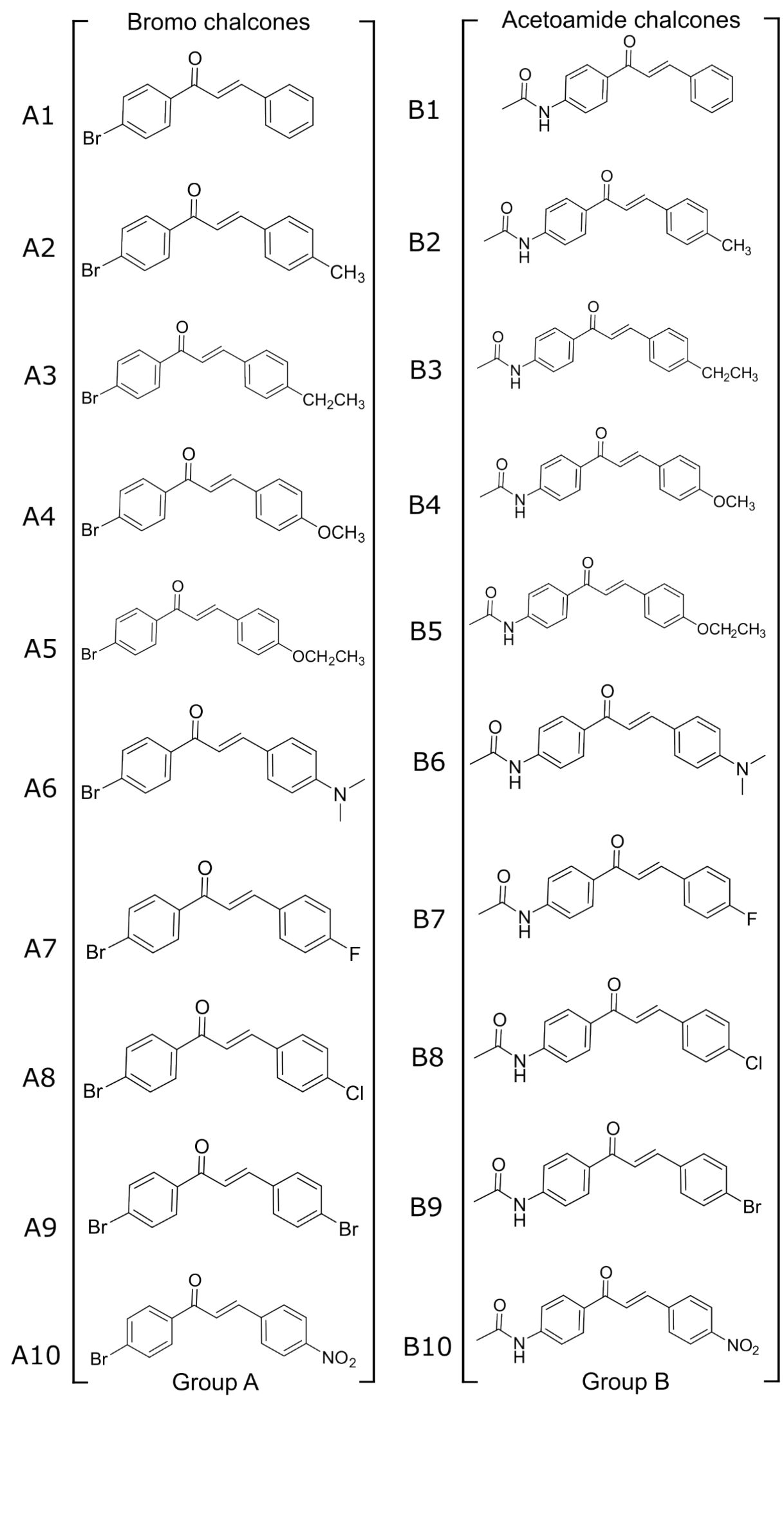

Figure S1. Representative structures of chalcones. Chemical structures of the synthesized chalcone derivatives evaluated in this study. Compounds are displayed from left to right and top to bottom in the following order: A1-A10/B1-B10. Each structure depicts the chalcone core scaffold with specific substituents (R_1, R_2) indicated, corresponding to the chemical modifications investigated for their impact on ABCG2 inhibition.

Reaction progress was assessed by thin-layer chromatography employing hexane/ethyl acetate mixtures (85:15 or 80:20, v/v) as the mobile phase. Following completion, hydrochloric acid was added in equimolar proportion to neutralize the reaction medium, resulting in precipitation of the crude products. The solids were isolated by vacuum filtration, washed with ice-cold water (6 °C), and subsequently purified by recrystallization using either hexane/ethyl acetate (60:40, v/v) or methanol.

Structural confirmation was made by ^{1}H and ^{13}C Nuclear Magnetic Resonance (NMR) spectroscopy and High-resolution mass spectrometry (HRMS).

- **1.1 NMR methodology**

The NMR recorded on Bruker Avance III 500MHz (11,75 T) spectrometer (Bruker Corporation, Billerica, USA) and the samples were solubilized in deuterated dimethylsulfoxide (DMSO-*d6* – Sigma Aldrich, San Luis, USA) and deuterated chloroform ($CDCl_3$ – Cambrigde Isotope Laboratories, Teksburry, USA).

- **1.2 Mass spectrometry methodology**

Methanol (≥99.9%) and acetonitrile (≥99.9%) were obtained from LiChrosolv® Sigma-Aldrich (St. Louis, USA), and formic acid (≥95%) was purchased from Sigma-Aldrich (St. Louis, USA). The dried extract was reconstituted in 1 mL of a methanol–acetonitrile mixture (2:1, v/v), after which 10 µL of formic acid was added to enhance ionization efficiency under positive electrospray conditions.

Electrospray ionization mass spectrometry (ESI-MS) analyses were performed using a high-resolution hybrid Quadrupole-Orbitrap mass spectrometer (Q-Exactive™, Thermo Scientific). This instrument was selected for its capacity to provide resolutions above 140,000 (FWHM at *m/z* 200) and mass accuracy below 3 ppm, both of which are essential for the reliable identification of molecular species in complex matrices.

Ionization parameters were adjusted to promote efficient desolvation and stable signal generation. A spray voltage of +3.8 kV was applied, the capillary temperature was

maintained at 320 °C to prevent thermal degradation of labile analytes, and nitrogen gases of ≥99.9% purity were regulated at 8 L min^{-1} (sheath) and 5 L min^{-1} (auxiliary) to optimize nebulization and ion transfer. The S-lens potential was set to 45 V to ensure effective ion focusing with minimal collisional loss.

For Tandem mass spectrometry (MS/MS) structural characterization, fragmentation was performed in the HCD cell using collision-induced dissociation under normalized collision energies of 20, 25, and 30 eV. This gradient-based approach allows systematic evaluation of energy-dependent fragmentation pathways, including characteristic neutral losses and rearrangements, thereby facilitating structural confirmation and discrimination of potential isomers. The controlled variation in collision energy also improves precursor-to-fragment transition selection, supporting more robust qualitative interpretation and, when applicable, Multiple Reaction Monitoring (MRM-based) quantification.

- **1.3 NMR spectra**

1H NMR spectra of all synthesized compounds exhibited the characteristic pair of doublets associated with trans-chalcones, with coupling constants of approximately 15 Hz. These resonances, observed near 7.4 and 7.7 ppm, correspond to the α,β-olefinic protons in the E configuration.

For chalcone $ChCH_3$ (Fig. SI6), two additional doublets (2H each) were detected at 7.20 and 7.51 ppm (J = 8.0 Hz), assigned to the aromatic protons attached to C3/C5 and C2/C6, respectively. Signals at 7.66 and 7.99 ppm were attributed to protons bonded to the C3′/C5′ and C2′/C6′ aromatic carbons. Two singlets at 2.20 and 2.37 ppm (3H each) correspond to the methyl groups at C8 and C9. The amide N–H proton appeared as a broad singlet at 7.84 ppm.

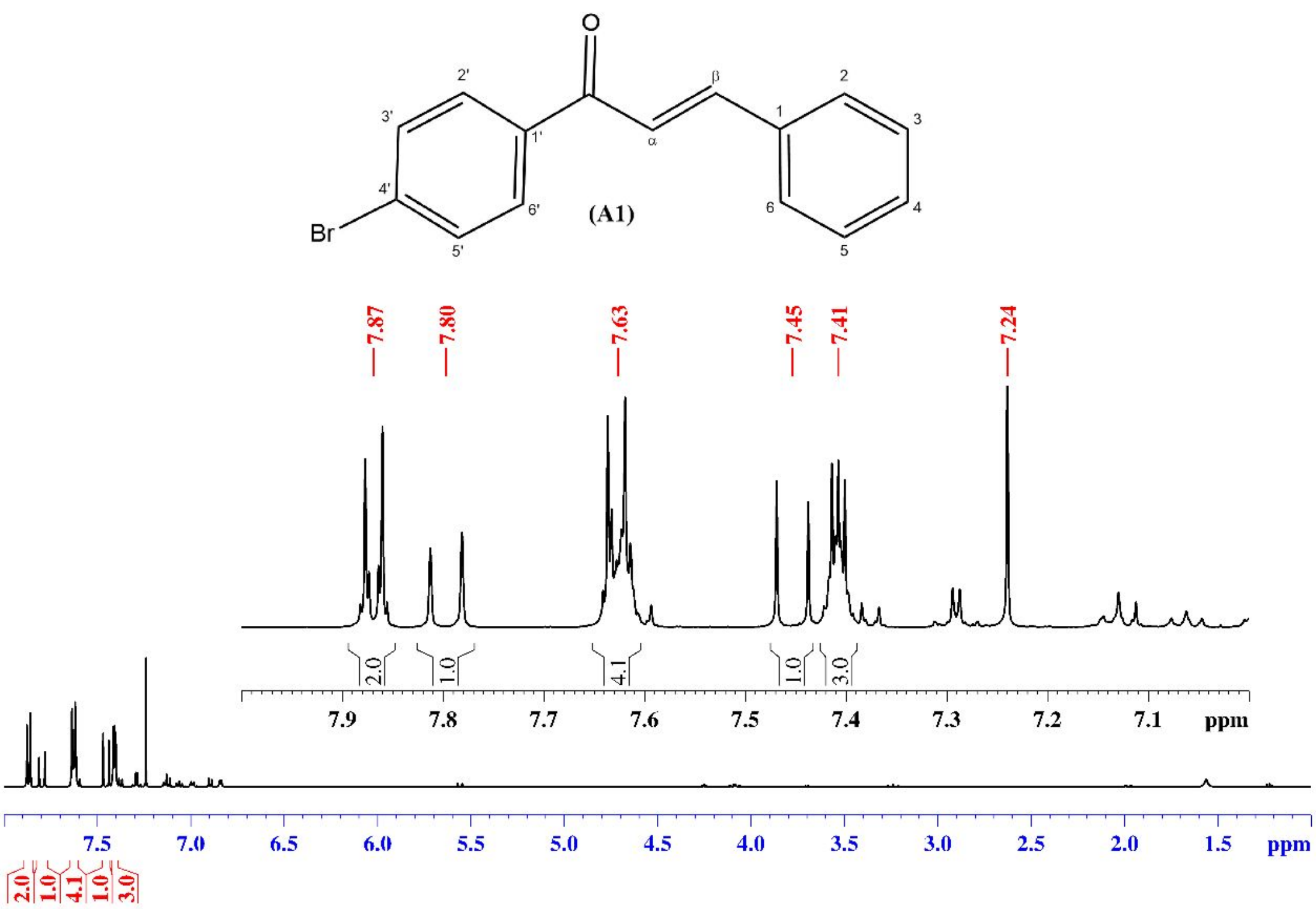

Figure S2. ^{1}H NMR spectra, in CDCl3 (7.24 ppm), of **A1**.:

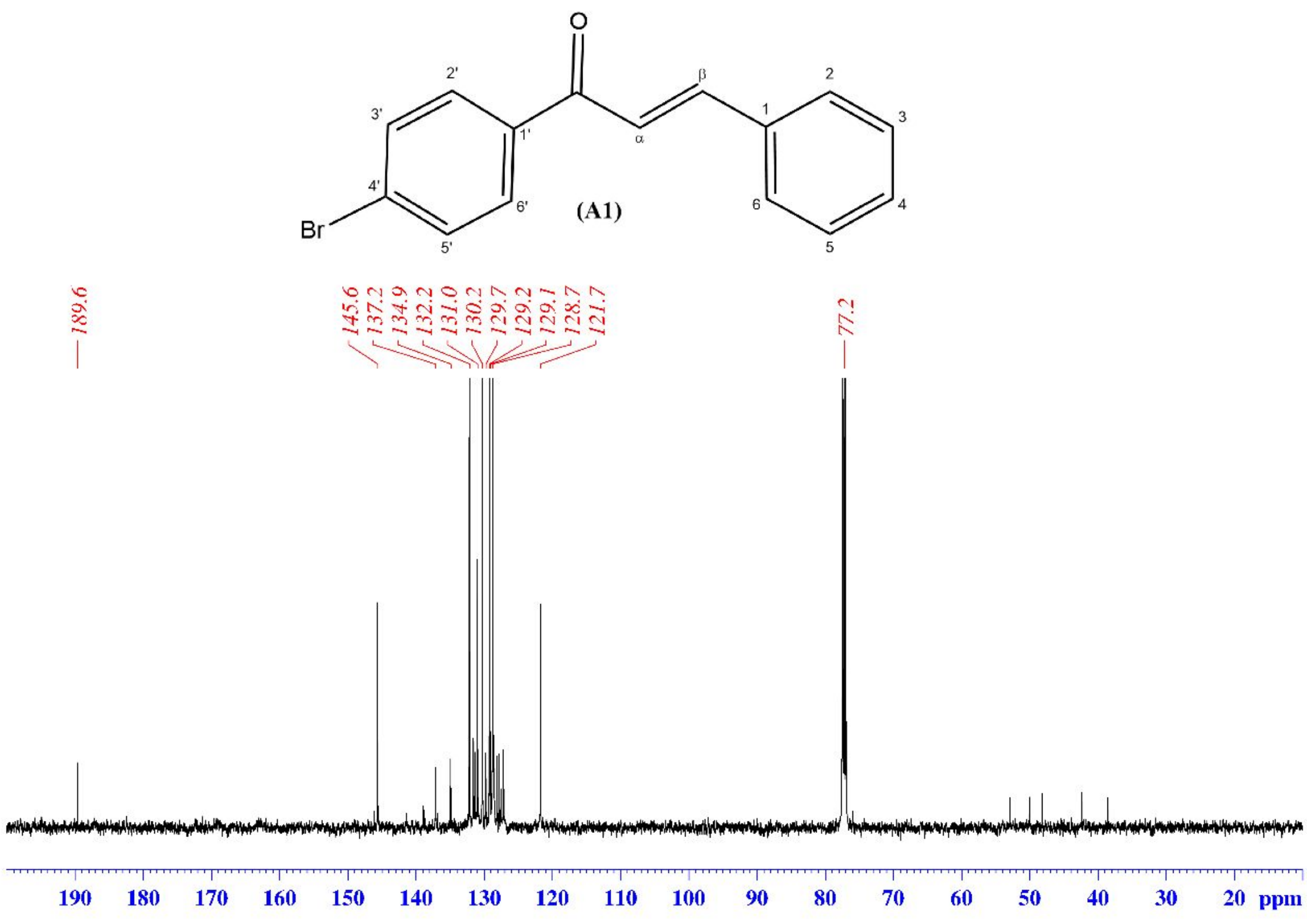

Figure S3. ^{13}C NMR spectra, in CDCl3 (77.2 ppm), of **A1**.

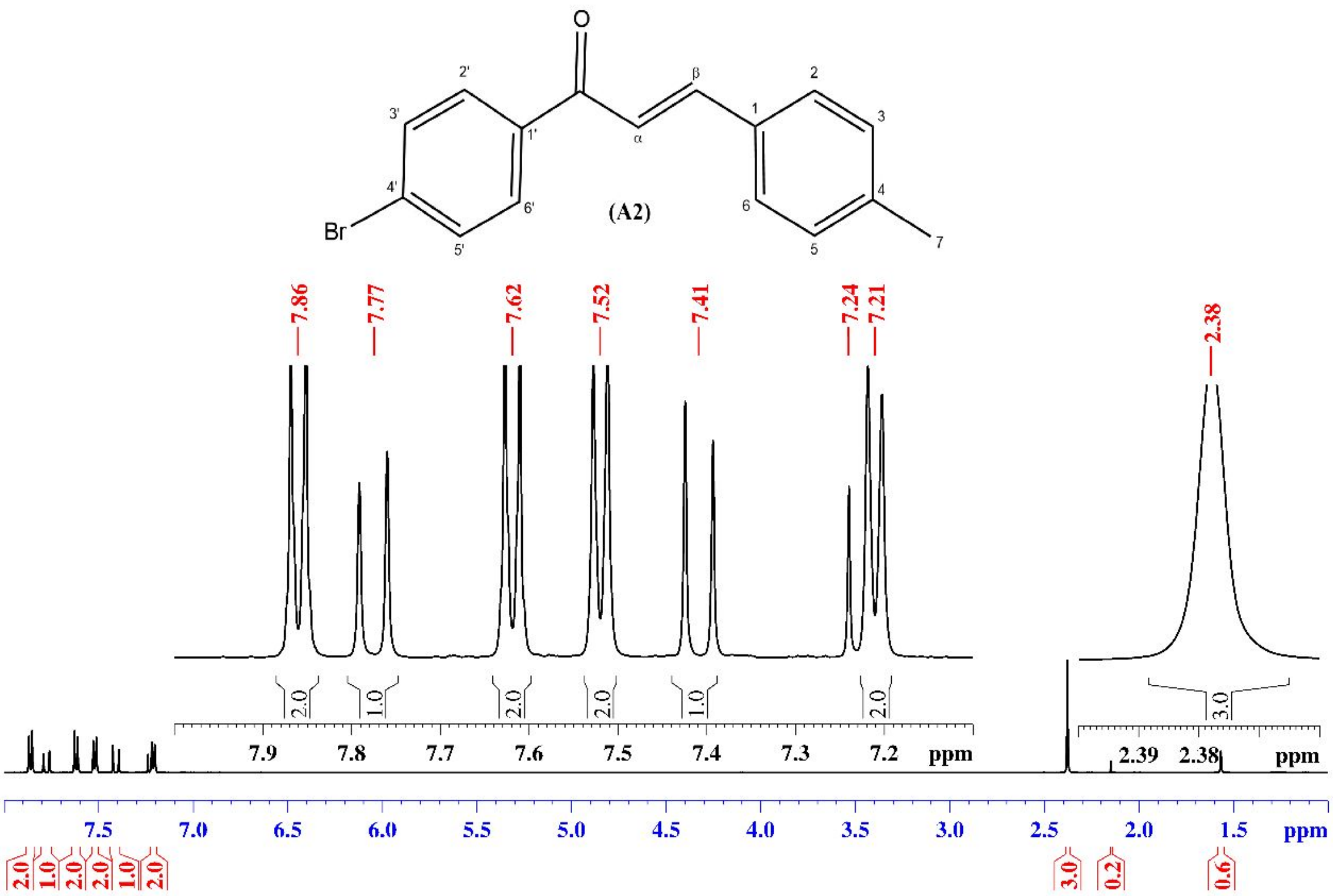

Figure S4. ^{1}H NMR spectra, in CDCl3 (7.24 ppm), of **A2**.:

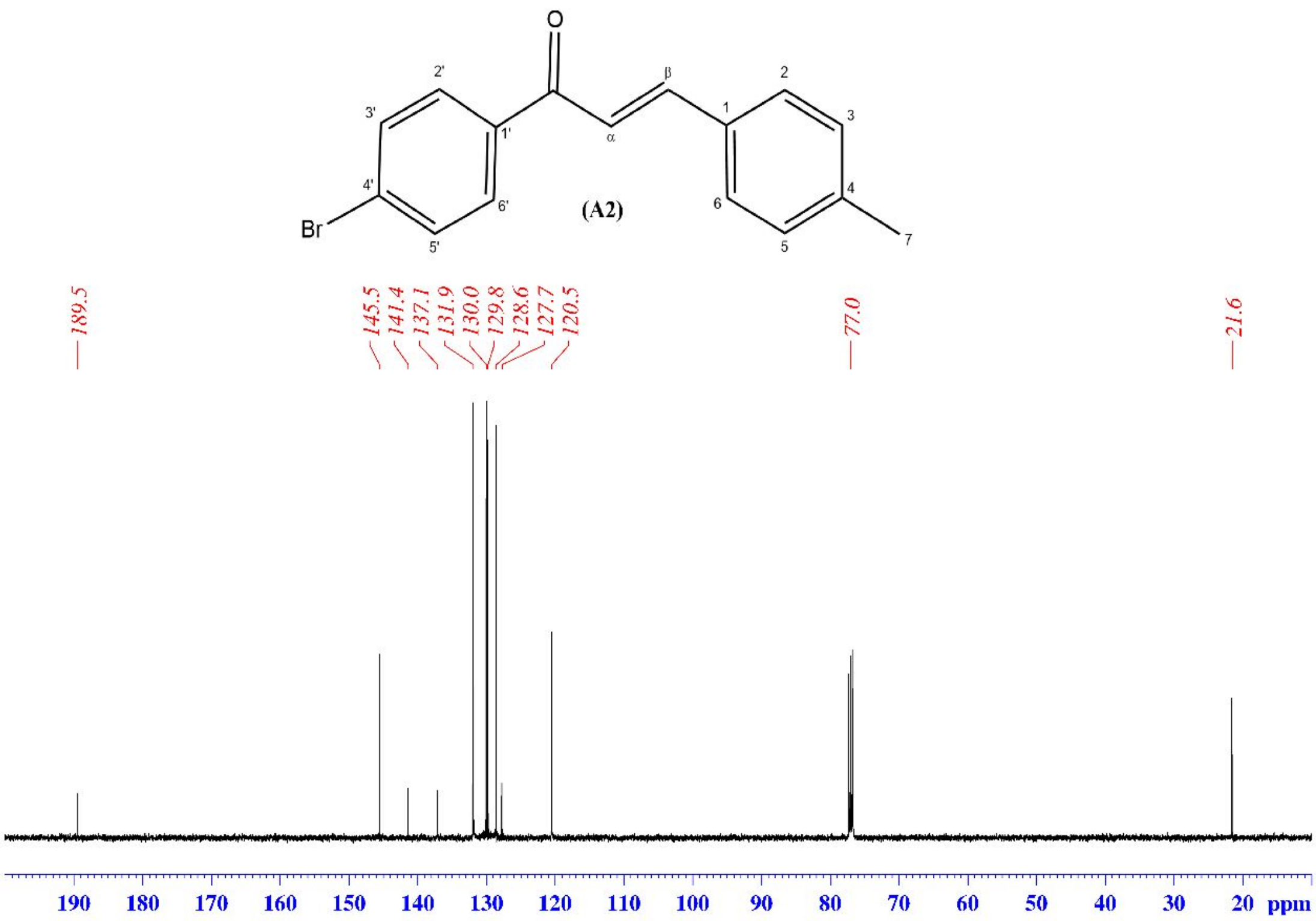

Figure S5. ^{13}C NMR spectra, in CDCl3 (77.2 ppm), of **A2**

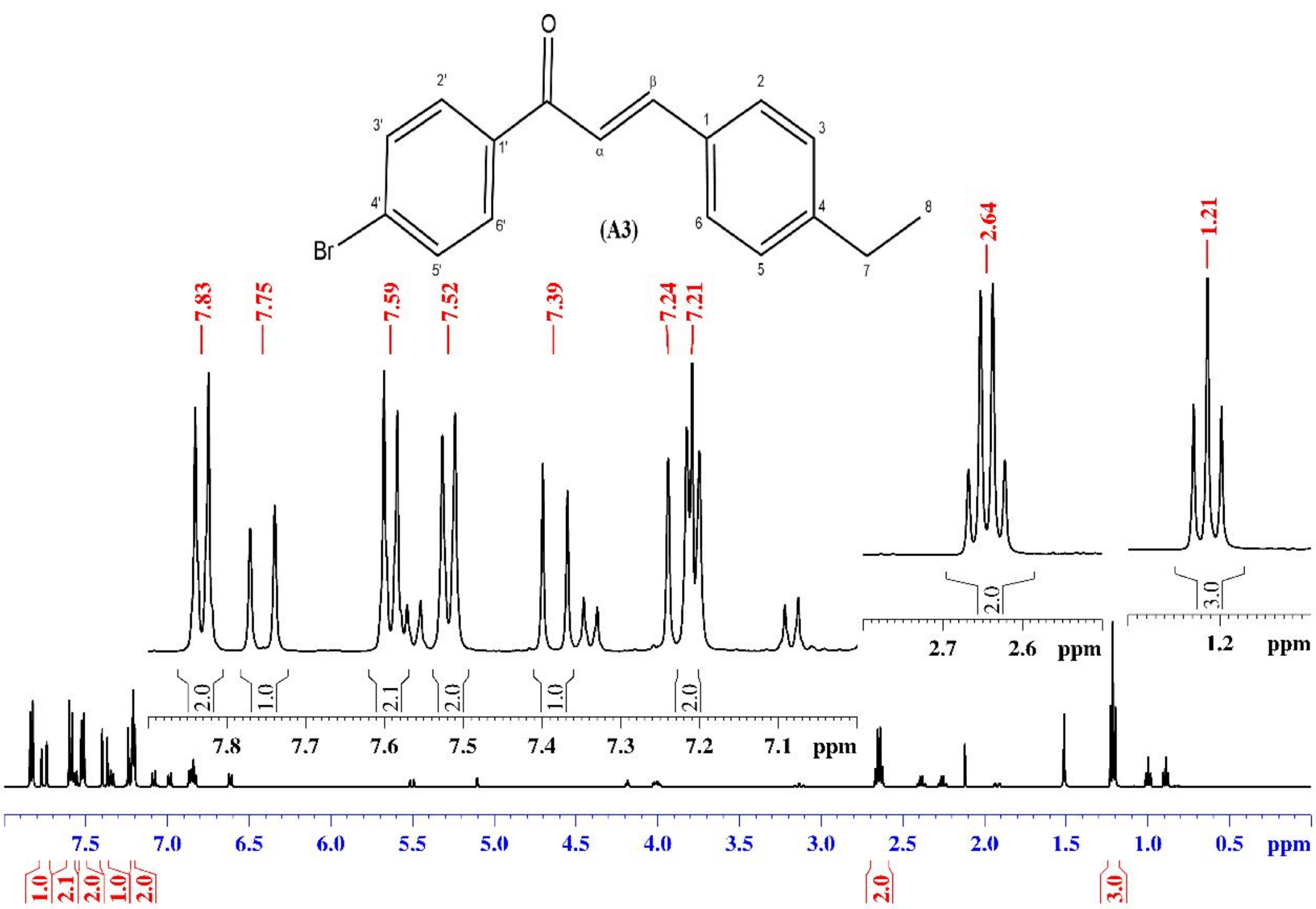

Figure S6. ^{1}H NMR spectra, in CDCl3 (7.24 ppm), of **A3**.:

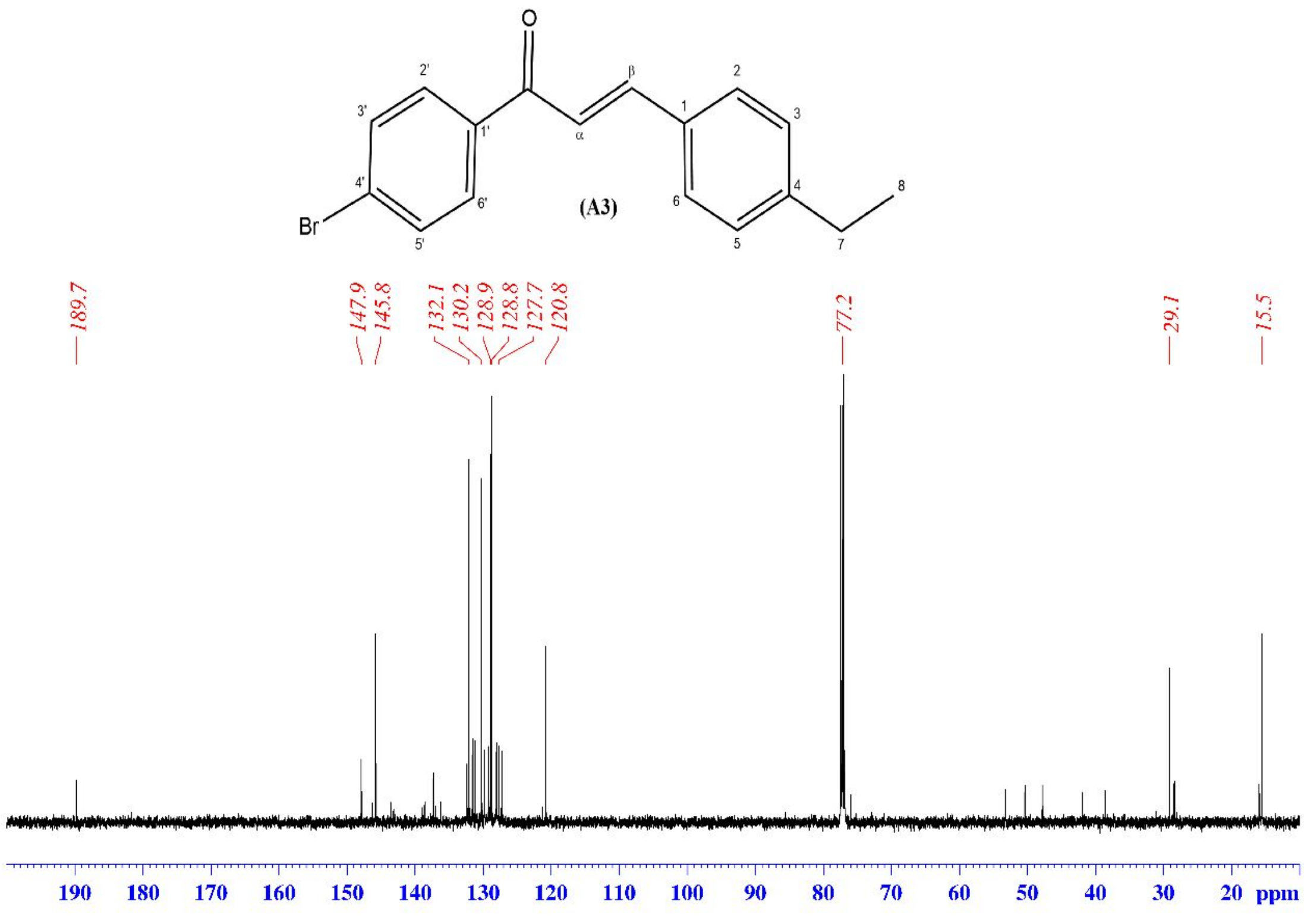

Figure S7. ^{13}C NMR spectra, in CDCl3 (77.2 ppm), of **A3**

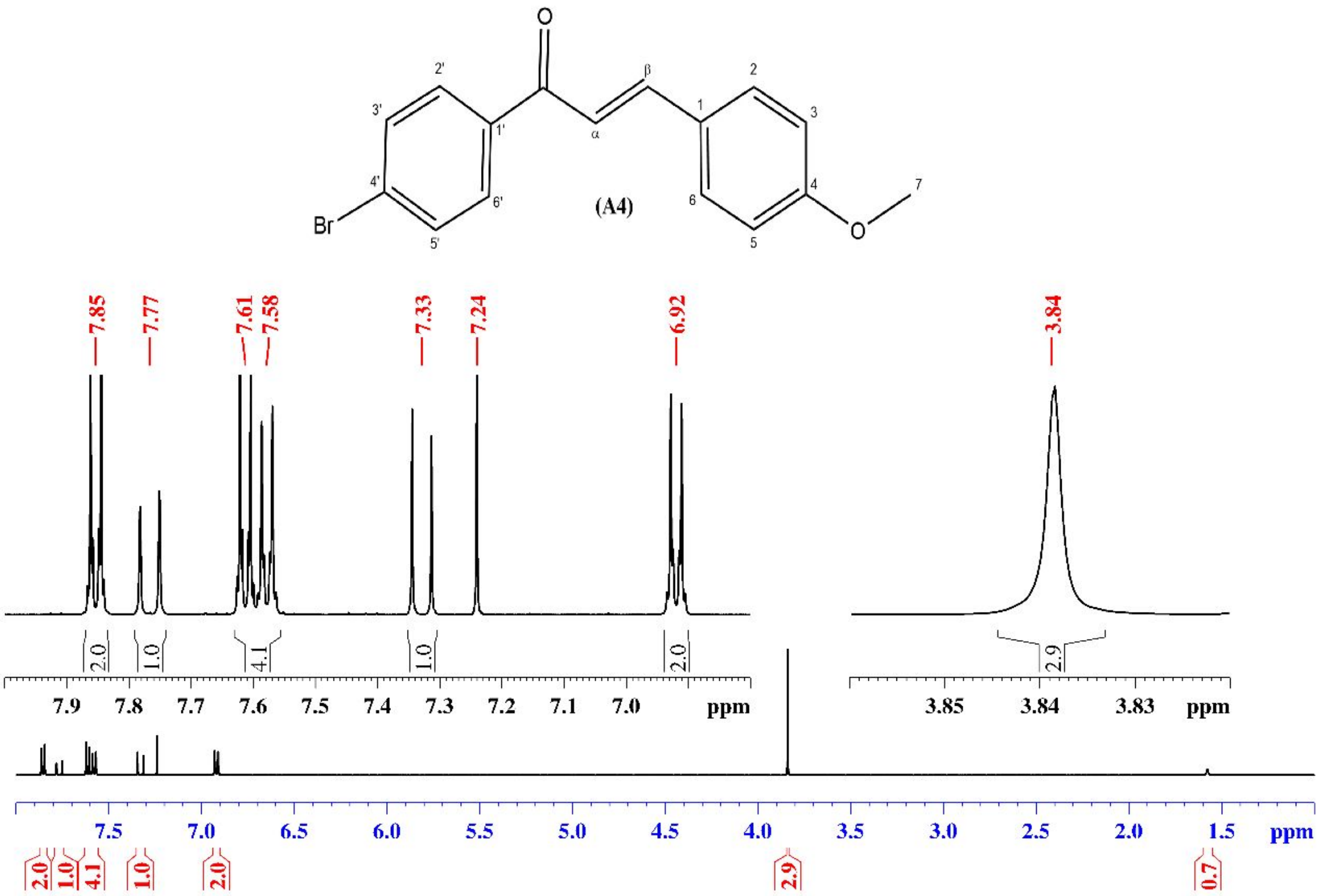

Figure S8. ^{1}H NMR spectra, in CDCl3 (7.24 ppm), of **A4**.:

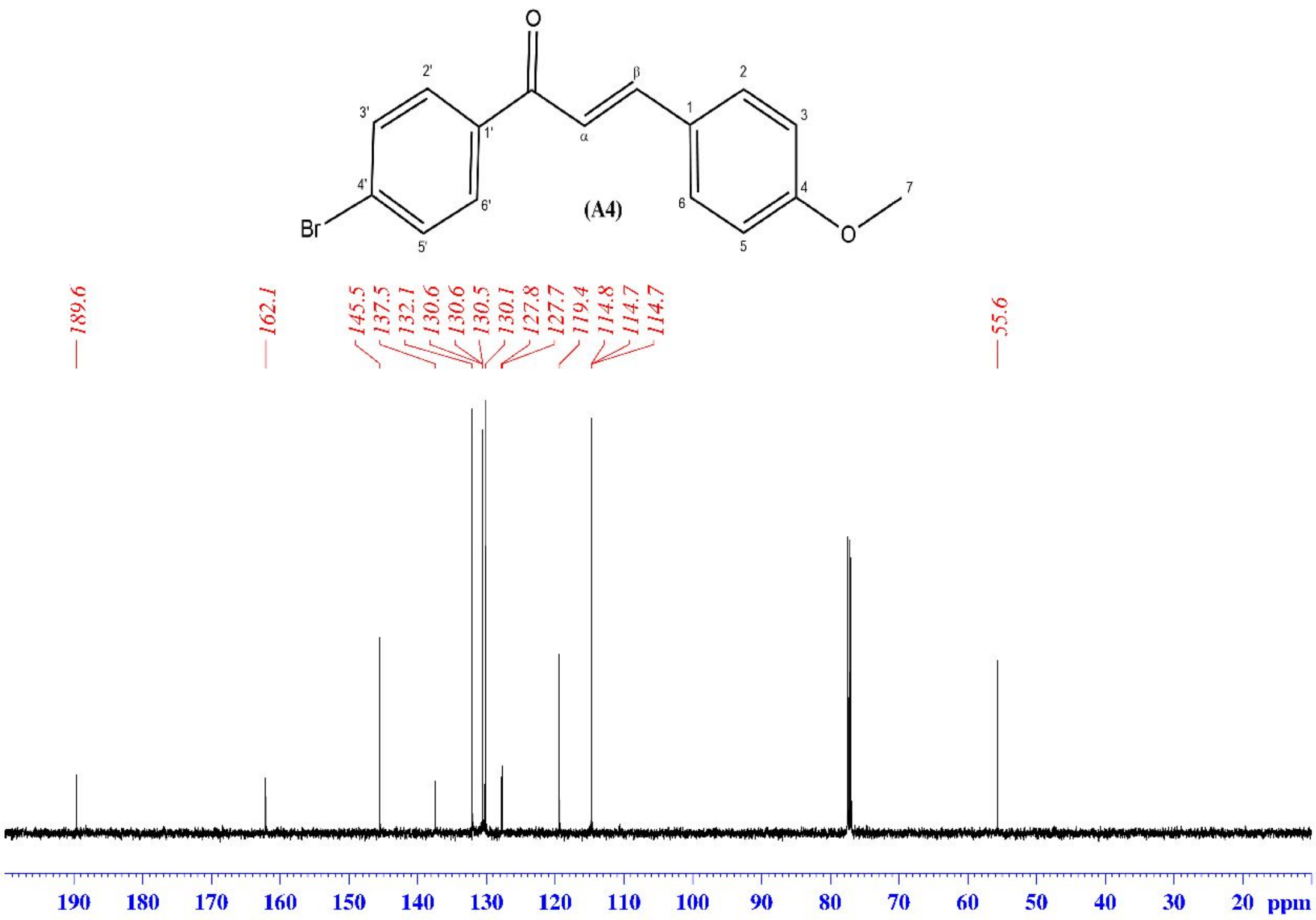

Figure S9. ^{13}C NMR spectra, in CDCl3 (77.2 ppm), of **A4**

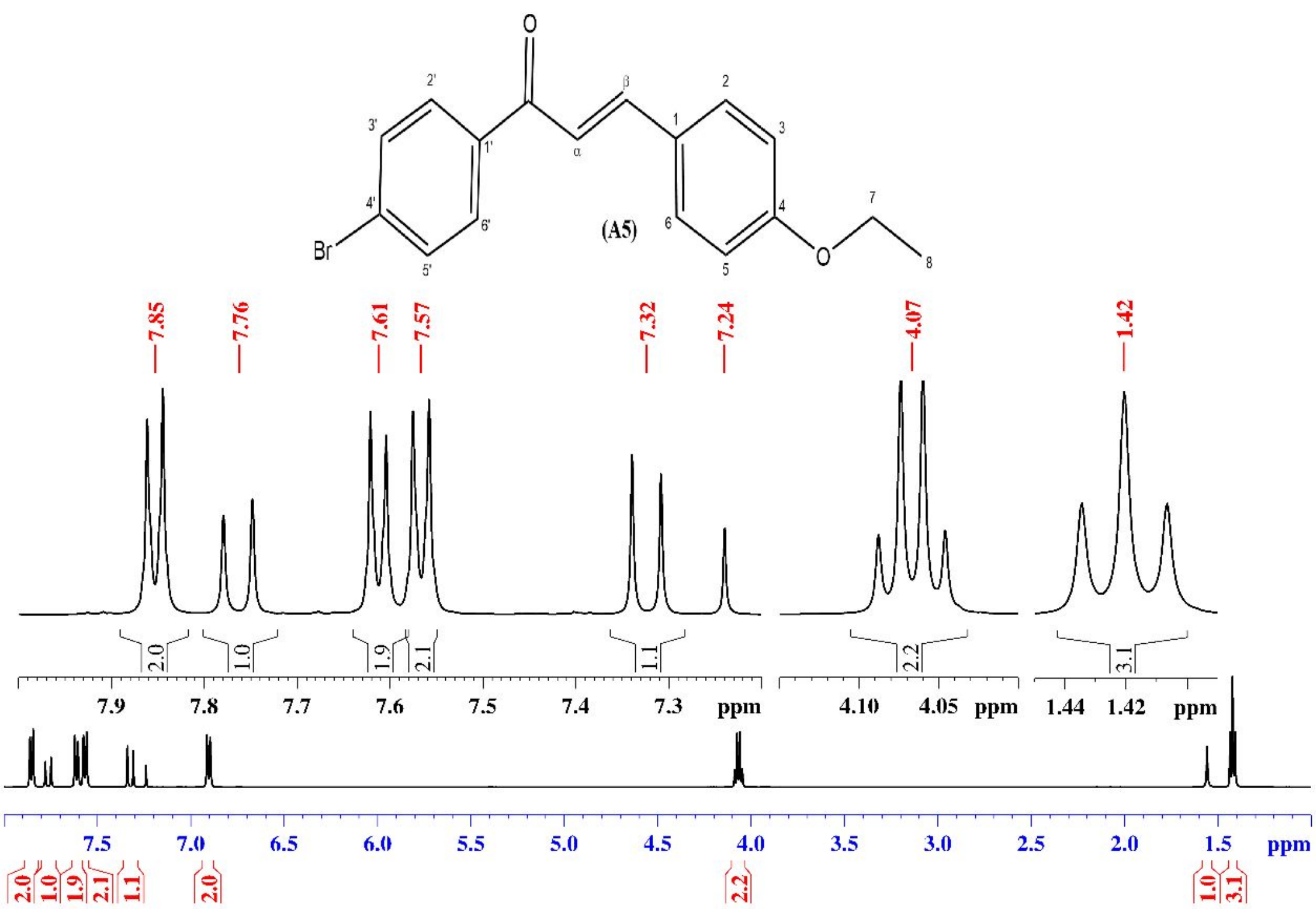

Figure S10. ^{1}H NMR spectra, in CDCl3 (7.24 ppm), of **A5**.:

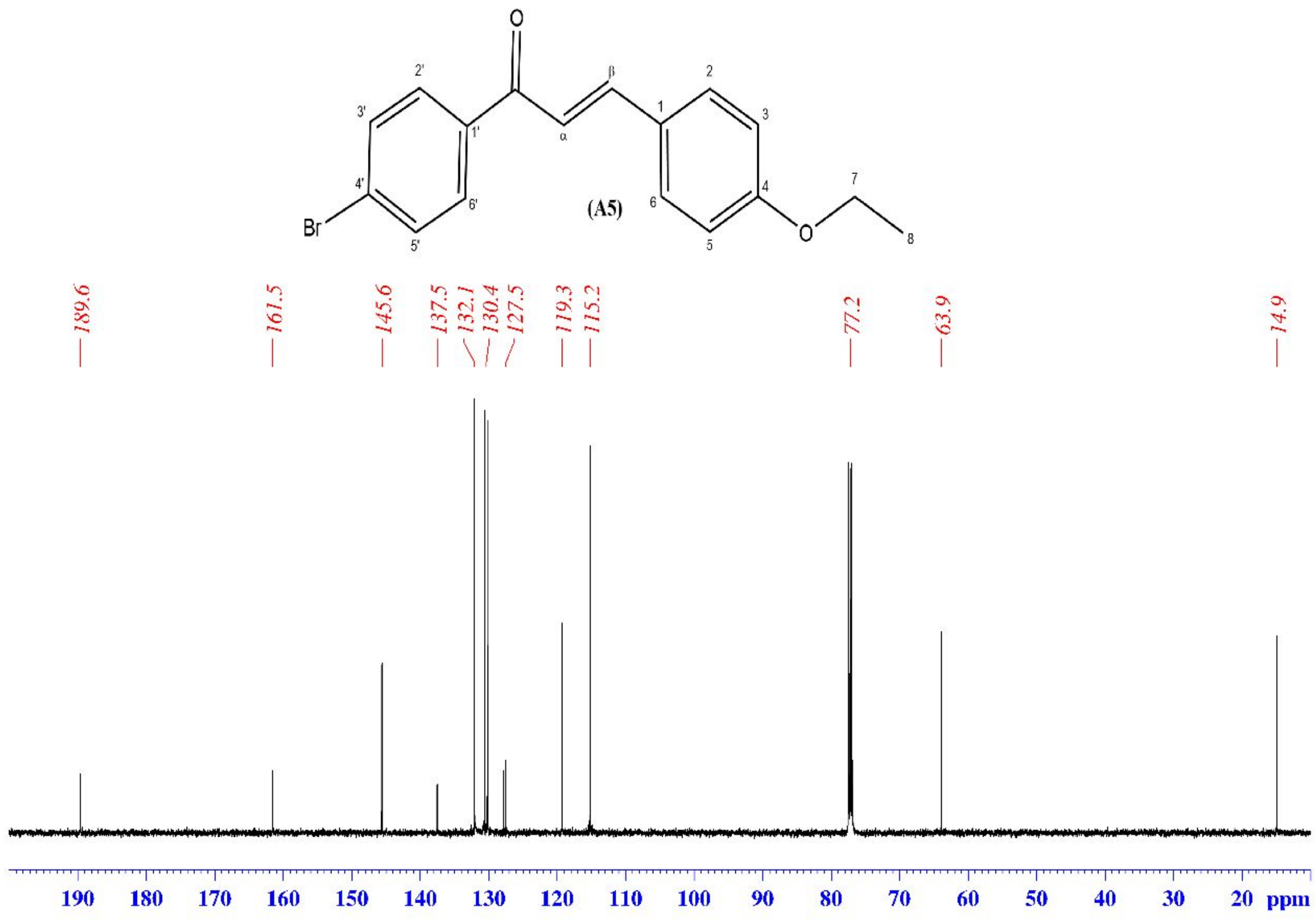

Figure S11. ^{13}C NMR spectra, in CDCl3 (77.2 ppm), of **A5.**

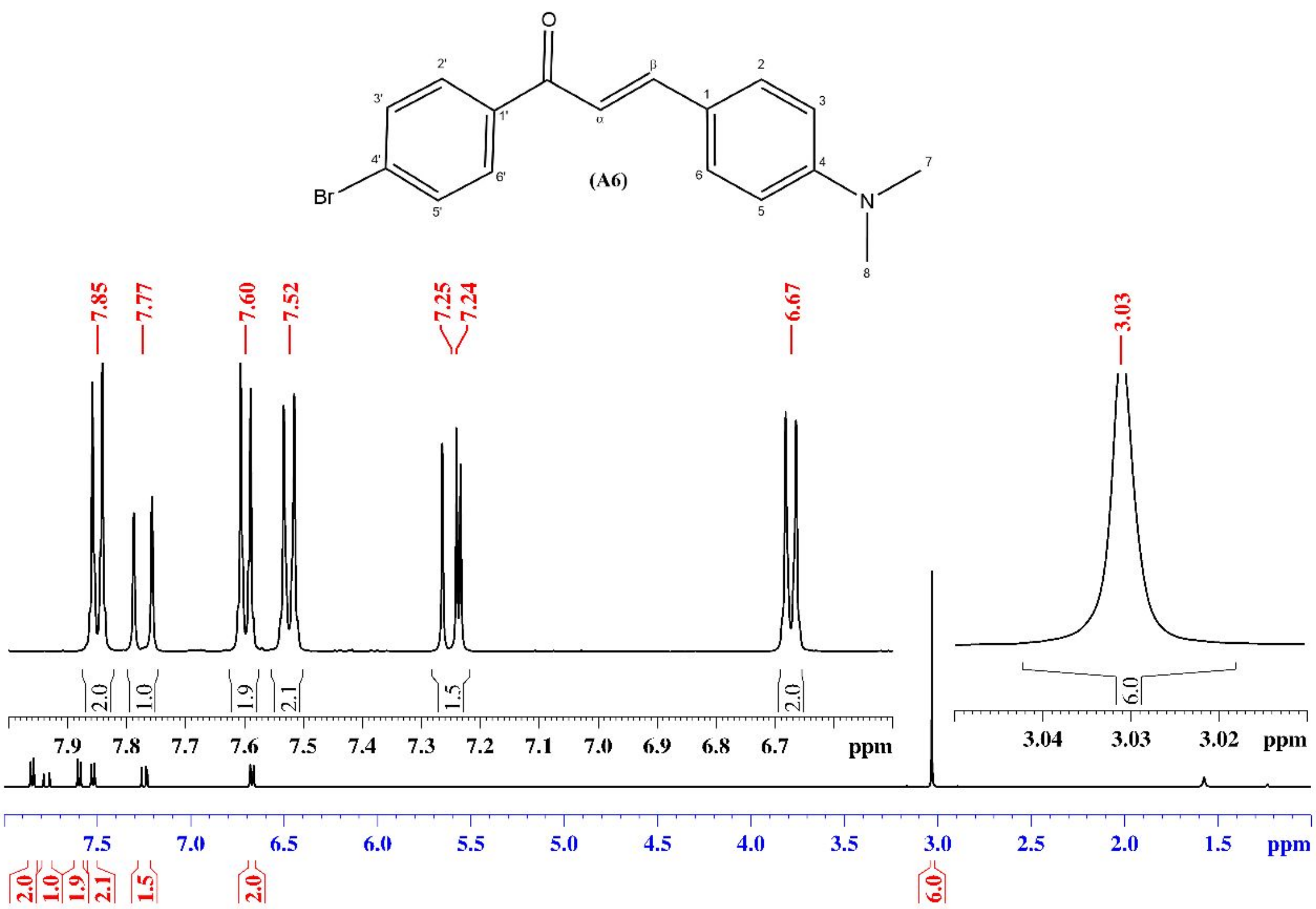

Figure S12. ^{1}H NMR spectra, in CDCl3 (7.24 ppm), of **A6**.:

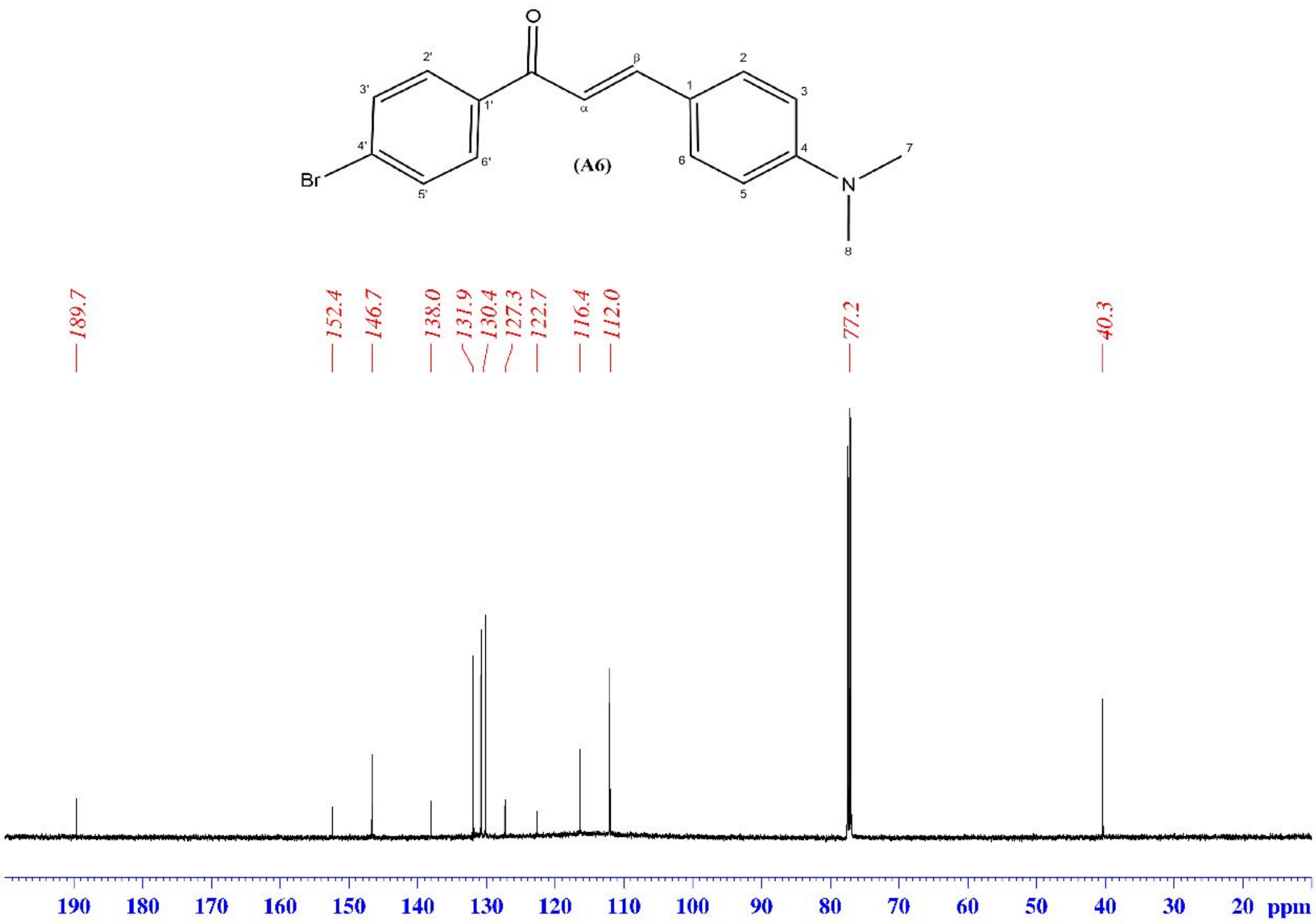

Figure S13. ^{13}C NMR spectra, in CDCl3 (77.2 ppm), of **A6**

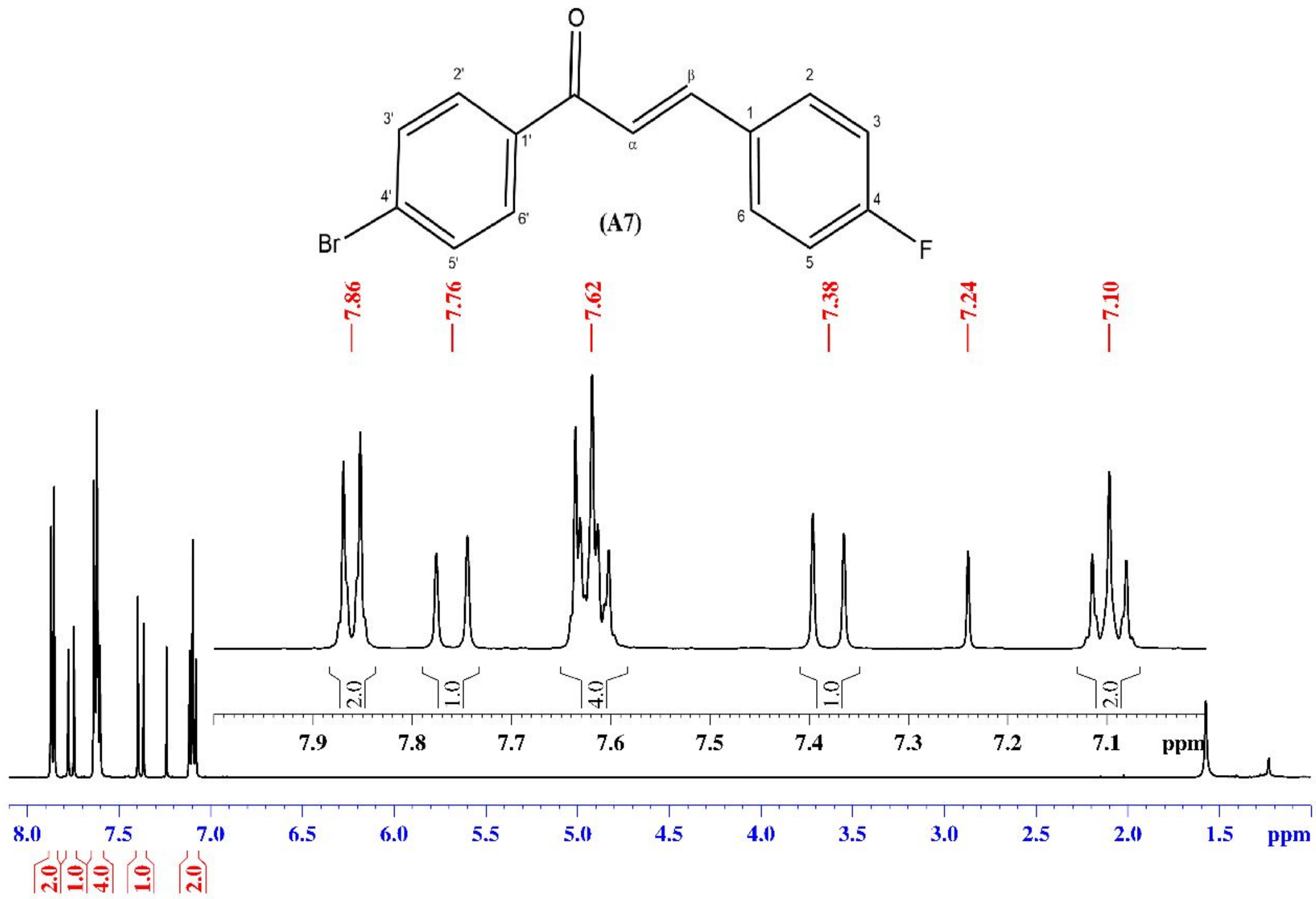

Figure S14. ^{1}H NMR spectra, in CDCl3 (7.24 ppm), of **A7**.:

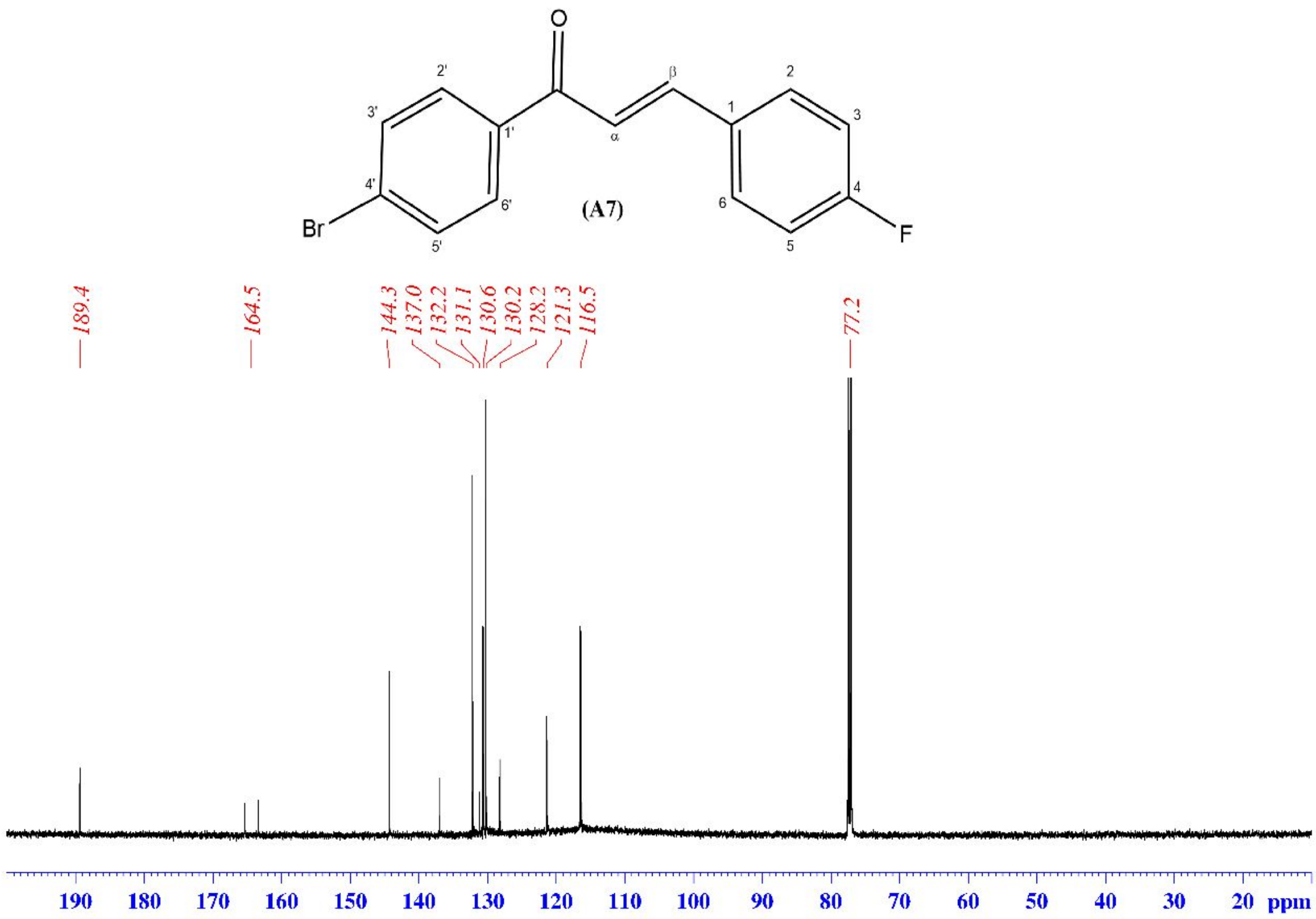

Figure S15. ^{13}C NMR spectra, in CDCl3 (77.2 ppm), of **A7**

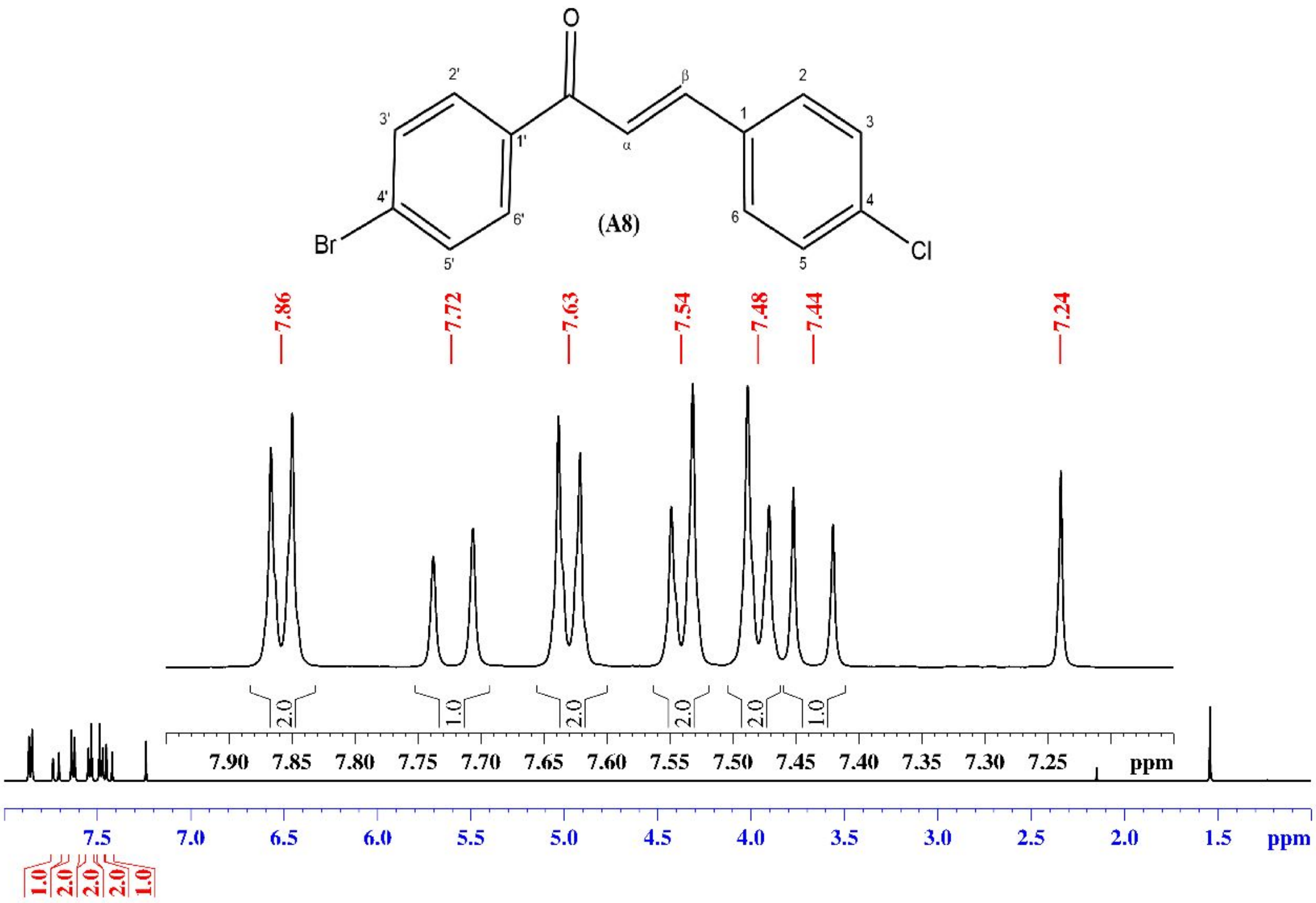

Figure S16. ^{1}H NMR spectra, in CDCl3 (7.24 ppm), of **A8**.:

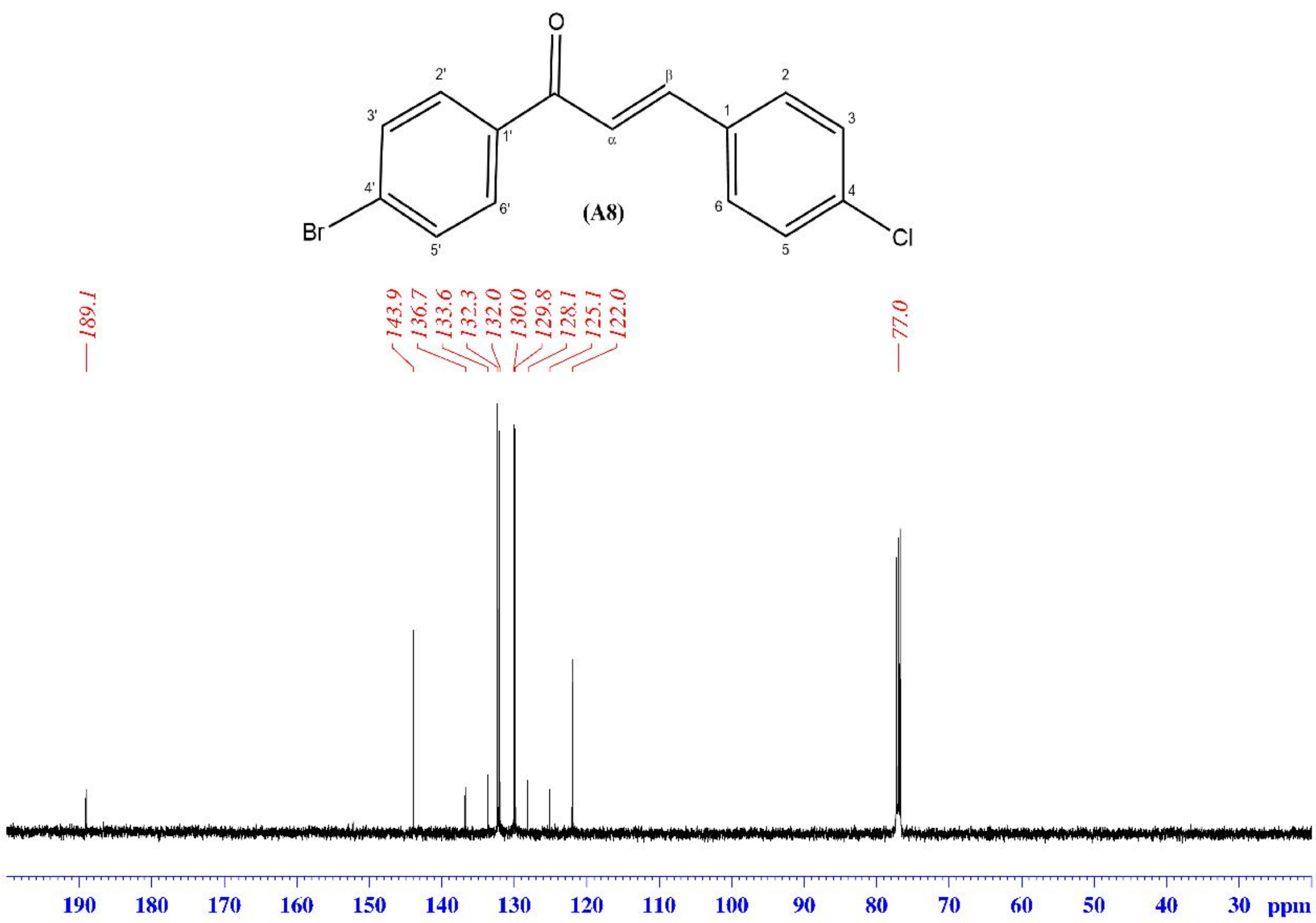

Figure S17. ^{13}C NMR spectra, in CDCl3 (77.2 ppm), of **A8**

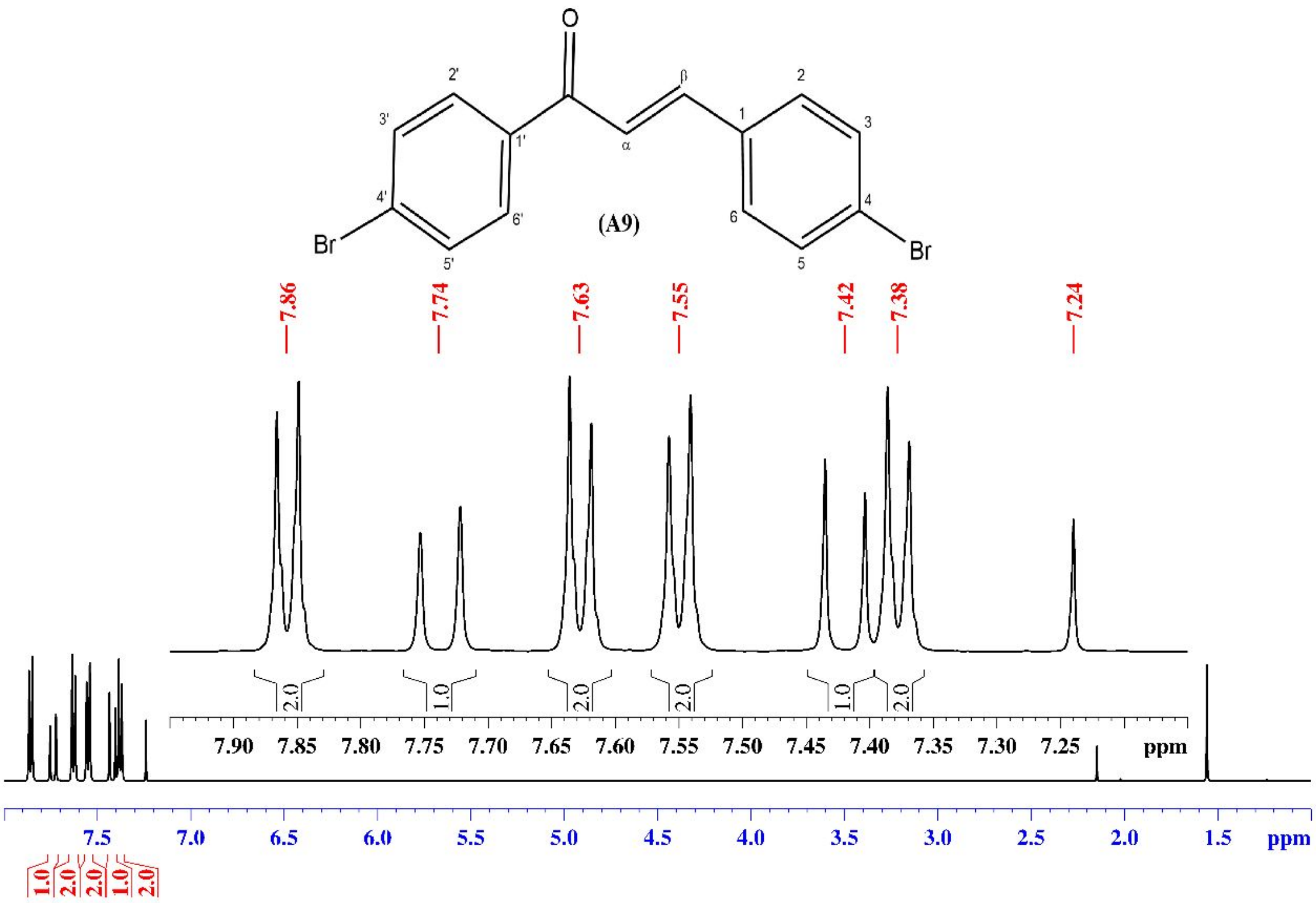

Figure S18. ¹H NMR spectra, in CDCl3 (7.24 ppm), of **A9**.:

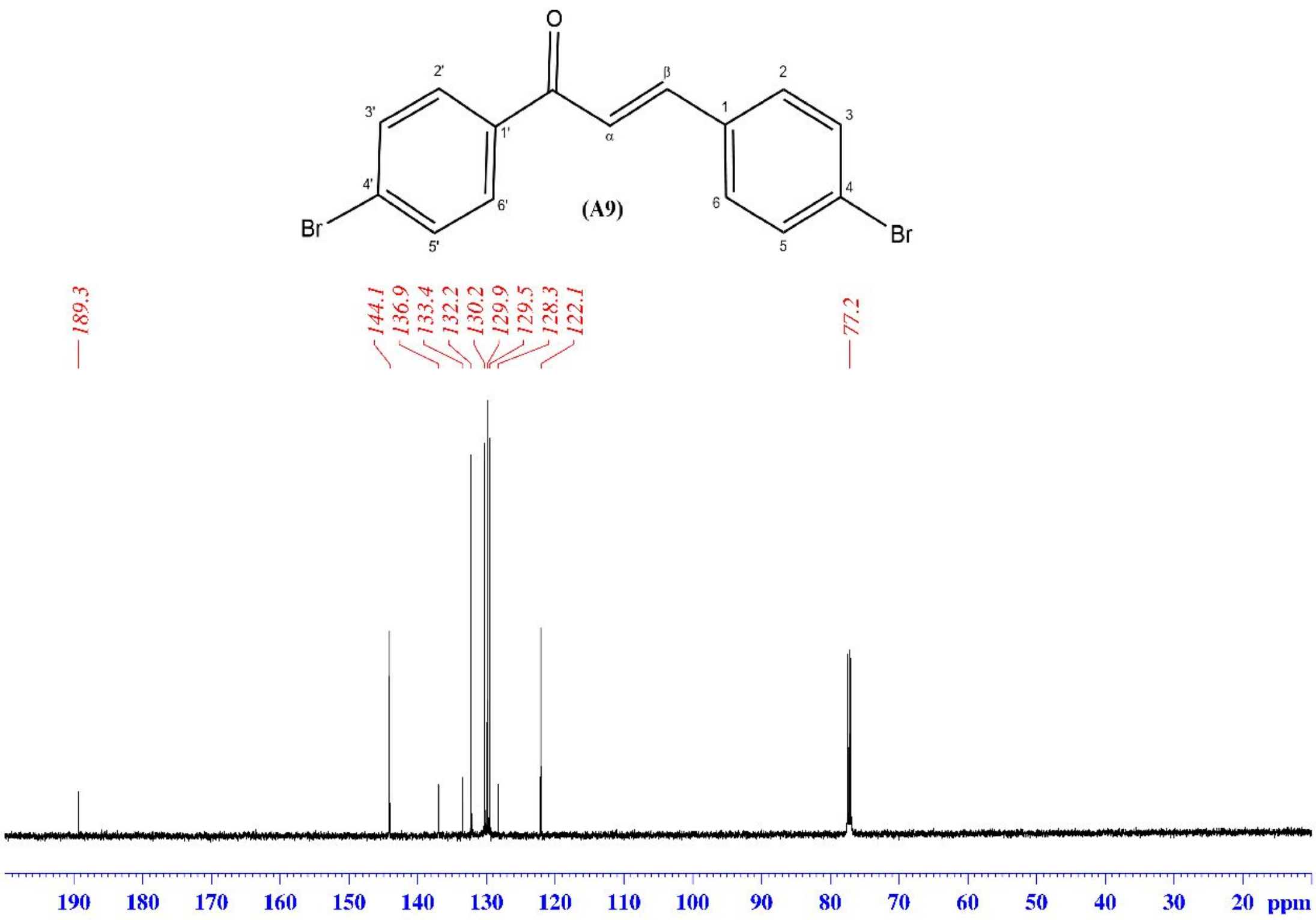

Figure S19. ^{13}C NMR spectra, in CDCl3 (77.2 ppm), of **A9**

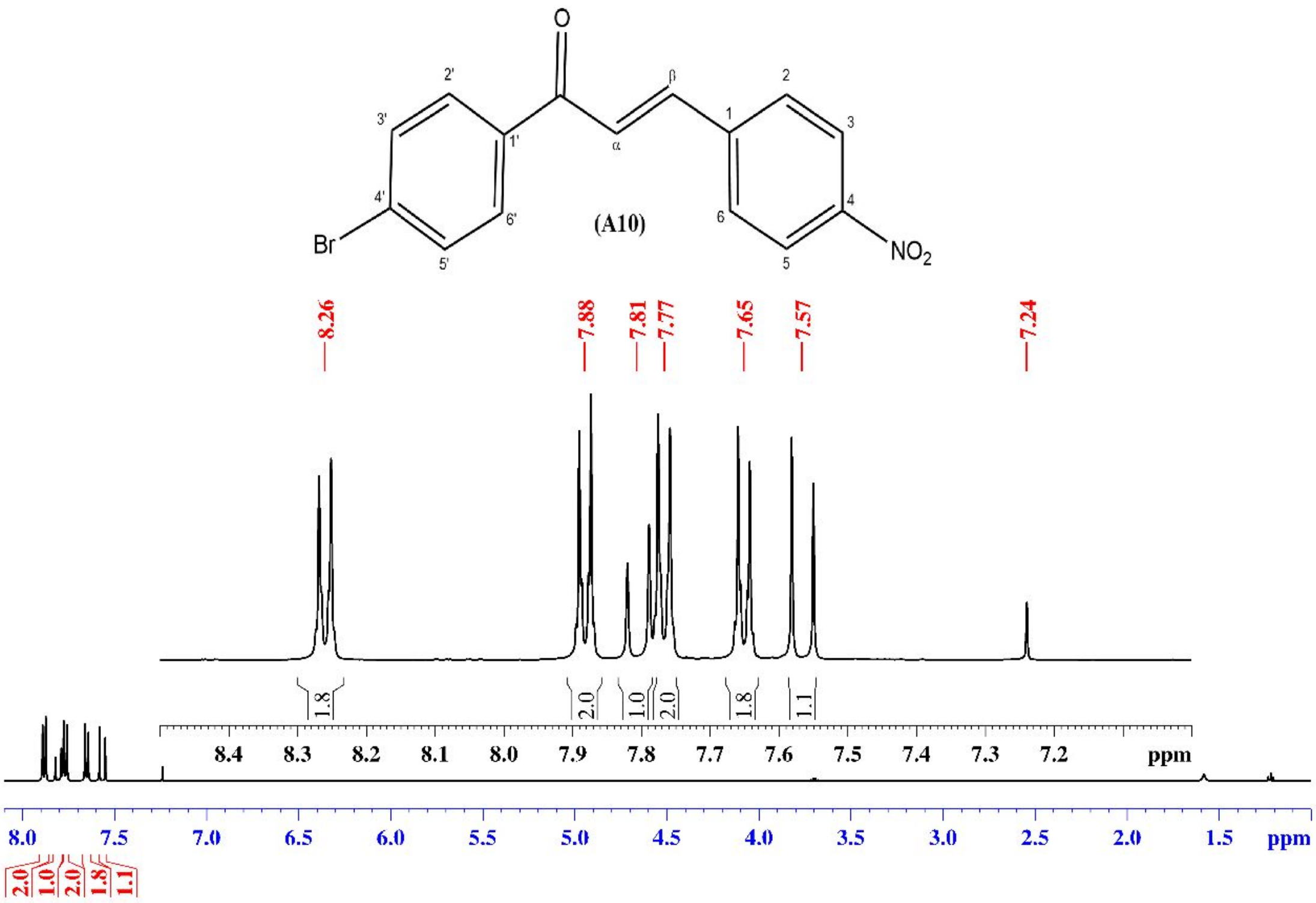

Figure S20. ^{1}H NMR spectra, in CDCl3 (7.24 ppm), of **A10.**:

- **1.4 HRMS fragmentation spectra**

The structural characterization of chalcone derivatives was conducted through HRMS, employing CID fragmentation pathways to elucidate diagnostic ion signatures critical for unambiguous analyte identification. For MS/MS analyses, performed in positive ion mode with optimized collision energies (20-30 eV), revealed systematic cleavage patterns correlating with the chalcone core's electronic configuration and substituent topology. The base structure, comprising an α,β-unsaturated aromatic ketone scaffold (C6-C3-C6), exhibited a conserved fragmentation pathway involving retro-Diels-Alder (RDA) cleavage, yielding a diagnostic 4-bromobenzaldehyde fragment (C_7H_4OBr), at *m/z* 182.9401, as corroborated by isotopic distribution analysis (Figure S2, S3, S4). This persistent fragment, arising from carbonyl (C=O) β-scission, confirms the integrity of the brominated A-ring across all analogs, serving as a structural fingerprint.

Substituent-dependent fragmentation mechanisms were observed, with chloride (-Cl), methoxy (-OCH3), ethoxy (-OC2H5), N-methanidylmethanamine (-C2H6N), fluorine (-F) and Bromide (-Br). This core structure is responsible for its antibacterial,

antifungal, and anti-inflammatory properties. Consequently, the incorporation of different functional groups broadens the scope of chalcone applications [2]

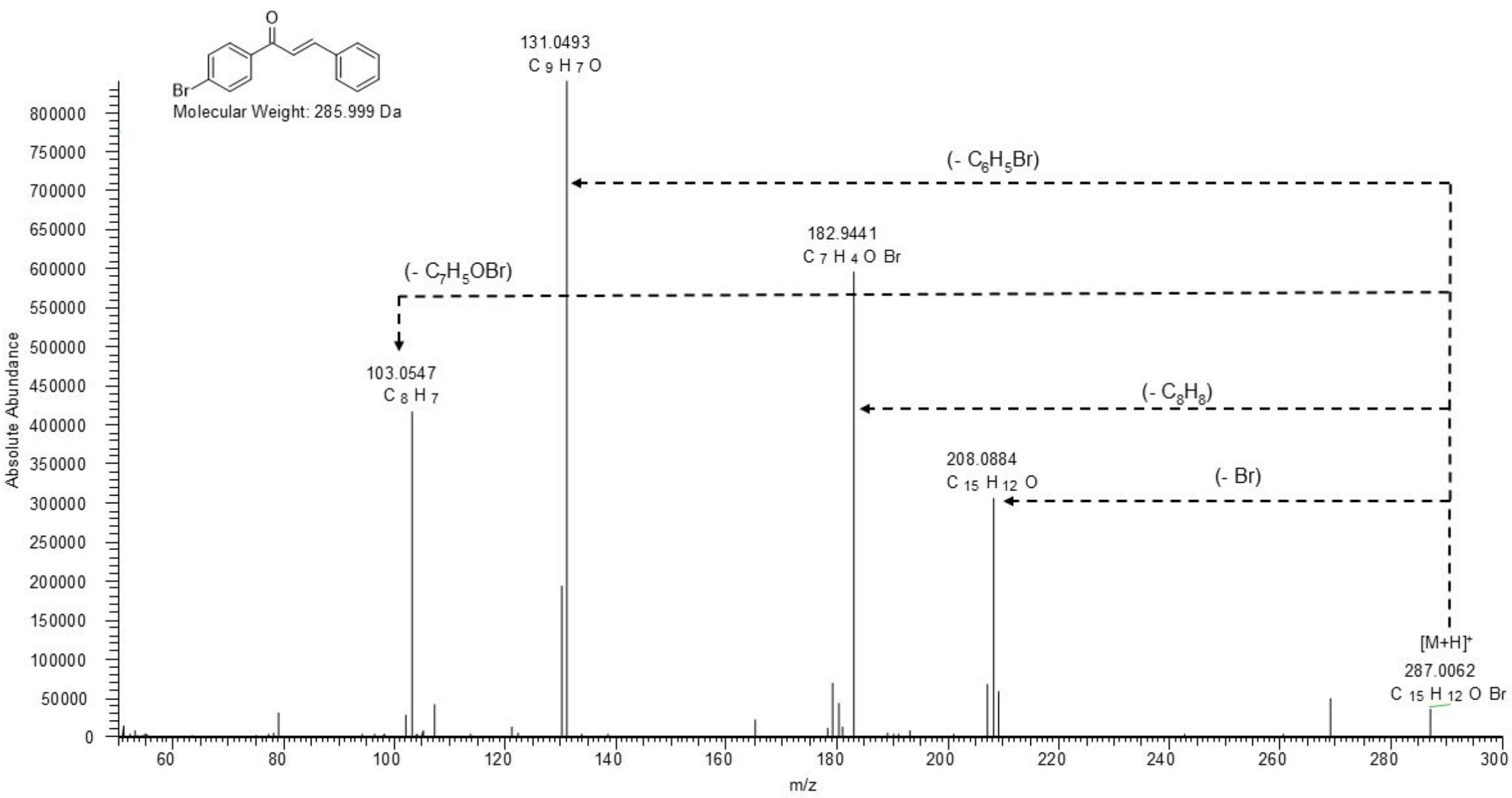

Figure S21. Fragmentation profile of sample **A1**.

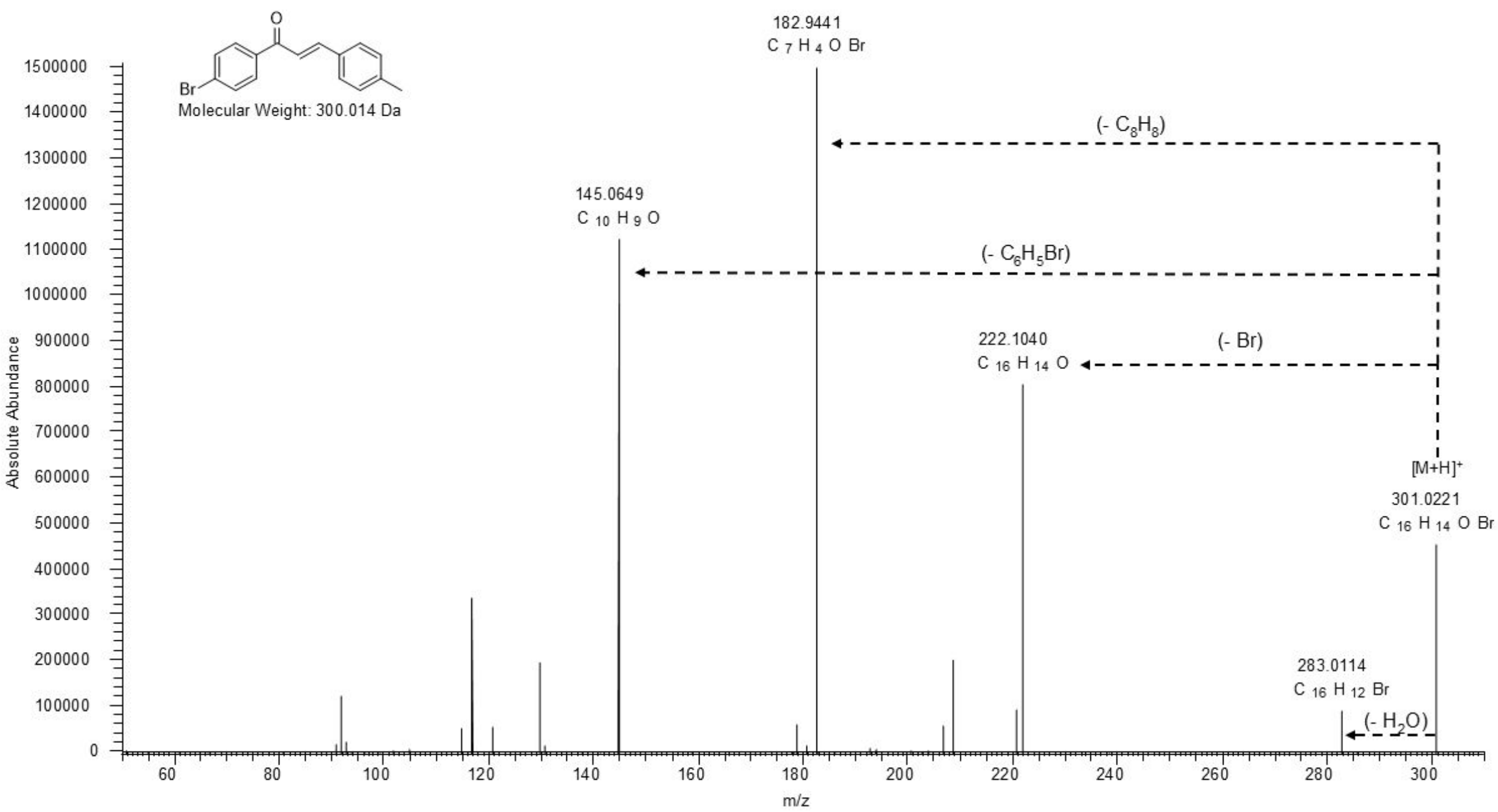

Figure S22. Fragmentation profile of sample **A2**.

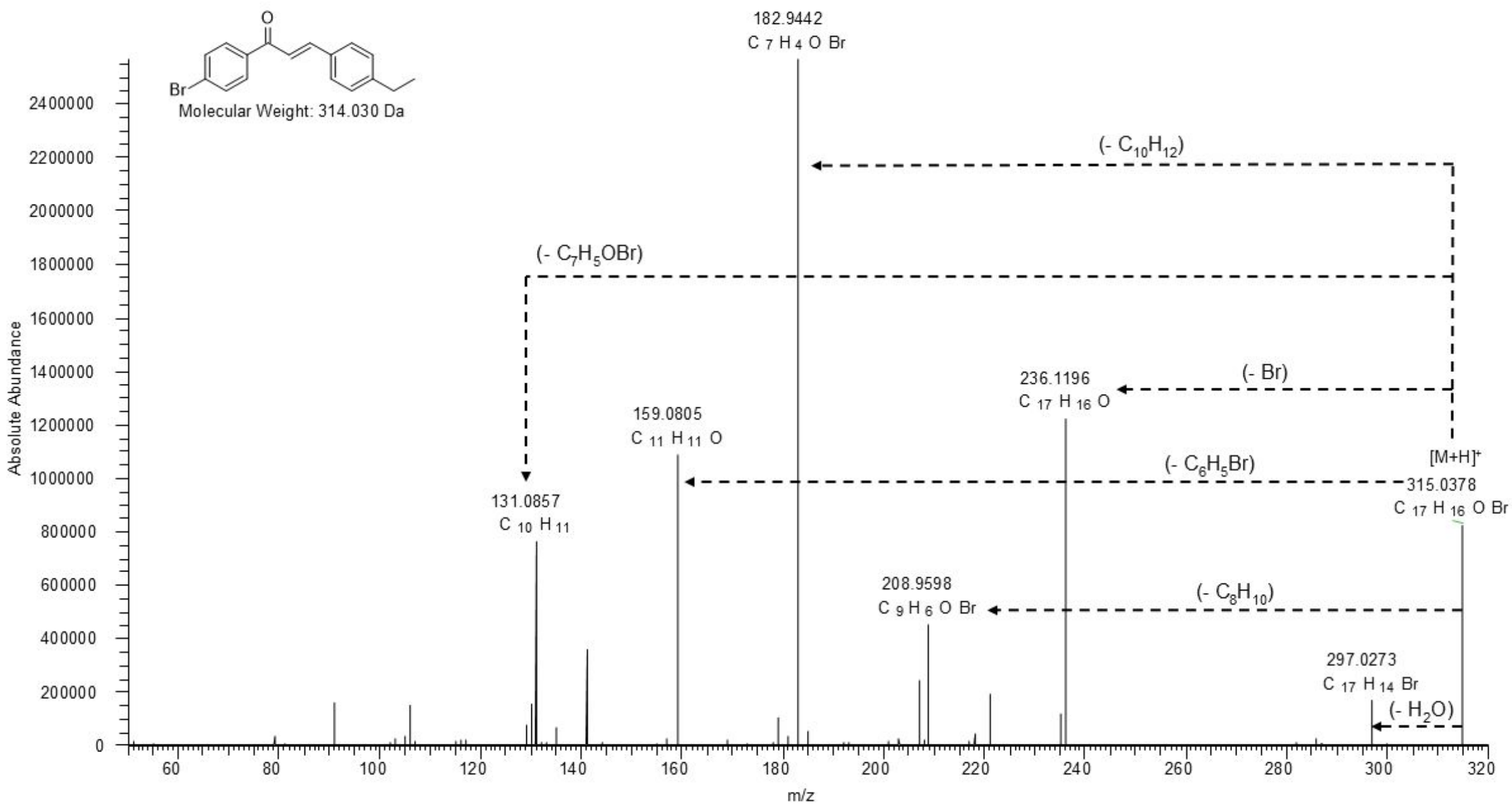

Figure S23. Fragmentation profile of sample **A3**.

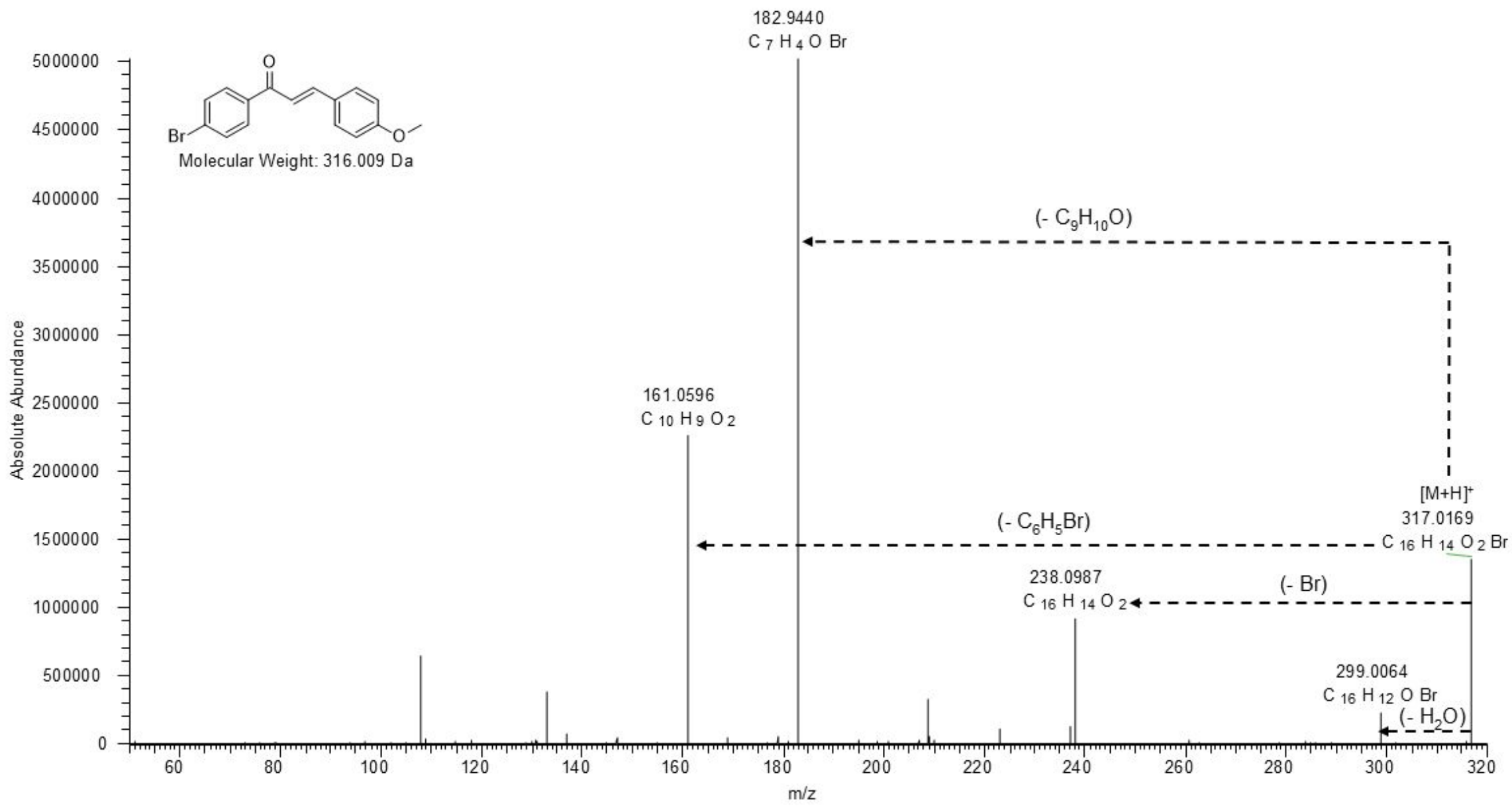

Figure S24. Fragmentation profile of sample **A4**.

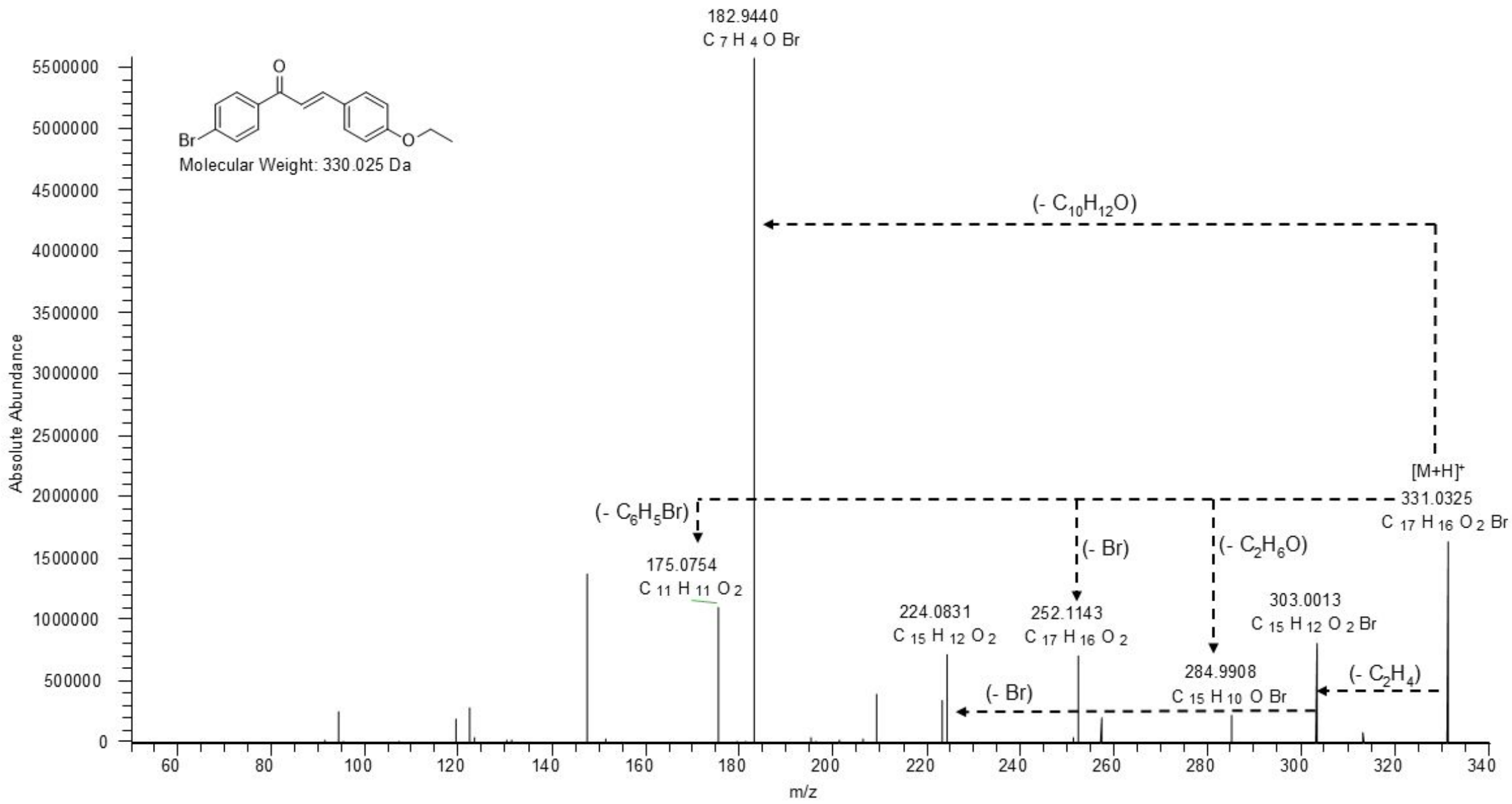

Figure S25. Fragmentation profile of sample **A5**.

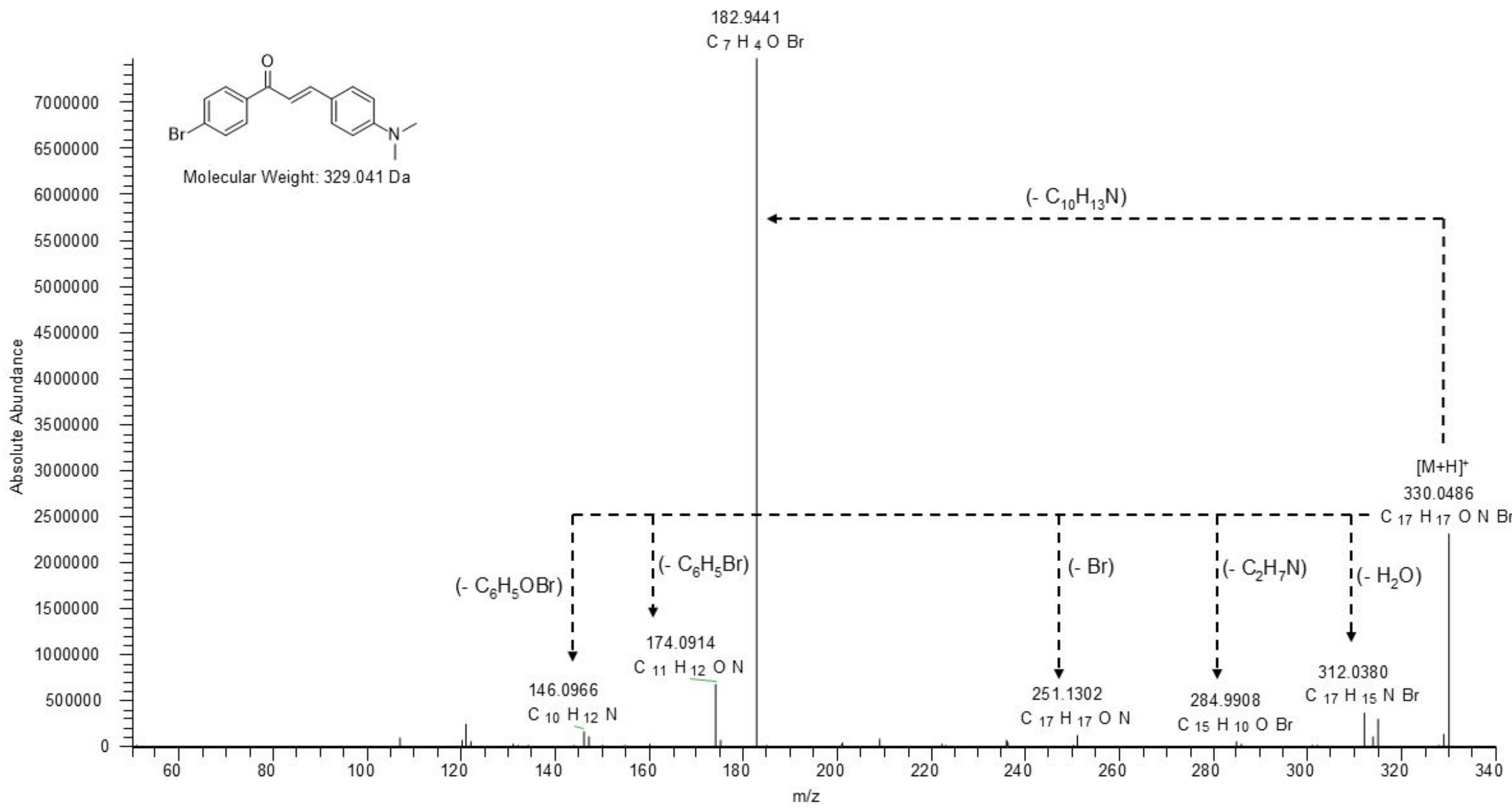

Figure S26. Fragmentation profile of sample **A6**.

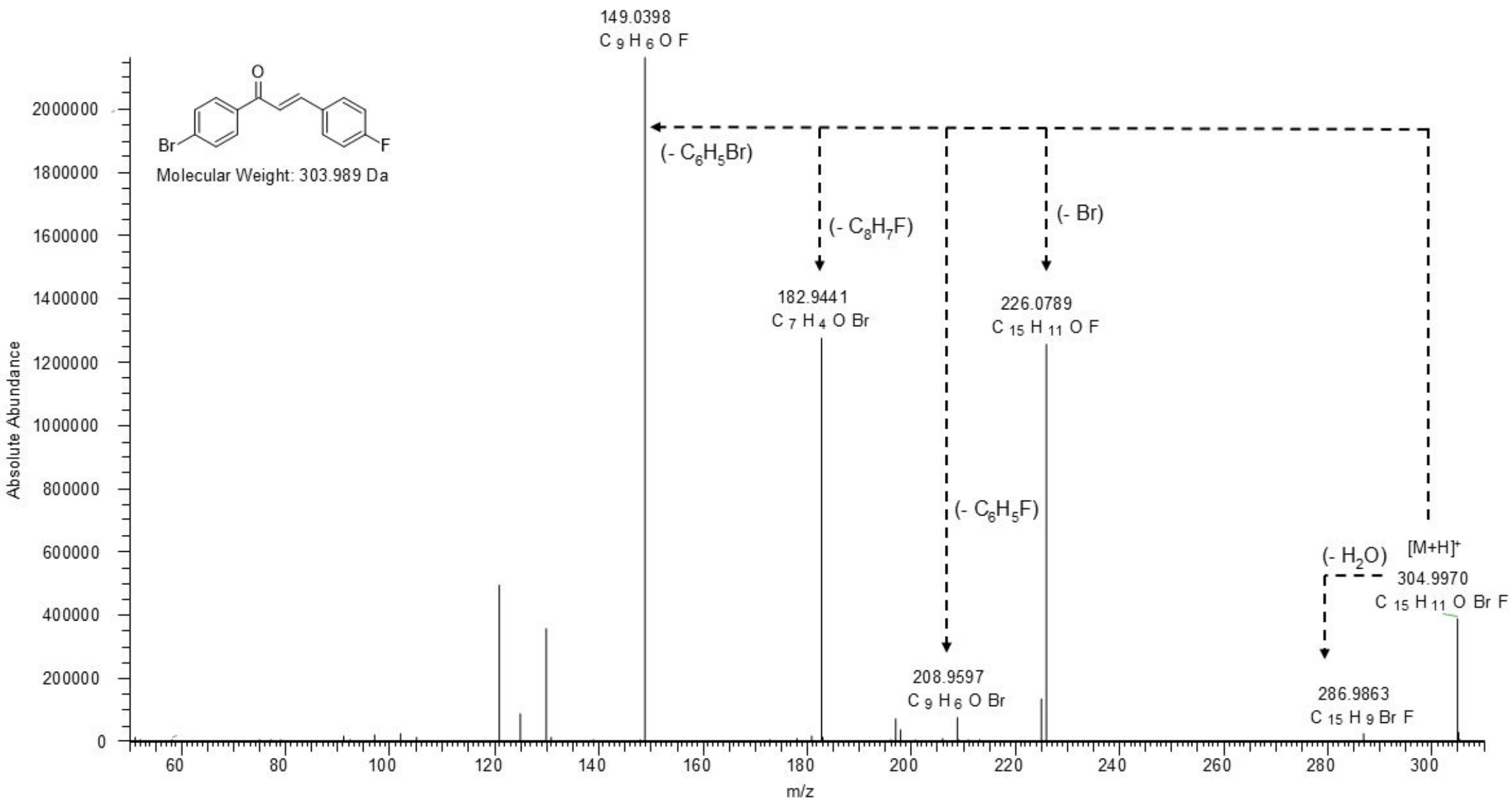

Figure S27. Fragmentation profile of sample **A7**.

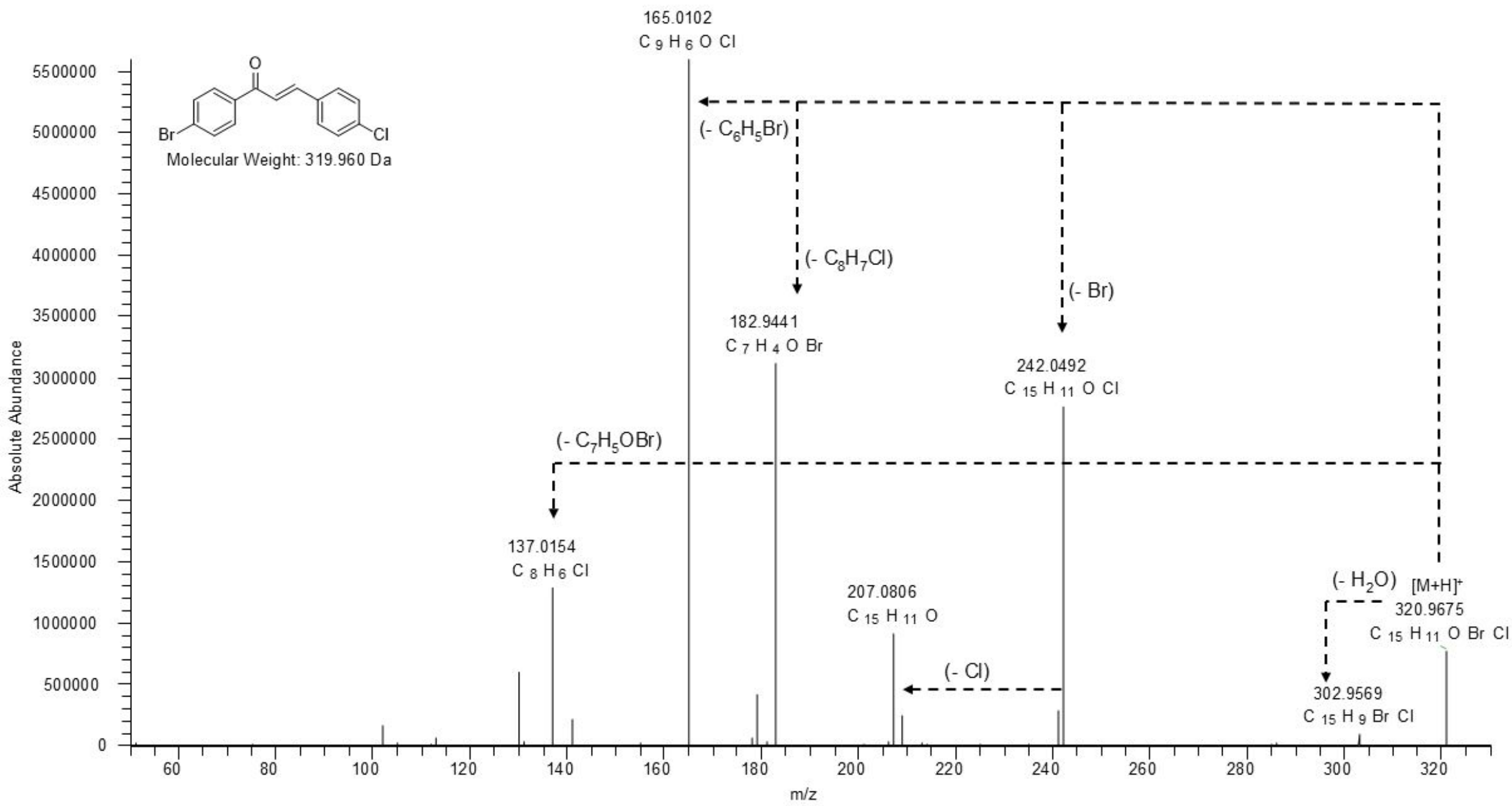

Figure S28. Fragmentation profile of sample **A8**.

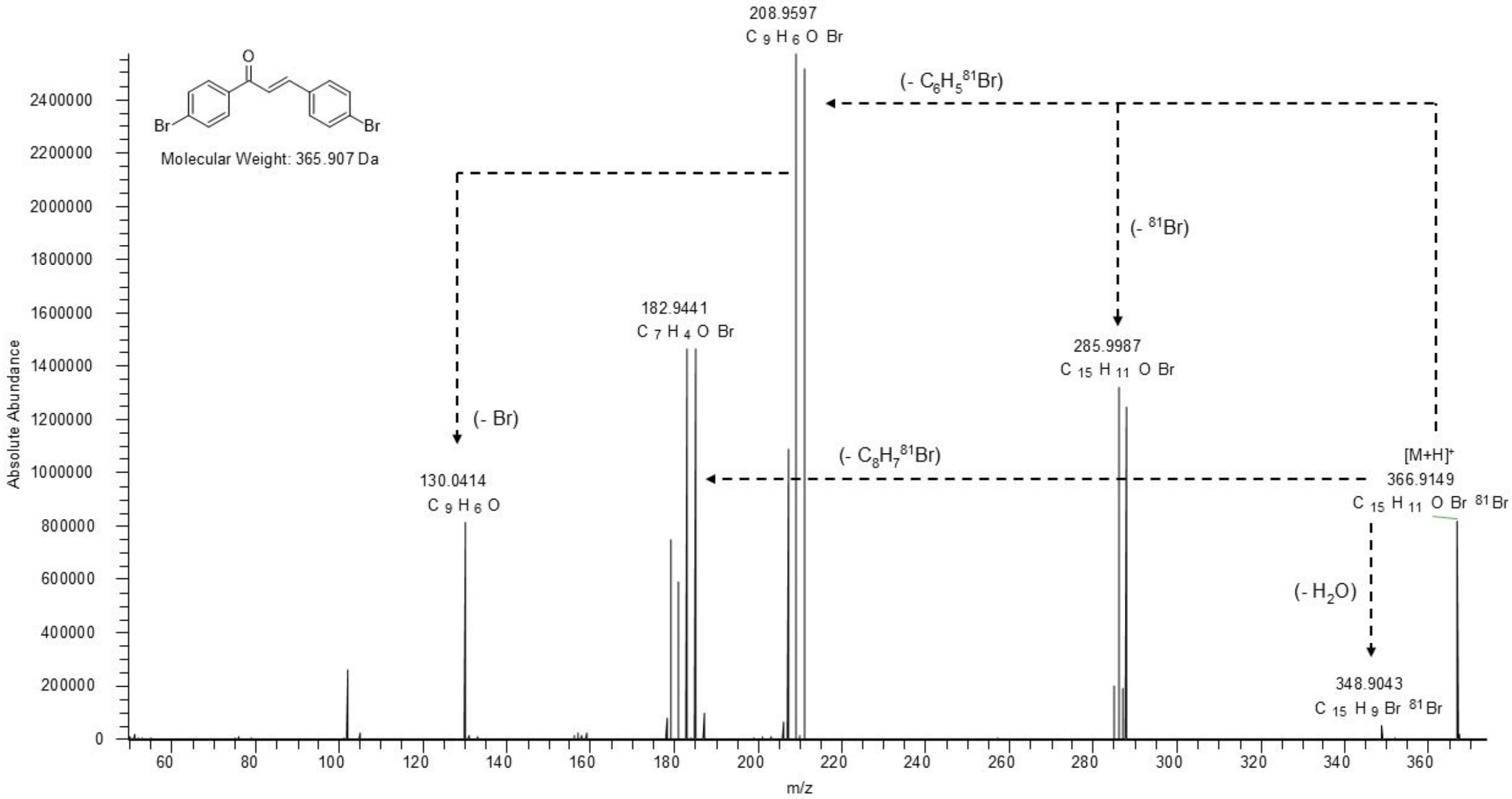

Figure S29. Fragmentation profile of sample **A9**.

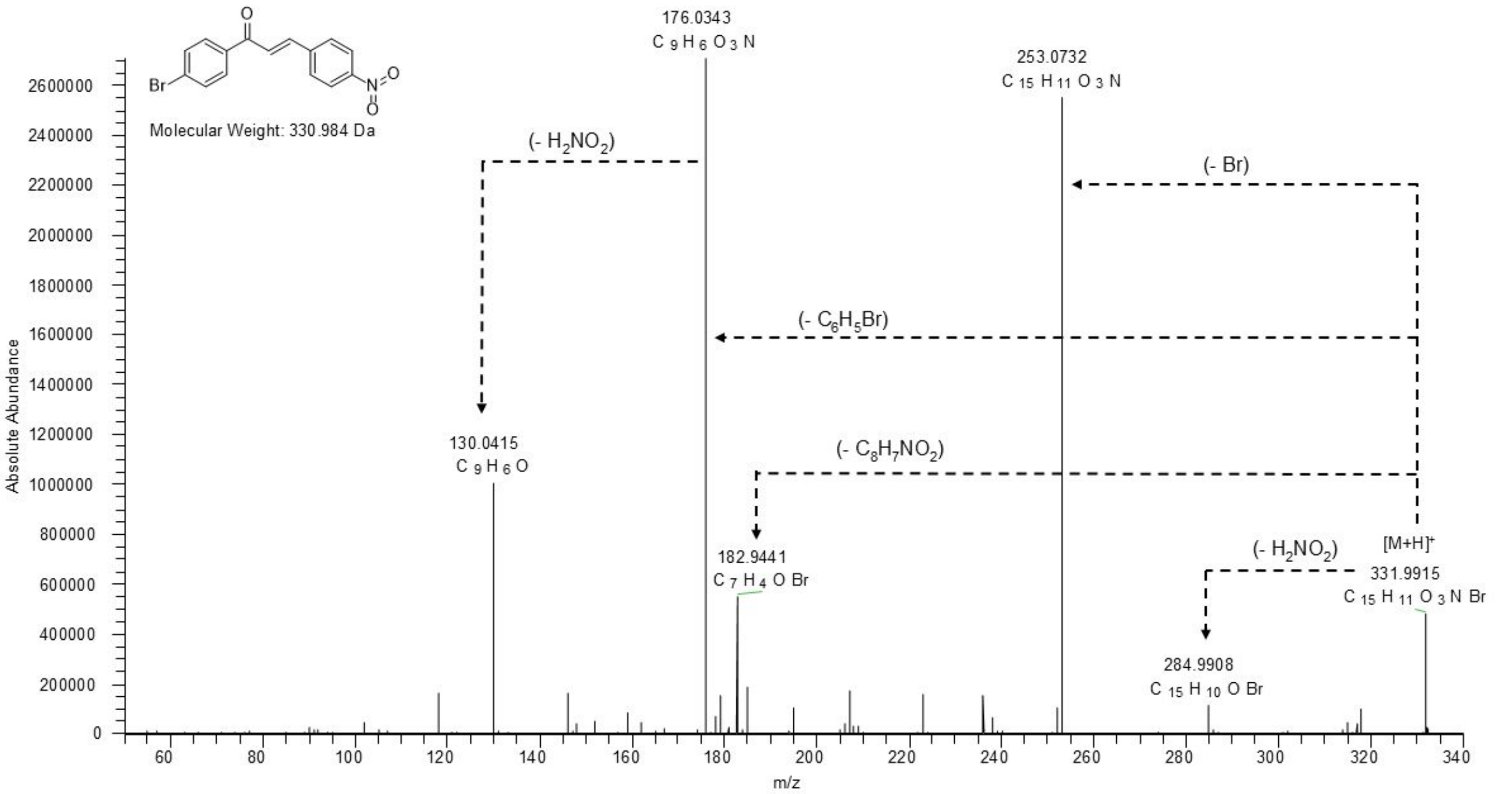

Figure S30. Fragmentation profile of sample **A10**.

- **1.5 Compound characterization data**

A1 - *(2E)-1-(4-Bromophenyl)-3-phenyl-2-propen-1-one*: $C_{15}H_{11}BrO$; Reaction Yield: 3%; Molecular Weight: 287,6 g.mol^{-1}; CAS Number: 22966-23-0; ESI-MS: $[M+H]^+$ = *m/z* 287.0062 Yellowish white solid.

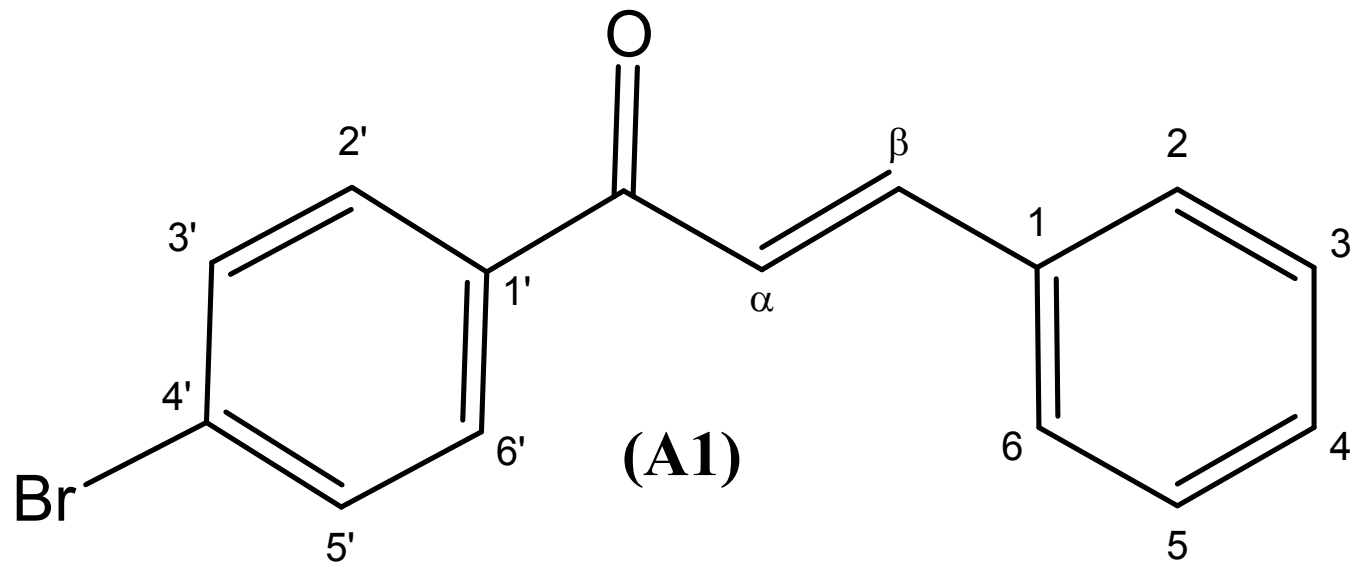

(A1)

RMN ^{1}H 500MHz $CDCl_3$ (*δ* ppm, *m*, J): 7.87 ppm (*d*, 8.6 Hz, H-2'/H-6'); 7.80 ppm (*d*, 15.5 Hz, H-β); 7.63 ppm (*d*, 8.6 Hz, H-3'/H-5'; H-2/H-6); 7.45 ppm (*d*, 15.5 Hz, H-α); 7.41 ppm (*m*, H-3/H-4/H-5).

^{13}C NMR 125MHz $CDCl_3$ (δ ppm): 189.6 ppm (C=O); 145.6 ppm (C-β); 137.2 ppm (C-1'); 134.9 ppm (C-1); 132.1 ppm (C-3'/C-5'); 131.0 ppm (C-2'/C-6'); 130.1 ppm (C-3/C-5); 129.2 ppm (C-4); 121.7 ppm (Cα).

A2 - *(2E)-1-(4-bromophenyl)-3-(4-methylphenyl)prop-2-en-1-one*: $C_{16}H_{13}BrO$; Reaction Yield: 74 %; Molecular Weight: 301.18 g.mol^{-1}; CAS Number: 130668-84-2; ESI-MS: $[M+H]^+$ = *m/z* 301.0221; Yellowish white solid.

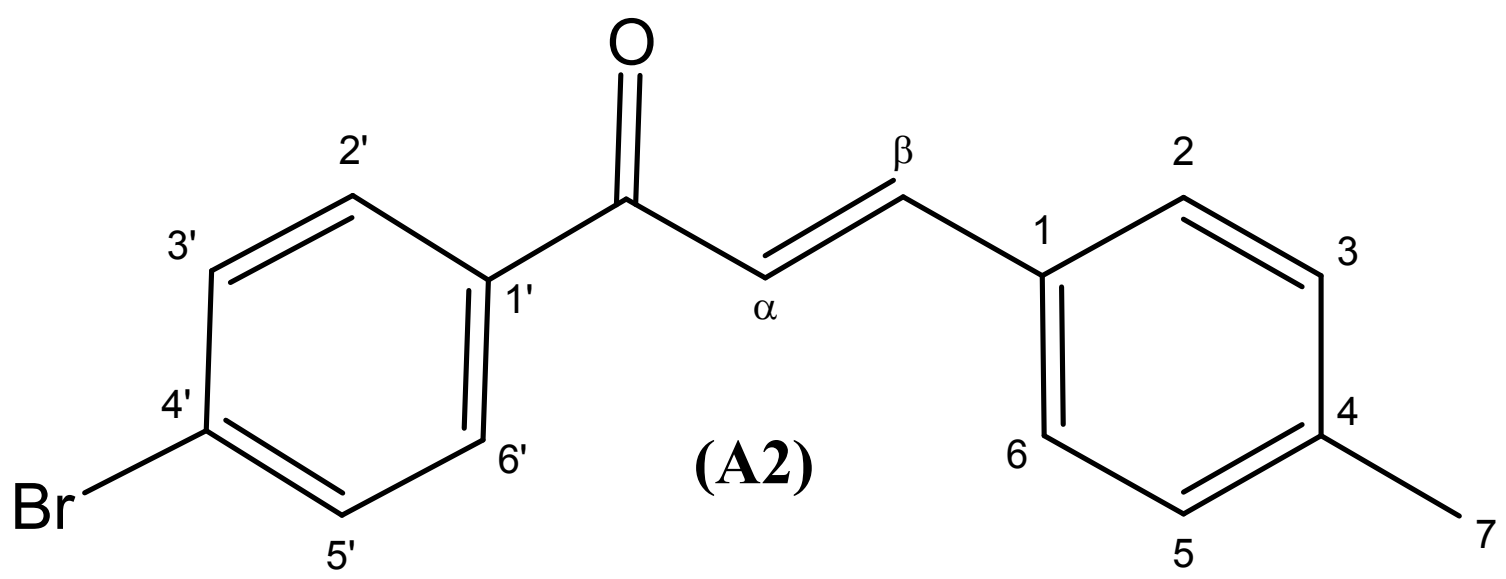

(A2)

RMN ^{1}H 500MHz $CDCl_3$ (*δ* ppm, *m*, J): 7.86 ppm (*d*, 8.6 Hz, H-2'/H-6'); 7.77 ppm (*d*, 15.5 Hz, H-β); 7.61 ppm (*d*, 8.6 Hz, H-3'/H-5';); 7.52 ppm (*d*, 8.6 Hz, H-2/H-6); 7.40 ppm (*d*, 15.5 Hz, H-α); 7.21 ppm (*d*, 8.1 Hz, H-3/H-5); 2.38 (*s*, H7)

^{13}C NMR 125MHz $CDCl_3$ (δ ppm): 189.5 ppm (C=O); 145.5 ppm (C-β); 141.4 ppm (C-1'); 137.1 ppm (C-4); 131.9 ppm (C-3'/C-5'); 130.0 ppm(C-2'/C-6'); 129.8 ppm (C-4'); 128.6 ppm (C-3/C-5); 127.7 ppm (C-2/C-6); 120.5 ppm (Cα); 21.6 ppm (C7).

A3 - *(2E)-1-(3-Bromophenyl)-3-(4-ethylphenyl)prop-2-en-1-one*: $C_{17}H_{15}BrO$; Reaction Yield: 44 %; Molecular Weight: 315.21 g.mol^{-1}; CAS Number: 126443-12-7; ESI-MS: $[M+H]^+$ = *m/z* 315.0371; Yellowish white solid.

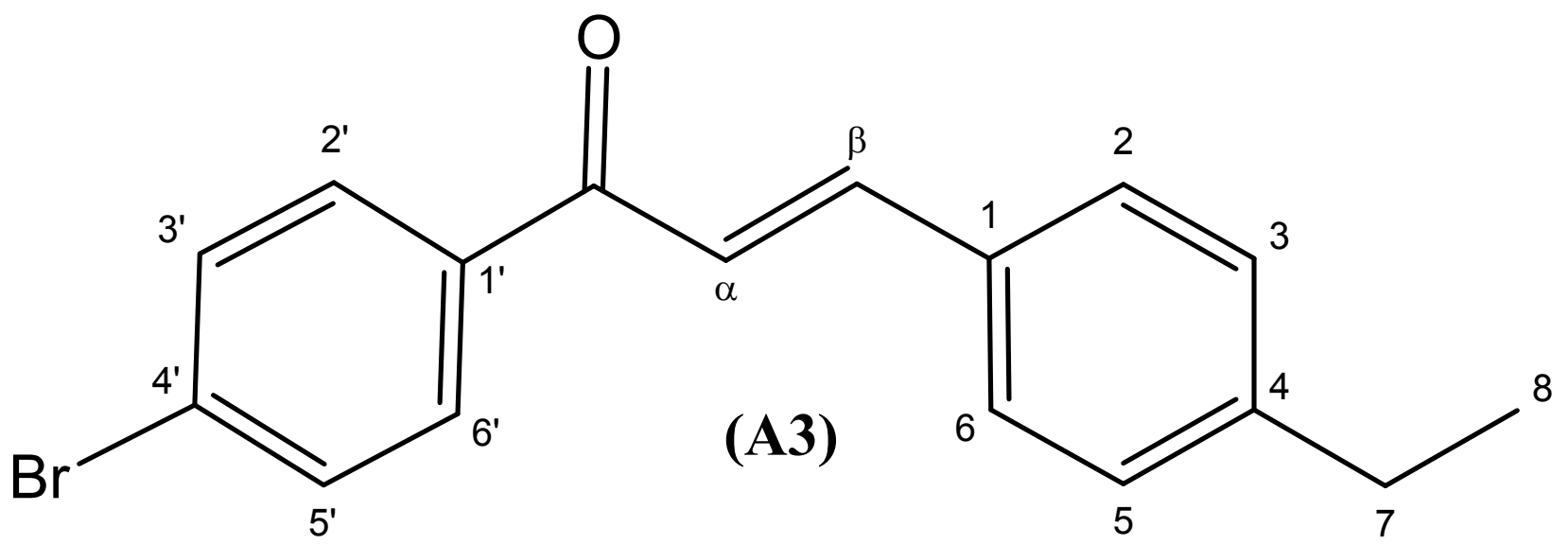

RMN ^{1}H 500MHz CDCl$_3$ (*δ* ppm, *m*, J): 7.83 ppm (*d*, 8.5 Hz, H-2'/H-6'); 7.75 ppm (*d*, 15.5 Hz, H-β); 7.59 ppm (*d*, 8.6 Hz, H-3'/H-5';); 7.51 ppm (*d*, 8.6 Hz, H-2/H-6); 7.38 ppm (*d*, 15.5 Hz, H-α); 7.21 ppm (*d*, 8.1 Hz, H-3/H-5); 2.64 ppm (*t*,7.6 H7); 1.21 ppm (*t*,7.6 Hz, H7)

^{13}C NMR 125MHz CDCl$_3$ (δ ppm): 189.5 ppm (C=O); 145.5 ppm (C-β); 141.4 ppm (C-1'); 137.1 ppm (C-4); 131.9 ppm (C-3'/C-5'); 130.0 ppm(C-2'/C-6'); 129.8 ppm (C-4'); 128.6 ppm (C-3/C-5); 127.7 ppm (C-2/C-6); 120.5 ppm (Cα); 21.6 ppm (C7).

A4 - *(2E)-3-(4-bromophenyl)-1-(4-methoxyphenyl)prop-2-en-1-one*: $C_{16}H_{13}BrO$; Reaction Yield: 44 %; Molecular Weight: 317.18 g.mol^{-1}; CAS Number: 1206687-37-7; ESI-MS: $[M+H]^+$ = *m/z* 317.0169; Yellowish white acicular solid.

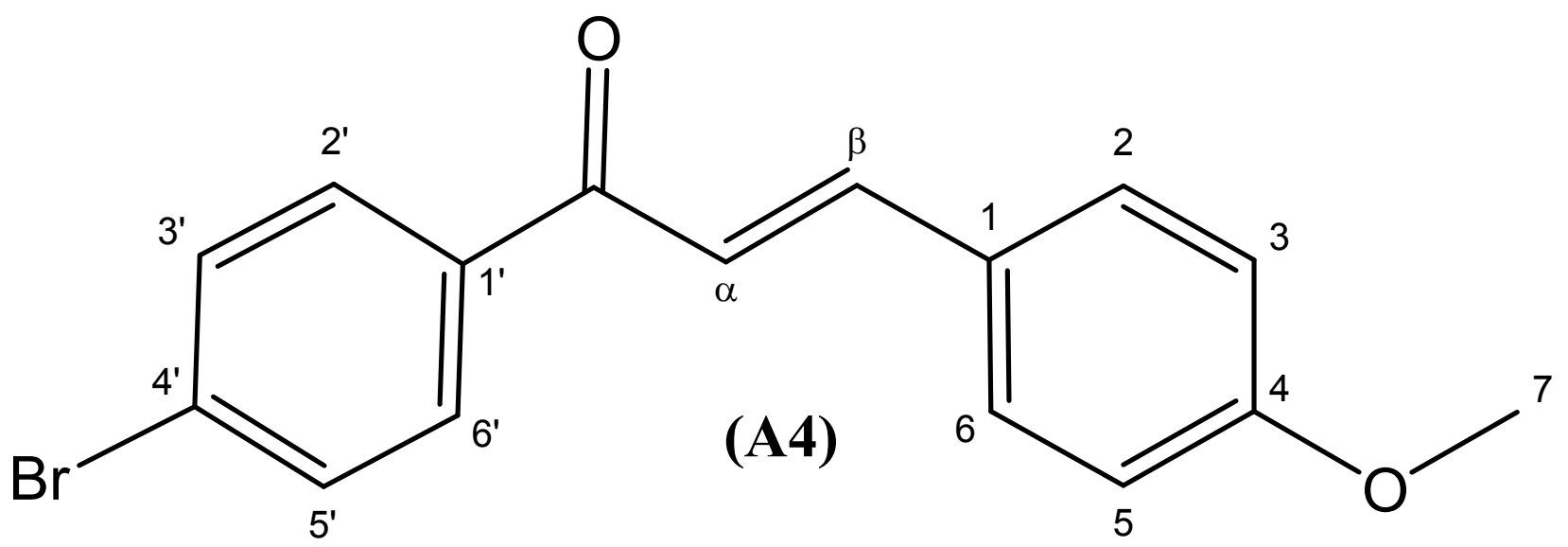

RMN ^{1}H 500MHz CDCl$_3$ (*δ* ppm, *m*, J): 7.85 ppm (*d*, 8.5 Hz, H-2'/H-6'); 7.77 ppm (*d*, 15.5 Hz, H-β); 7.61 ppm (*d*, 8.6 Hz, H-3'/H-5';); 7.58 ppm (*d*, 8.6 Hz, H-2/H-6); 7.33 ppm (*d*, 15.5 Hz, H-α); 6.92 ppm (*d*, 8.1 Hz, H-3/H-5); 3.84 ppm (*s*, H7).

^{13}C NMR 125MHz CDCl$_3$ (δ ppm): 189.6 ppm (C=O); 162.1 ppm (C-4); 145.5 ppm (C-β); 137.5 ppm (C-1'); 132.1 ppm (C-3'/C-5'); 130.6 ppm (C-2/C-6); 130.1 ppm (C-2'/C-

6'); 127.8 ppm (C-4'); 127.7 ppm (C1); 119.4 ppm (Cα); 114.8 ppm (C-3/C-5); 55.6 ppm (C7).

A5 - *(2E)-3-(4-bromophenyl)-1-(4-methoxyphenyl)prop-2-en-1-one*: $C_{17}H_{15}BrO_2$; Reaction Yield: 63 %; Molecular Weight: 331.21 g.mol^{-1}; CAS Number: 1206687-37-7; ESI-MS: $[M+H]^+$ = *m/z* 331.0325; Yelllow crystalline solid.

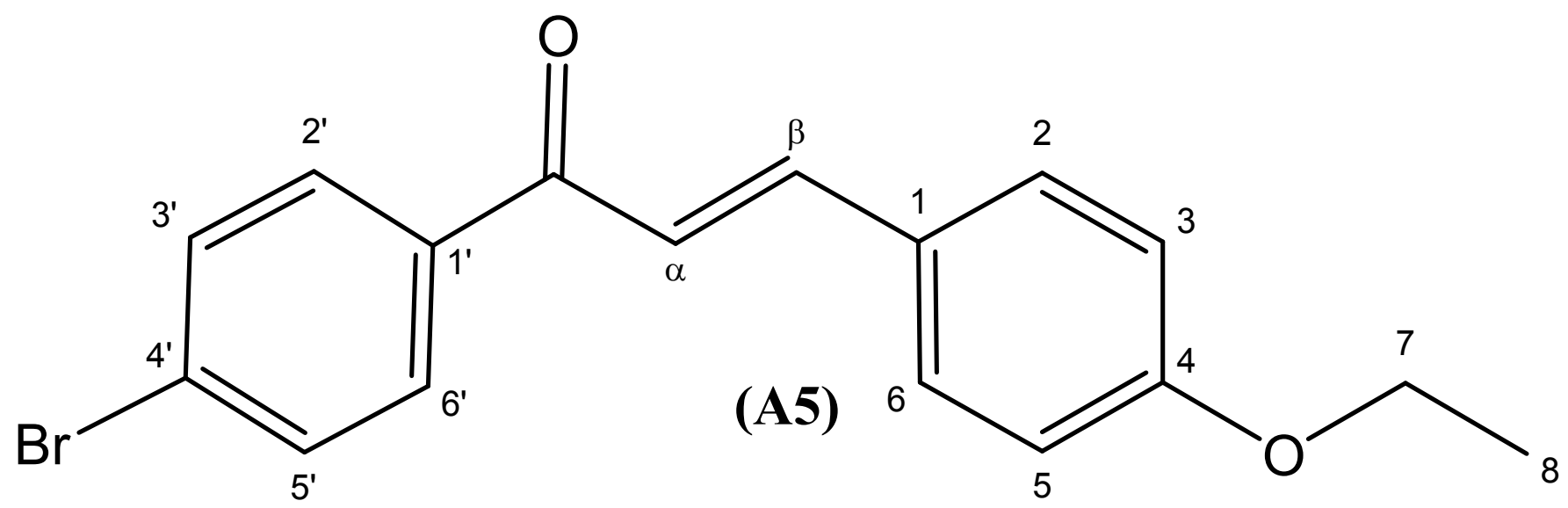

RMN ^{1}H 500MHz CDCl$_3$ (*δ* ppm, *m*, J): 7.85 ppm (*d*, 8.5 Hz, H-2'/H-6'); 7.76 ppm (*d*, 15.5 Hz, H-β); 7.61 ppm (*d*, 8.4 Hz, H-3'/H-5';); 7.57 ppm (*d*, 8.7 Hz, H-2/H-6); 7.32 ppm (*d*, 15.5 Hz, H-α); 6.92 ppm (*d*, 8.8 Hz, H-3/H-5); 4.07 ppm (*d*, 7.2 Hz, H7); 1.42 ppm (*d*, 7.1 Hz, H8).

^{13}C NMR 125MHz CDCl$_3$ (δ ppm): 189.6 ppm (C=O); 161.5 ppm (C-4); 145.6 ppm (C-β); 137.5 ppm (C-1'); 132.1 ppm (C-3'/C-5'); 130.4 ppm (C-2'/C-6'); 127.5 ppm (C-1); 119.2 ppm (Cα); 115.2 ppm (C-3/C-5); 63.9 ppm (C7); 14 ppm (C8).

A6 - *(2E)-1-(4-Bromophenyl)-3-[4-(diethylamino)phenyl]-2-propen-1-one*: $C_{17}H_{16}BrNO$; Reaction Yield: 48 %; Molecular Weight: 330.23 g.mol^{-1}; CAS Number: 131888-13-6; ESI-MS: $[M+H]^+$ = *m/z* 330.0486; Bright Yellow crystalline solid.

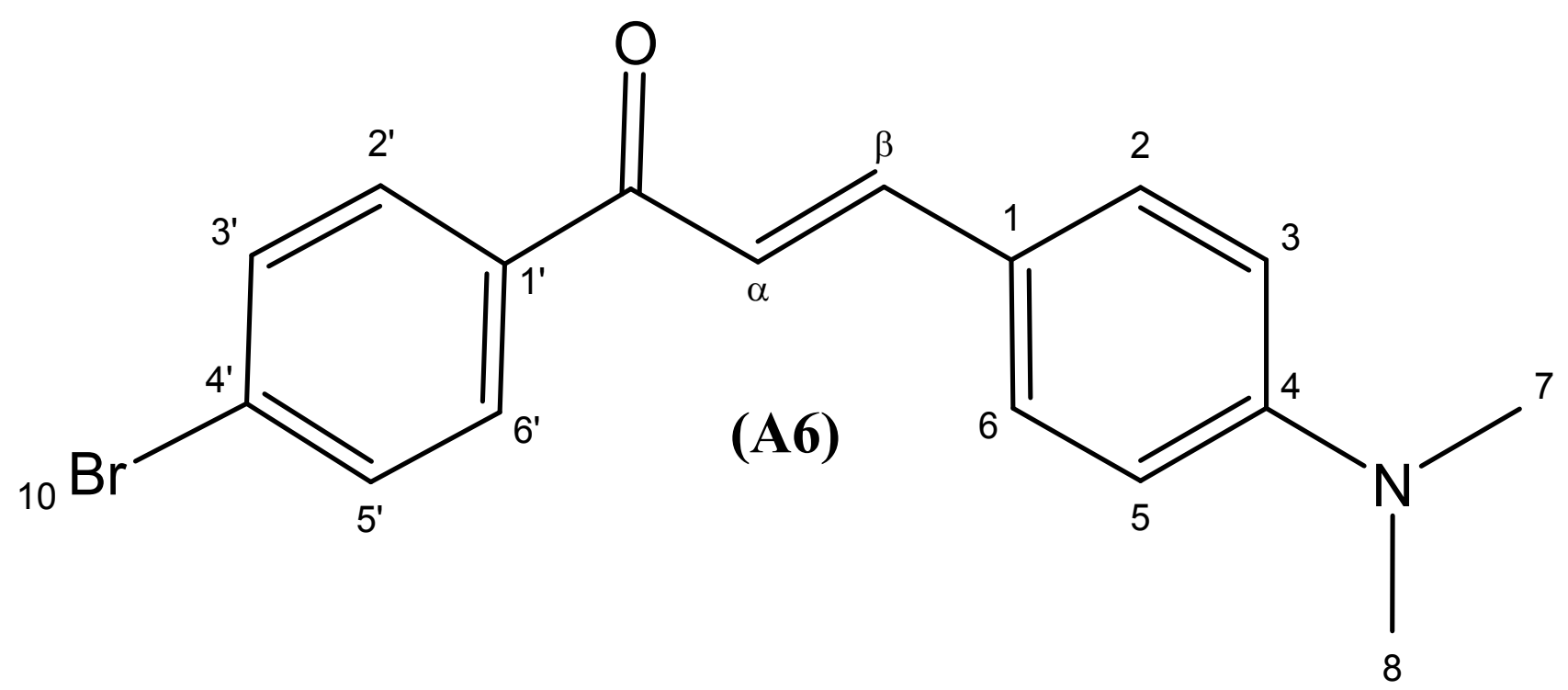

RMN ^{1}H 500MHz CDCl$_3$ (*δ* ppm, *m*, J): 7.85 ppm (*d*, 8.4 Hz, H-2'/H-6'); 7.77 ppm (*d*, 15.4 Hz, H-β); 7.60 ppm (*d*, 8.4 Hz, H-3'/H-5';); 7.52 ppm (*d*, 8.9 Hz, H-2/H-6); 7.25 ppm (*d*, 15.5 Hz, H-α); 6.67 ppm (*d*, 8.8 Hz, H-3/H-5); 3.03 ppm (*s*, H7/H8).

^{13}C NMR 125MHz $CDCl_3$ (δ ppm): 190.0 ppm (C=O); 152.4 ppm (C-4); 146.6 ppm (C-β); 138.5 ppm (C-1'); 131.9 ppm (C-3'/C-5'); 130.4 ppm (C-2'/C-6'); 127.3 ppm (C-1); 122.7 ppm (Cα); 116.4 ppm (C2-C6); 112.0 (C-3/C-5); 40.3 ppm (C7/C8).

A7 - *(2E)-1-(4-Bromophenyl)-3-(4-fluorophenyl)prop-2-en-1-one*: $C_{15}H_{10}BrFO$; Reaction Yield: 45 %; Molecular Weight: 305.15 g.mol^{-1}; CAS Number: 126473-61-8; ESI-MS: $[M+H]^+$ = *m/z* 304.9970; Yellowish-acicular crystals.

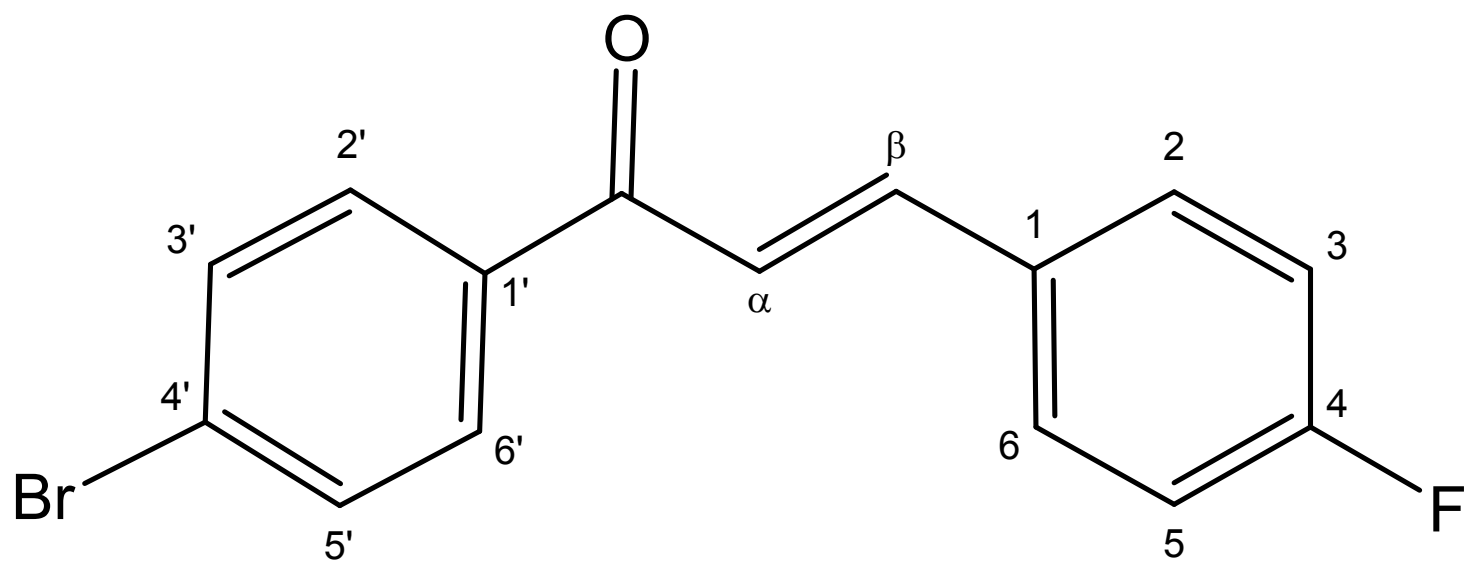

RMN ^1H 500MHz $CDCl_3$ (*δ* ppm, *m*, J): 7.86 ppm (*d*, 8.5 Hz, H-2'/H-6'); 7.76 ppm (*d*, 15.7 Hz, H-β); 7.62 ppm (*m*, 8.4 Hz, H-3'/H-5'/H-2/H-6); 7.38 ppm (*d*, 15.8 Hz, H-α); 7.10 ppm (*d*, 8.8 Hz, H-3/H-5).
^{13}C NMR 125MHz $CDCl_3$ (δ ppm): 189.4 ppm (C=O); 164.4 ppm (C-4); 144.3 ppm (C-β); 137.0 ppm (C-1'); 132.2 ppm (C-3'/C-5'); 130.6 ppm (C1); 130.2 ppm (C-2/C6); 128.2 ppm (C-2'/C-6'); 121.3 ppm (Cα); 116.0 ppm (C-3/C-5).

A8 - *(2E)-1-(4-Bromophenyl)-3-(4-chlorophenyl)prop-2-en-1-one*: $C_{15}H_{10}BrClO$; Reaction Yield: 61 %; Molecular Weight: 321.60 g.mol^{-1}; CAS Number: 126443-16-1; ESI-MS: $[M+H]^+$ = *m/z* 320.9675; Pale yellow crystals.

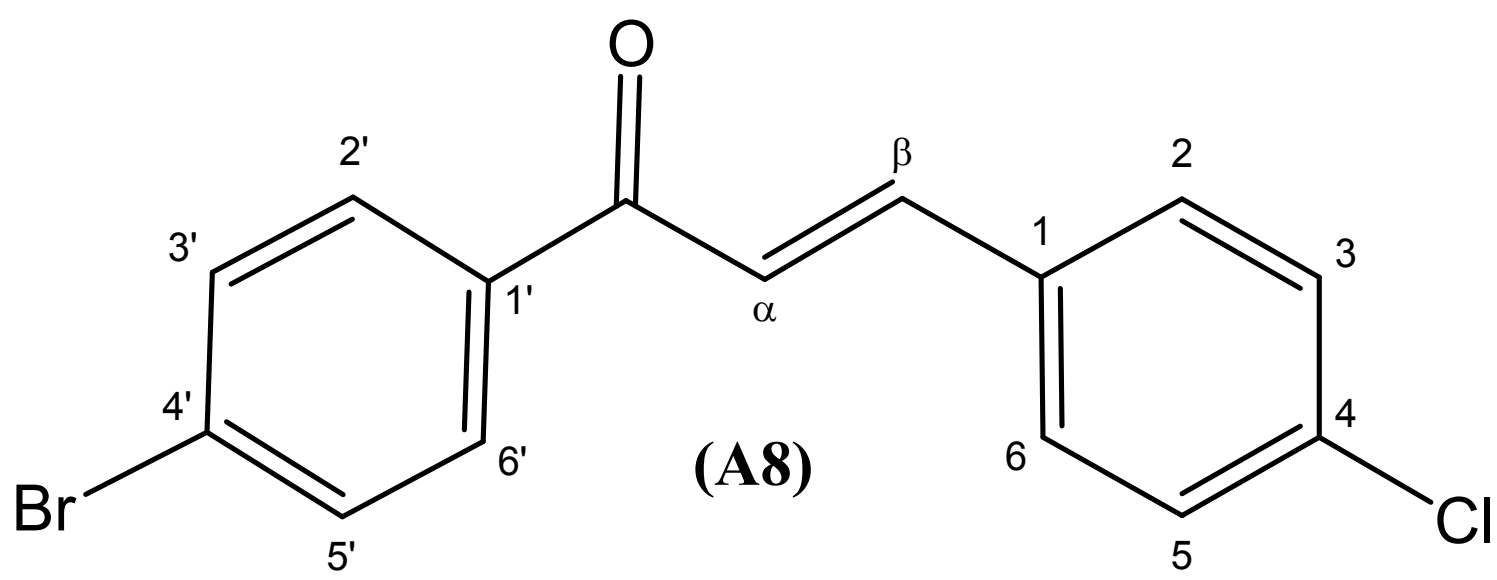

(A8)

RMN ^1H 500MHz $CDCl_3$ (*δ* ppm, *m*, J): 7.86 ppm (*d*, 8.5 Hz, H-2'/H-6'); 7.72 ppm (*d*, 15.7 Hz, H-β); 7.63 ppm (*d*, 8.6 Hz, H-3'/H-5'); 7.54 ppm (H-2/H-6); 7.48 ppm (*d*, 8.8 Hz, H-3/H-5);7.44 (*d*, 15.8 Hz, H-α).

^{13}C NMR 125MHz $CDCl_3$ (δ ppm): 189.1 ppm (C=O); 143.9 ppm (C-β); 136.7 ppm (C-1'); 133.6 ppm(C-4); 132.3 ppm (C1); 132.0 ppm (C-3'/C-5'); 130.0 ppm (C-2'/C-6'); 129.8 ppm (C-2/C6); 128.1 ppm (C4'); 125.1 ppm (C-3/C-5); 122.0 ppm (Cα).

A9 - *(2E)-1,3-Bis(4-bromophenyl)-2-propen-1-one*: $C_{15}H_{10}Br_2O$; Reaction Yield: 52 %; Molecular Weight: 366.05 g.mol^{-1}; CAS Number: 126443-21-8; ESI-MS: $[M+H]^+$ = *m/z* 366.9149; Pale yellow crystals.

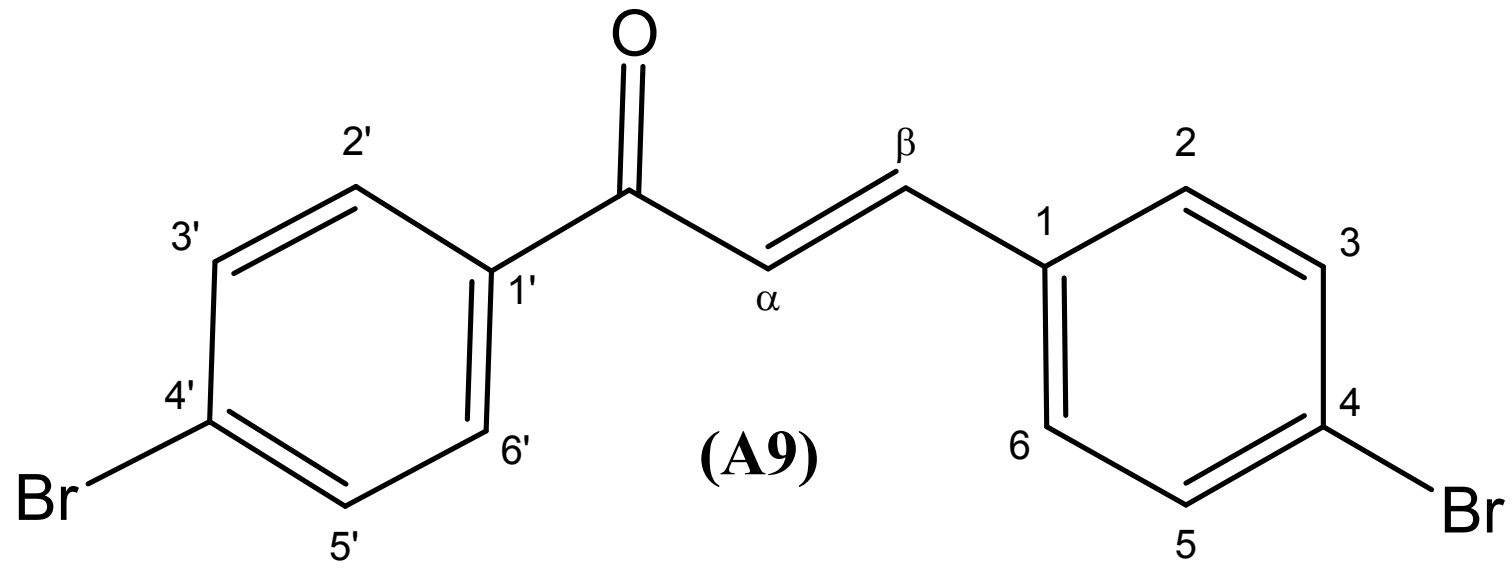

(A9)

RMN ^{1}H 500MHz $CDCl_3$ (*δ* ppm, *m*, J): 7.86 ppm (*d*, 8.7 Hz, H-2'/H-6'); 7.74 ppm (*d*, 15.6 Hz, H-β); 7.63 ppm (*d*, 8.6 Hz, H-3/H-5); 7.55 (*d*, 8.5 Hz, H-3'/H-5'); 7.42 (*d*, 15.8 Hz, H-α); 7.38 ppm (*d,* 8.6 Hz, H-2/H-6).

^{13}C NMR 125MHz $CDCl_3$ (δ ppm): 189.3 ppm (C=O); 144.1 ppm (C-β); 136.9 ppm (C-1'); 133.4 ppm (C1); 132.2 ppm (C-3'/C-5'); 130.2 ppm (C-3/C-5); 129.9 ppm (C4'); 129.5 ppm (C'-2/C'6); 128.3 ppm (C-2/C6); 122.1 ppm (C4); 120.0 ppm (Cα).

A10 - *(2E)-1-(4-Bromophenyl)-3-(4-nitrophenyl)-prop-2-en-1-one*: $C_{15}H_{10}BrNO_3$; Reaction Yield: 52 %; Molecular Weight: 332.15 g.mol^{-1}; CAS Number: 71122-94-6; ESI-MS: $[M+H]^+$ = *m/z* 331.9915; brownish-yellow acicular crystals

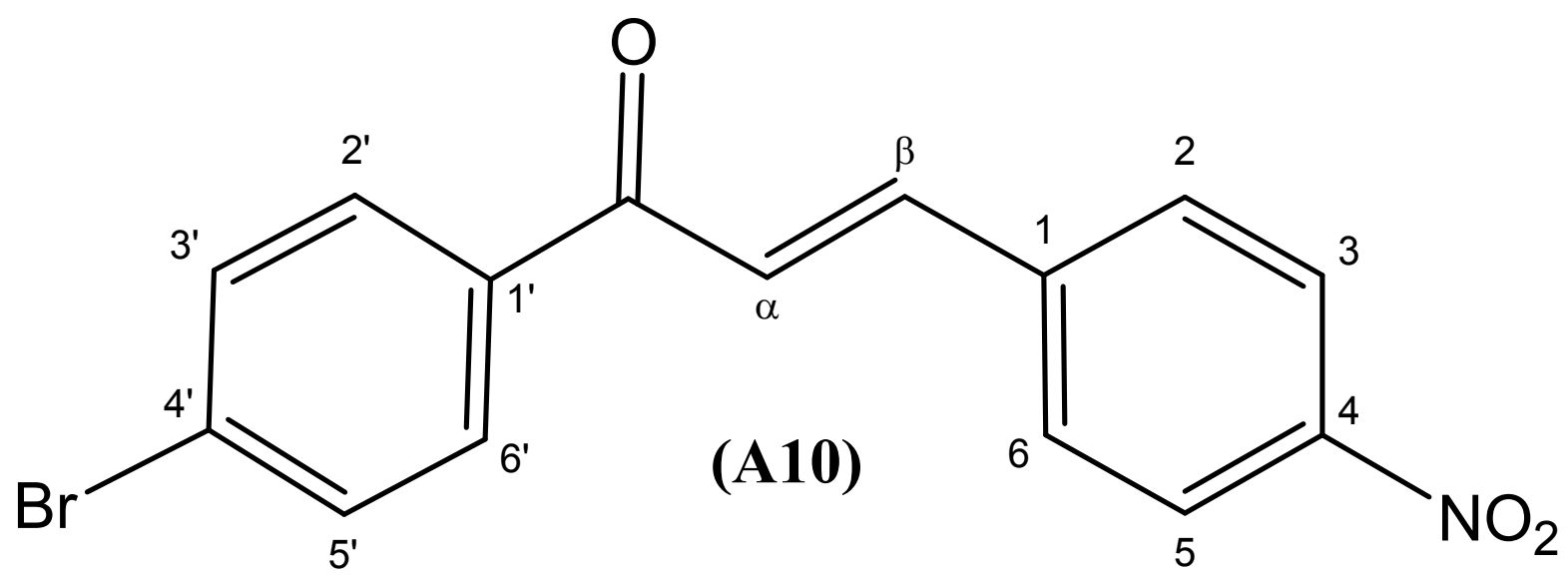

(A10)

RMN ^{1}H 500MHz $CDCl_3$ (*δ* ppm, *m*, J): 8.26 ppm (*d*, 8.6 Hz, H-3/H-5); 7.88 ppm (*d*, 8.5 Hz, H-2/H-6); 7.88 ppm (*d*, 8.7 Hz, H-2/H-6); 7.81 ppm (*d*, 15.7 Hz, H-β); 7.77 ppm (*d*, 8.7 Hz, H-2'/H-6'); 7.65 (*d*, 8.5 Hz, H-3'/H-5'); 7.57 (*d*, 15.8 Hz, H-α);.

2. Cell-based assays

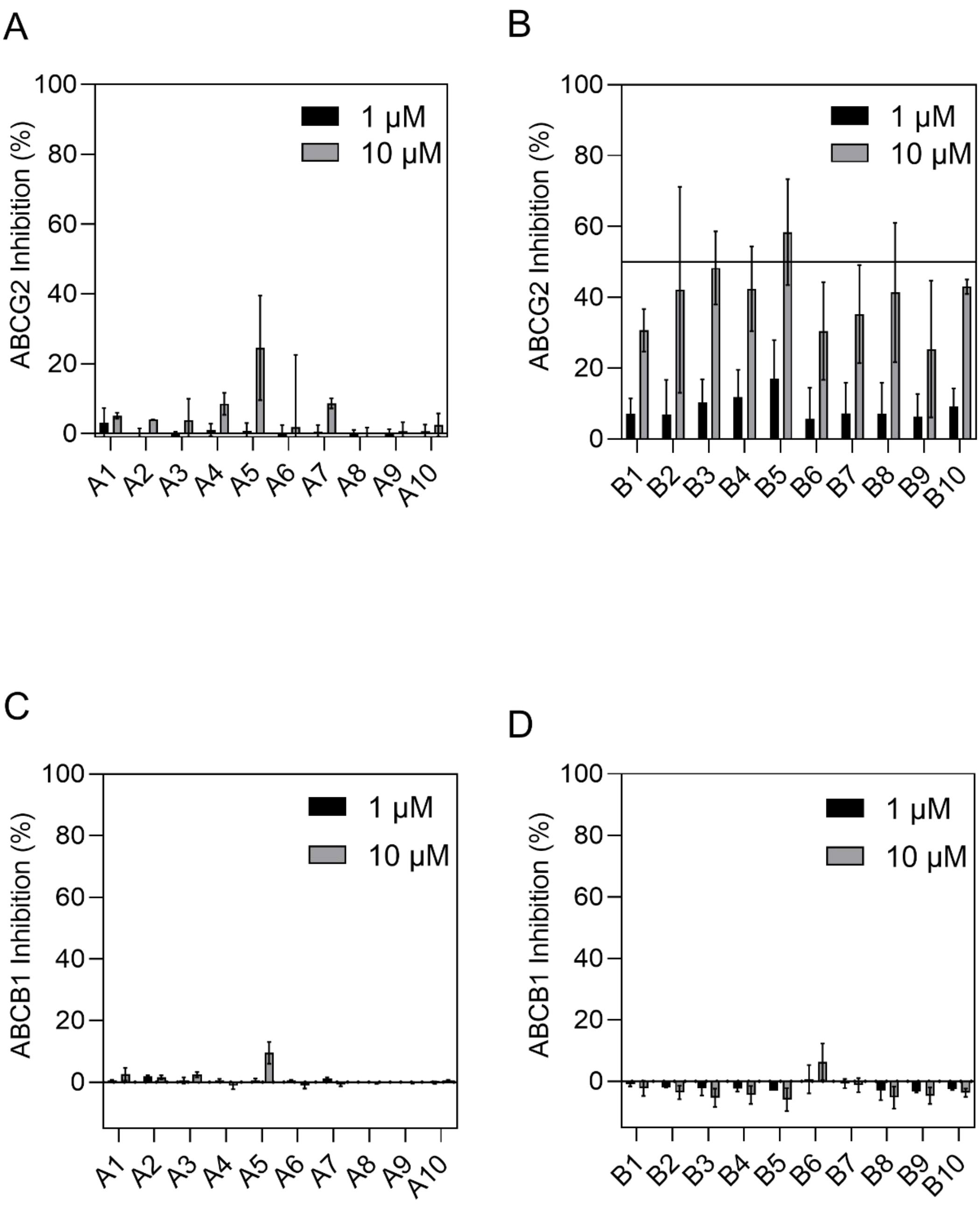

Figure S31. Comparative effects of chalcone derivatives on ABCG2- and ABCB1-mediated transport. Chalcone derivatives from the bromo-substituted series A1–A10 and the acetamide-substituted series B1–B10 were evaluated at 1 and 10 µM for their ability to inhibit ABC transporter-mediated efflux. (A) Effects of A1–A10 on ABCG2-mediated Hoechst 33342 efflux. (B) Effects of B1–B10 on ABCG2-mediated Hoechst 33342 efflux. (C) Effects of A1–A10 on ABCB1-mediated rhodamine 123 efflux. (D) Effects of B1–B10 on ABCB1-mediated rhodamine 123 efflux. ABCG2 activity was assessed in HEK293-ABCG2 cells using Hoechst 33342 (3 µM), whereas ABCB1 activity was assessed in NIH3T3-ABCB1 cells using rhodamine 123 (5 µM). The horizontal line in panel B indicates the 50% inhibition threshold used to select compounds for further characterization. Data are presented as mean ± SD from at least three independent experiments.

Table S1. ABCB1 inhibition activity of chalcones.

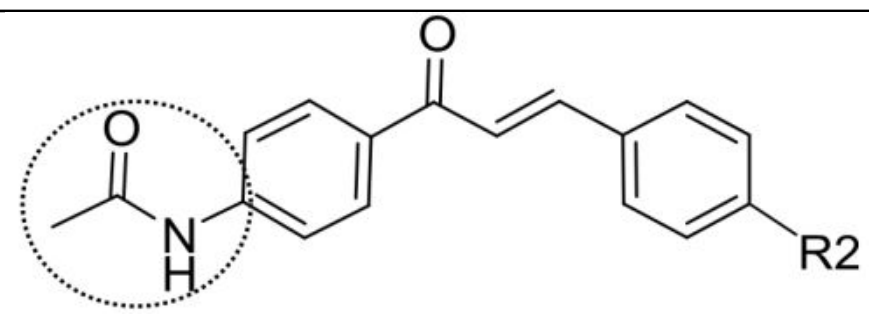

Chalcone	R2	ABCB1 Inhibition (%)	
		1 µM	10 µM
B1	H	-0.88 ± 0.73	-2.28 ± 2.49
B2	CH_3	-1.89 ± 0.16	-3.63 ± 2.20
B3	CH_2CH_3	-2.25 ± 2.29	-5.38 ± 2.94
B4	OCH_3	-2.40 ± 0.93	-4.43 ± 2.93
B5	OCH_2CH_3	-3.06 ± 0.01	-5.94 ± 3.74
B6	NH_2	0.69 ± 4.6	6.32 ± 5.92
B7	F	-0.73 ± 1.4	-1.27 ± 2.29
B8	Cl	-3.01 ± 3.08	-5.31 ± 3.59
B9	Br	-3.30 ± 0.35	-4.70 ± 2.64
B10	NO_2	-2.57 ± 0.31	-3.73 ± 1.36

Description. Inhibitory effects of chalcone derivatives on ABCB1-mediated transport. Chalcone derivatives and their corresponding R2 substituents are shown in the first and second rows, respectively. ABCB1 inhibition at 1 and 10 µM is presented in the third and fourth rows. Transport activity was assessed by flow cytometry in NIH3T3 cells overexpressing ABCB1 using rhodamine 123 (5 µM) as the fluorescent substrate. Inhibition was normalized to that produced by the reference ABCB1 inhibitor elacridar (1 µM), defined as 100%. Data are presented as mean ± SD from at least three independent experiments. Negative values reflect experimental variation below the untreated control and were interpreted as no detectable inhibition.

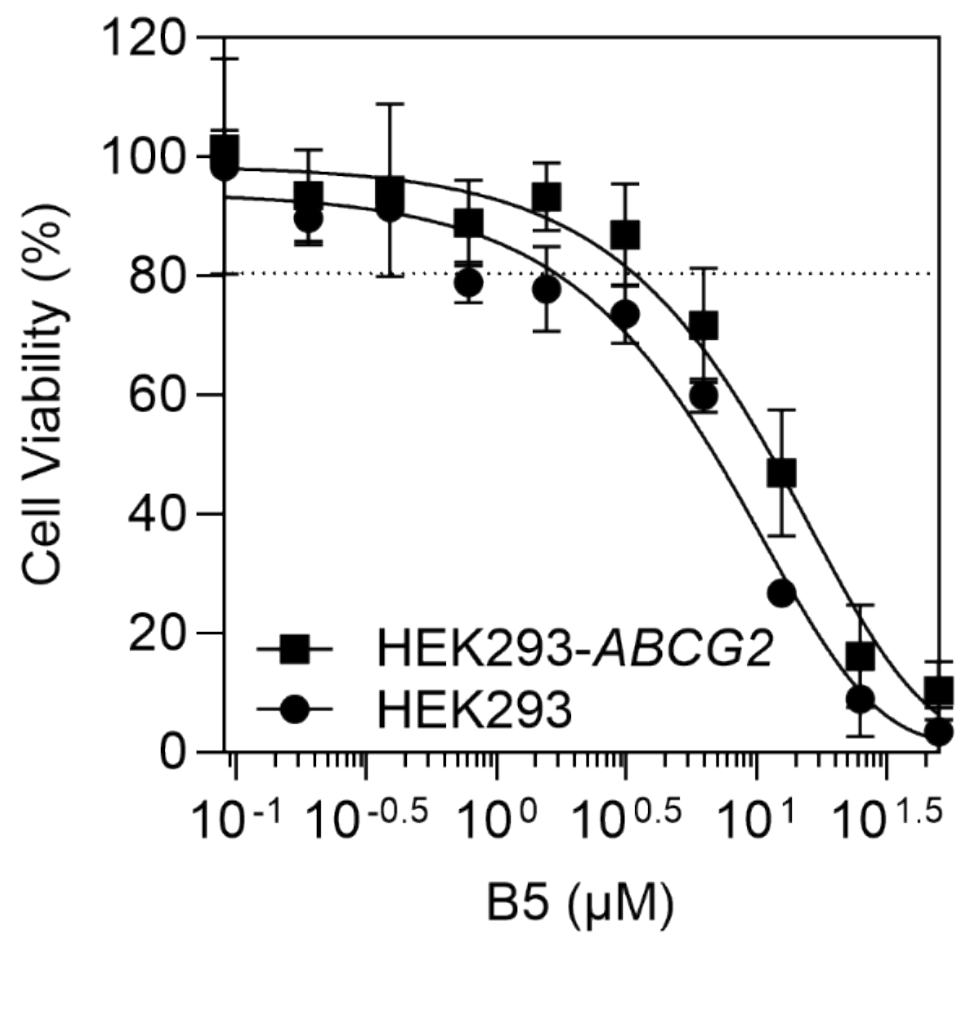

Figure S32 - Cytotoxicity assay of chalcone **B5** in HEK293 and HEK293-*ABCG2* cell lines. The cell viability was assessed using the MTT assay across a concentration range of 0.9 µM to 50 µM. The experiments were performed in at least triplicate, and data were analyzed and plotted using GraphPad Prism 9.5.0. Results are expressed as mean ± standard deviation (SD) of at from at least

three independent replicates. The dotted line represents 80% viability. Cytotoxicity of B5 in HEK293-*ABCG2* and parental cell.

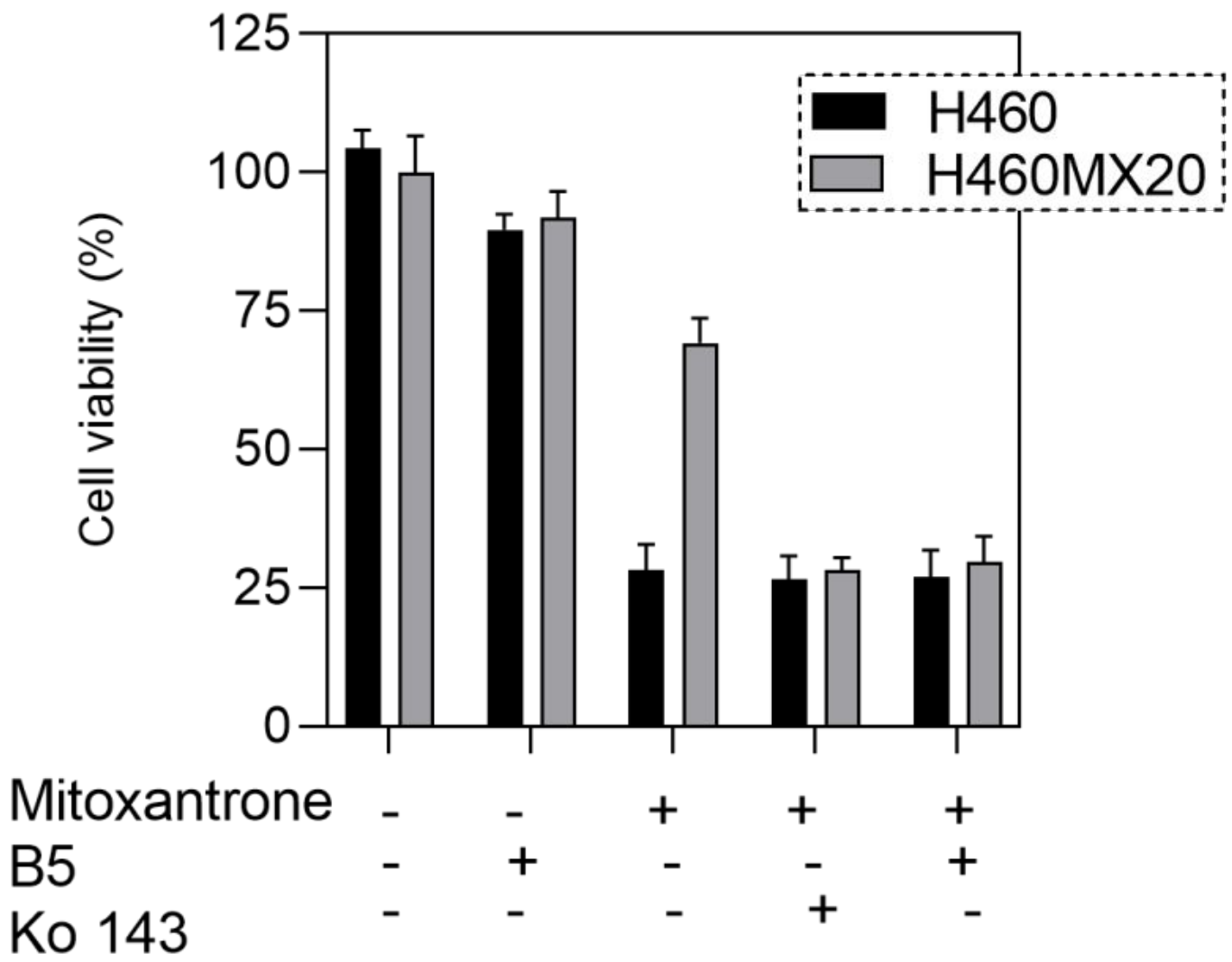

Figure S33. Reversal of mitoxantrone resistance by B5 in parental H460 and ABCG2-overexpressing H460MX20 cells. Cells were treated with mitoxantrone (0.5 µM), B5 (2.5 µM), or the reference ABCG2 inhibitor Ko143 (1 µM), either alone or in the indicated combinations, and cell viability was assessed. Data are presented as mean ± SD from at least three independent experiments.

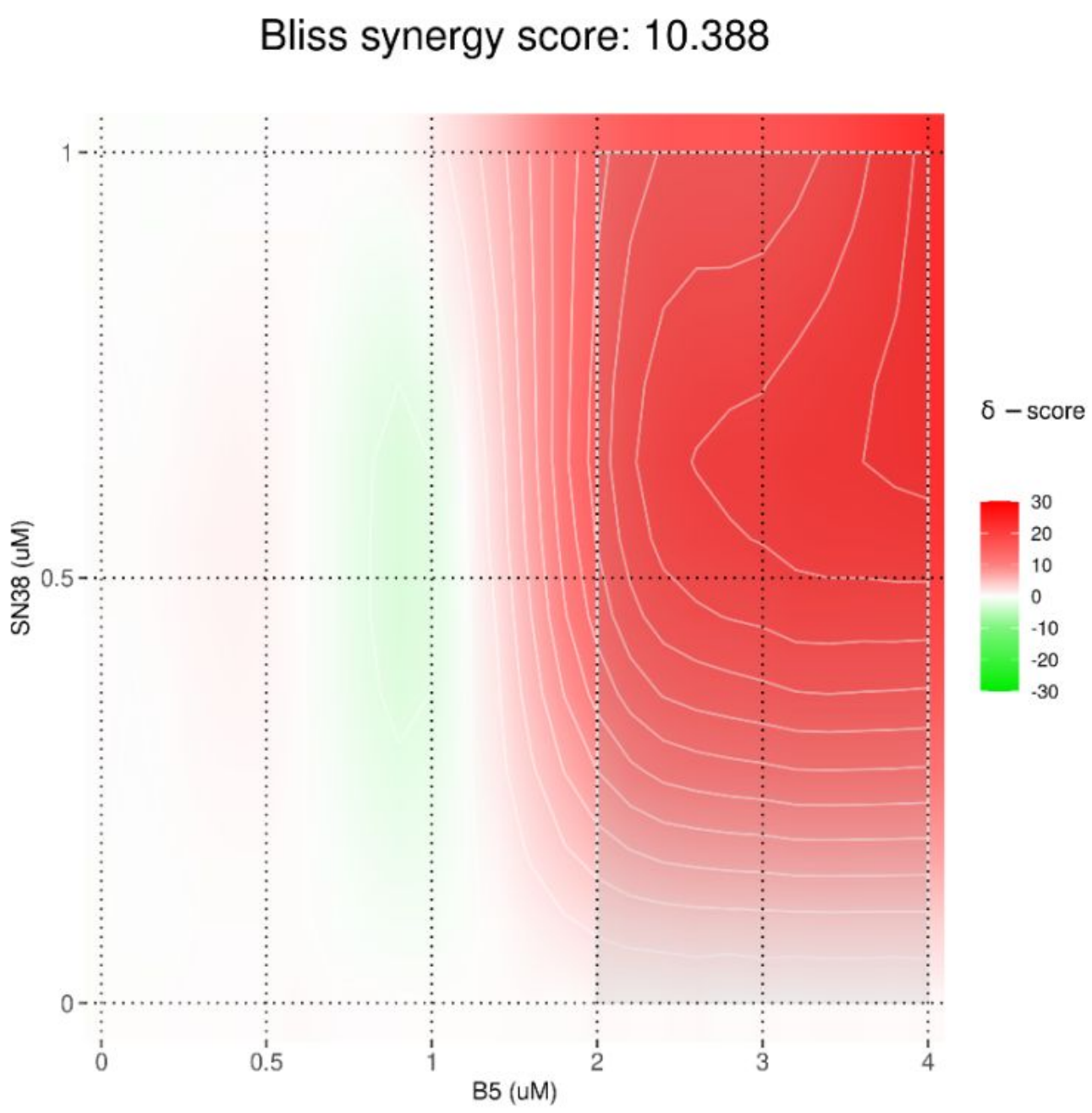

Figure S34. Bliss synergy analysis of the combination of B5 and SN-38. Two-dimensional Bliss synergy map for the combination of **B5** and SN-38. Cells were treated with increasing concentrations of **B5** (0–4 µM) and SN-38 (0–1 µM), alone or in combination, and the interaction was evaluated using the Bliss independence model. The color scale represents the Bliss δ-score, with positive values shown in red indicating synergy, values close to zero indicating an additive interaction, and negative values shown in green indicating antagonism. Contour lines represent

interpolated δ-score levels across the concentration matrix, whereas the dotted lines indicate the experimentally tested concentrations. The overall Bliss synergy score was 10.388, consistent with a modest synergistic interaction between **B5** and SN-38 under the tested conditions.

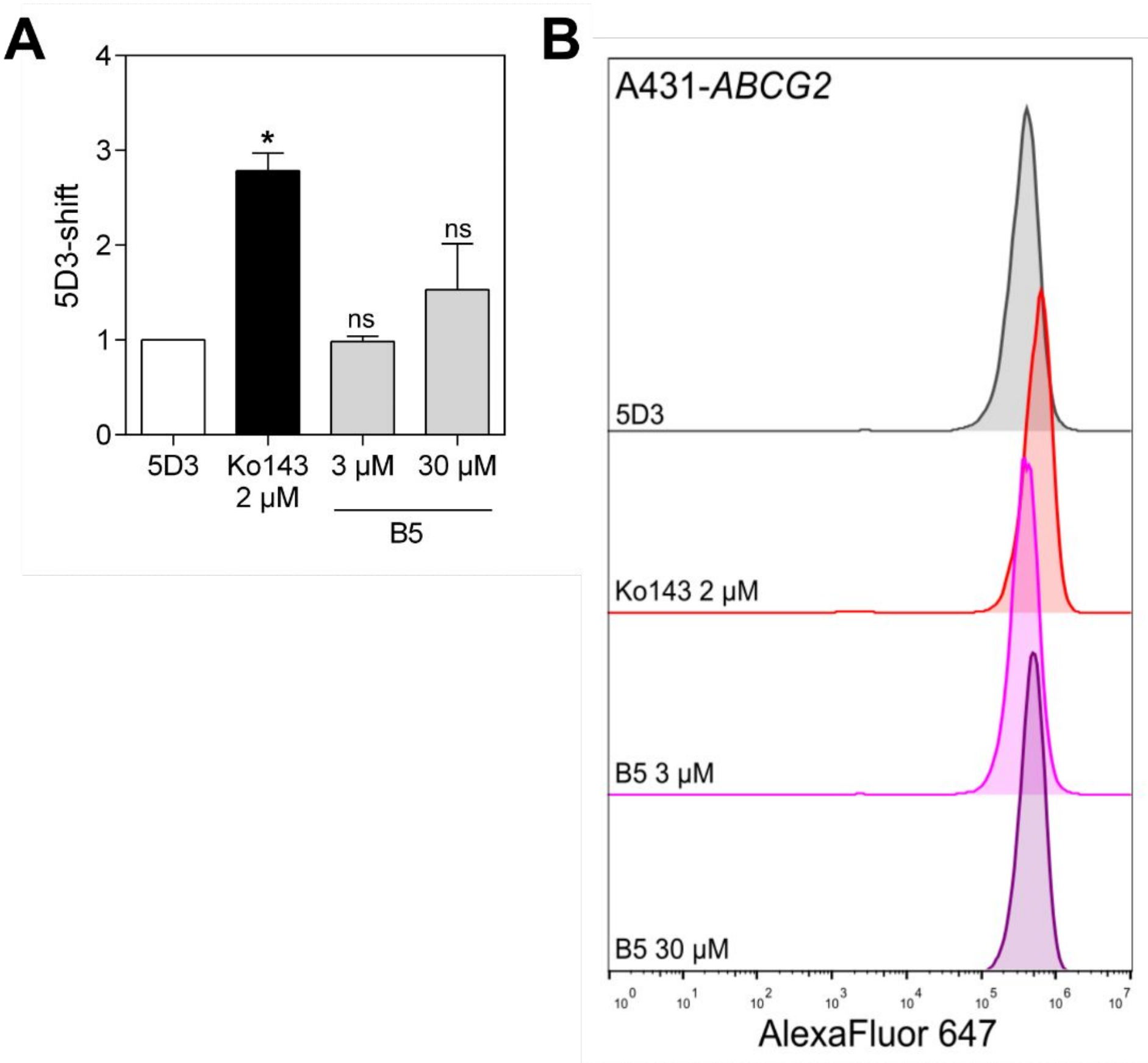

Figure S35. Effect of B5 on 5D3 binding to ABCG2. Experiments were carried out in A431 overexpressing ABCG2 cell line. Cells were pre-incubated with the reference inhibitor Ko143 (2 µM) or the chalcone **B5** (3 and 30 µM) for 30 minutes at 37°C, followed by the addition of 5D3-A647 (2.5%µg/mL) for 30 minutes. After incubation, cells were washed and stained with propidium iodide (PI, 5%µg/mL). ABCG2 conformational changes were evaluated by flow cytometry. (A) Data represents the mean ± SD of three independent experiments. Statistical differences were tested using one-way ANOVA and Tukey's post hoc test. Significative difference between control and the indicated compound with p-value * < 0.05. (B) The histograms represent one out of three independent measurements.

- ## 3 Computational analyses

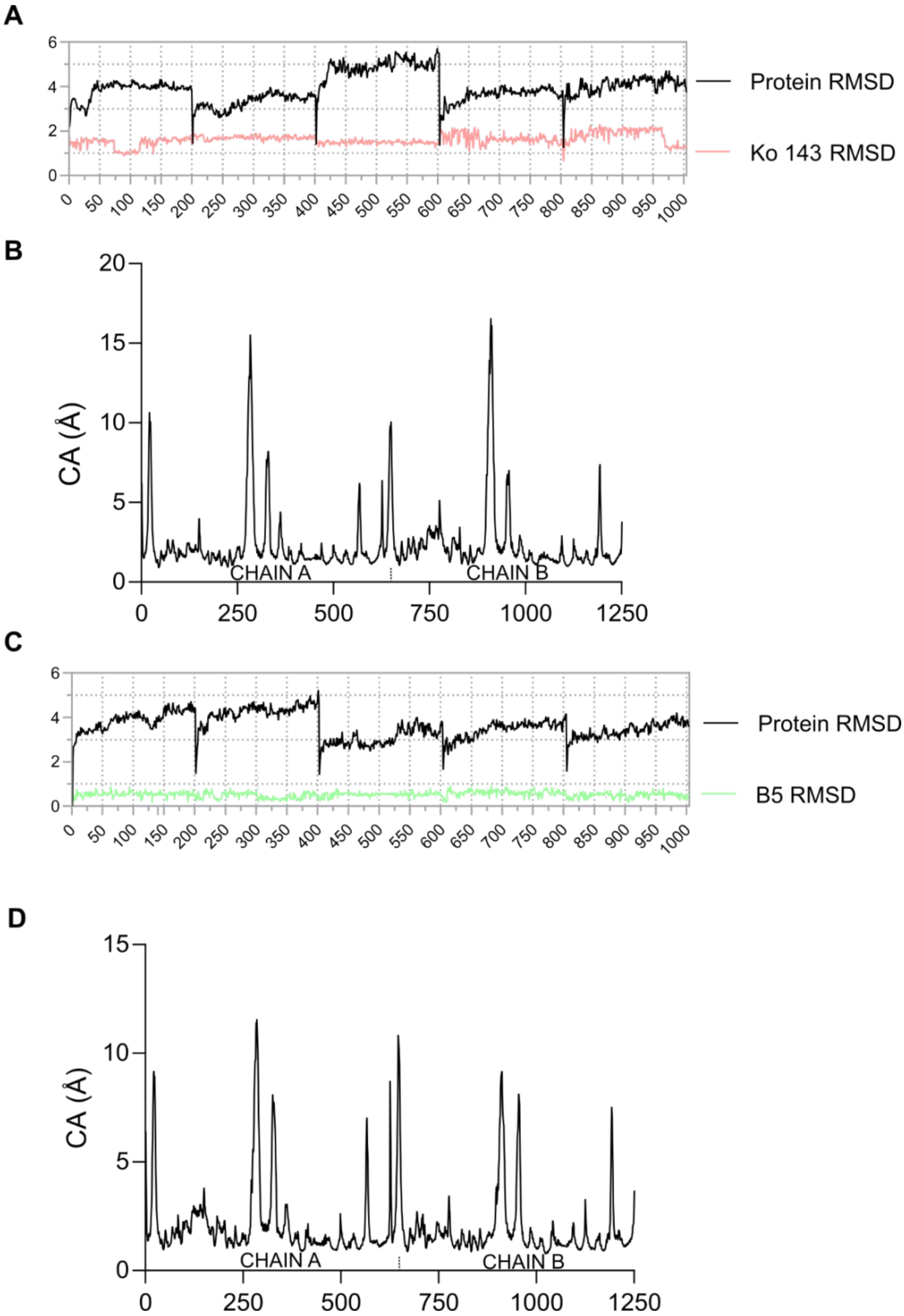

Figure S36. Conformational stability and residue flexibility of the ABCG2 complexes with Ko143 and B5. Protein backbone and ligand root-mean-square deviation (RMSD) profiles are shown for the ABCG2–Ko143 (A) and ABCG2–B5 (C) complexes. Root-mean-square fluctuation (RMSF)

profiles of the Cα atoms in chains A and B are shown for the ABCG2–Ko143 (B) and ABCG2–B5 (D) complexes. Ko143 and B5 are represented in light pink and green, respectively, while the protein backbone is shown in black. For each complex, five independent 200 ns molecular dynamics simulations were concatenated for analysis, corresponding to an aggregate simulation time of 1 µs. RMSF peaks indicate protein regions with increased conformational flexibility. Molecular dynamics simulations were performed using Desmond within the Schrödinger Suite (version 2024-2).

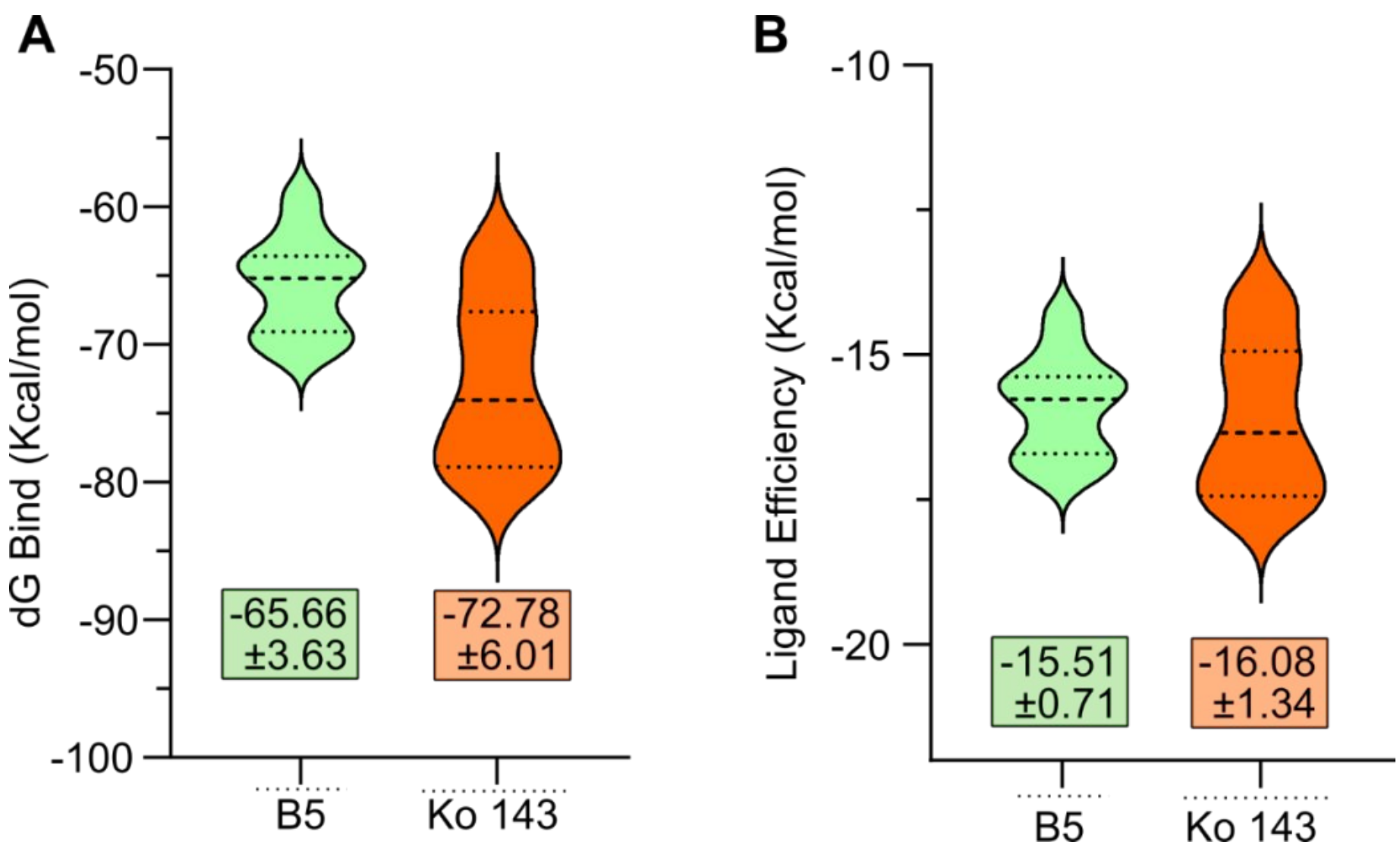

Figure S37. MM/GBSA binding free energy and ligand efficiency of B5 and Ko143. Violin plots show the distributions of the estimated binding free energy (ΔG_bind; kcal mol^{-1}) (A) and ligand efficiency (LE; kcal mol^{-1} per heavy atom) (B) for B5 and Ko143. Plots were generated using GraphPad Prism version 9.5.

Table S2. Hydrophobic frequency.

ABCG2	CHAIN A					CHAIN B						
	**Ala 397**	**Val 546**	**Phe 439**	**Val 442**	**Val 401**	**Phe 432**	**Phe 439**	**Val 442**	**Val 546**	**Met 549**	**Ile 543**	**Leu 539**
**Ko143**	10.9		44	14.1	12.2	15.2	16.8	11.6	49.5	10.9	12.6	13.1
**B5**		64.6	56.4				76.7		59.5			

Description: The table represents the Hydrophobic interaction frequencies of both inhibitors, Ko143 and **B5** with the amino acid residues from both chains A, left side and chain B right side. The data resulted from 1 µs simulation (five independent 200 ns simulation) per replica. A cutoff of 10% interaction was established. The frequency is represented in (%).

Table S3. Pi interactions frequency.

ABCG2	CHAIN A	CHAIN B

	Phe 439	Phe 439
Ko143	40.9	
B5	47.3	11.6

Description: The table represents the π-mediated interaction frequencies of both inhibitors, Ko143 and **B5** with the amino acid residues from both chains A, left side and chain B right side. The data resulted from 1 μs simulation (five independent 200 ns simulation) per replica. A cutoff of 10% interaction was established. The frequency is represented in (%).

Table S4. Hydrogen bounds interaction.

ABCG2	CHAIN A	CHAIN B
	Gln 398	Thr 542
Ko143	41.2	
B5		96.9

Description: The table represents the Hydrogen-bond interaction frequencies of both inhibitors, Ko143 and **B5** with the amino acid residues from both chains A, left side and chain B right side. The data resulted from 1 μs simulation (five independent 200 ns simulation) per replica. A cutoff of 10% interaction was established. The frequency is represented in (%).

Table S5. Comparative molecular docking of **B5** and tariquidar within the transmembrane drug-binding cavity of human ABCB1.

Compound	Classification in the analysis	docking score	Relative interpretation
Tariquidar	Reference ABCB1 inhibitor	−10.925	More favorable predicted interaction
**B5**	Investigated chalcone	−7.659	Less favorable predicted interaction than tariquidar

Description: Docking was performed using the tariquidar-bound human ABCB1 structure (PDB ID: 7A6E) and the same protein/ligand preparation, grid, and scoring parameters for both compounds. More negative docking scores indicate a more favorable predicted interaction within the applied docking protocol. Docking scores are comparative computational estimates and should not be interpreted as direct measurements of binding affinity or inhibitory potency.

References

(1) Pelosi, A. G.; Silveira-Alves, E.; Cocca, L. H. Z.; Valverde, J. V.; Oliveira, G. R.; da Silva, D. L.; De Boni, L.; Gonçalves, P. J.; Mendonca, C. R. Two-Photon Absorption and Multiphoton Excited Fluorescence of Acetamide-Chalcone Derivatives: The Role of Dimethylamine Group on the Nonlinear Optical and Photophysical

Properties. *Molecules* **2023**, *28* (4), 1572. https://doi.org/10.3390/molecules28041572.

(2) Mukhtar, S. S.; Morsy, N. M.; Hassan, A. S.; Hafez, T. S.; Hassaneen, H. M.; Saleh, F. M. A Review of Chalcones: Synthesis, Reactions, and Biological Importance. *Egyptian Journal of Chemistry* **2022**, *65* (8), 379–395. https://doi.org/10.21608/ejchem.2022.112735.5125.
